# Scaffolding-dependent CASP1 constrains excessive cell-intrinsic inflammatory signaling in leukemia

**DOI:** 10.1016/j.chembiol.2025.12.002

**Published:** 2026-01-06

**Authors:** Emma E. Uible, Issac Choi, Courtnee A. Clough, Aishlin Hassan, Annabelle J. Anandappa, Julianna Fisher, Bibek Karki, Kathleen Hueneman, Kwangmin Choi, Eric J. Vick, William Seibel, Kenneth D. Greis, Lynn Lee, Courtney Jones, Timothy M. Chlon, Jorge Henao-Mejia, Chandrashekhar Pasare, John T. Cunningham, Andrew G. Volk, Daniel T. Starczynowski

**Affiliations:** 1 Department of Cancer Biology, University of Cincinnati, Cincinnati, OH, USA; 2 Division of Experimental Hematology and Cancer Biology, Cincinnati Children’s Hospital, Cincinnati, OH, USA; 3 Division of Hematology & Oncology, University of Cincinnati, Cincinnati, OH, USA; 4 University of Cincinnati Cancer Center, Cincinnati, OH, USA; 5 Division of Oncology, Cincinnati Children’s Hospital, Cincinnati, OH, USA; 6 Department of Pediatrics, University of Cincinnati, Cincinnati, OH, USA; 7 Department of Pathology and Laboratory Medicine, Perelman School of Medicine, University of Pennsylvania, Philadelphia, PA, USA; 8 Institute for Immunology and Immune Health (I3H), Perelman School of Medicine, University of Pennsylvania, Philadelphia, PA, USA; 9 Division of Protective Immunity, Department of Pathology and Laboratory Medicine, Children’s Hospital of Philadelphia, University of Pennsylvania, Philadelphia, PA, USA; 10 Division of Immunobiology, Cincinnati Children’s Hospital, Cincinnati, OH, USA; 11 Lead contact

## Abstract

Caspase-1 (CASP1) is best known for regulating IL-1β processing and pyroptosis; however, its role in leukemia has not been clearly defined. Here, we show that loss of CASP1 impairs leukemic cell growth, drives differentiation, and reduces leukemic burden *in vivo*, independent of its CASP1 protease activity. Instead, CASP1 functions as a scaffolding hub, controlling nuclear factor kappa-light-chain-enhancer of activated B cell (NF-κB) signaling via its interaction with raptor (RPTOR), a component of mTORC1. Deletion of CASP1 or disruption of its CARD domain induces excessive NF-κB activity and impairs leukemic cell function. We further developed a proteolysis-targeting chimera (PROTAC) degrader that selectively depletes Pro-CASP1 and suppresses leukemic cells. These findings reveal CASP1 as a regulator of mTORC1-NF-κB signaling in leukemia and highlight its scaffolding activity as a therapeutic vulnerability.

## In brief

Uible et al. revealed a non-catalytic role for CASP1 in leukemia. Acting as a scaffolding hub for mTORC1-NF-κB signaling through RPTOR, CASP1 was essential for leukemic cell function. A CASP1-targeted PROTAC exposed this scaffolding activity as a therapeutic vulnerability in myeloid malignancies.

## Graphical abstract

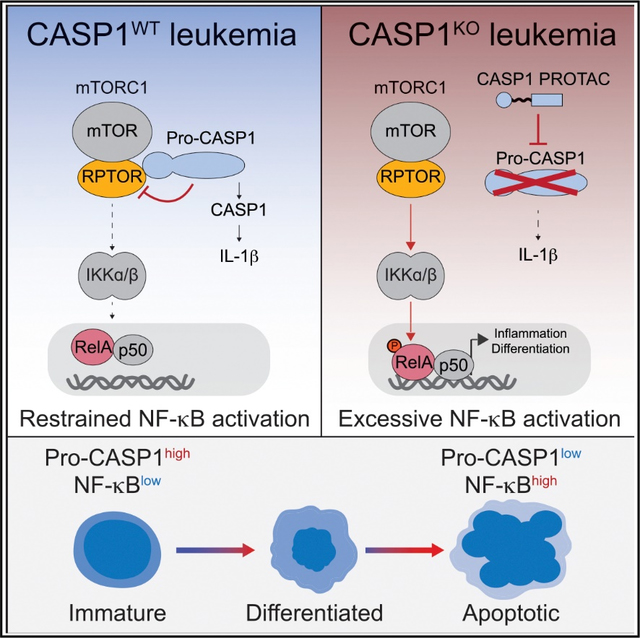

## INTRODUCTION

Leukemia remains a difficult-to-treat group of blood cancers, where biological complexity and an incomplete understanding of disease mechanisms continue to limit the impact of recent advances in targeted therapy. Among these, acute myeloid leukemia (AML) and myelodysplastic syndromes (MDSs) remain particularly challenging, as most standard-of-care therapies have remained unchanged for decades,^1^ and many patients are ineligible for curative bone marrow (BM) transplantation, leading to dismal survival rates.^1,2^ MDS and AML arise from mutant hematopoietic stem and progenitor cells (HSPCs).^3^ A hallmark of MDS/AML is dysregulated innate immune signaling and inflammation.^4–6^ Interleukin-1β (IL-1β), a central mediator of inflammatory responses, also regulates hematopoiesis by promoting granulopoiesis and myeloid differentiation under acute conditions.^7–10^ However, chronic IL-1β signaling can promote leukemic cell expansion and clonal hematopoiesis.^11–15^ Clinical studies show variable IL-1β levels in MDS/AML patient plasma, and therapies targeting IL-1β or its downstream pathways have shown limited responses in hematologic malignancies.^16–18^ This suggests that while IL-1β is involved in MDS/AML pathogenesis, it may not be the primary driver of leukemic cell function.

**Figure 1.**
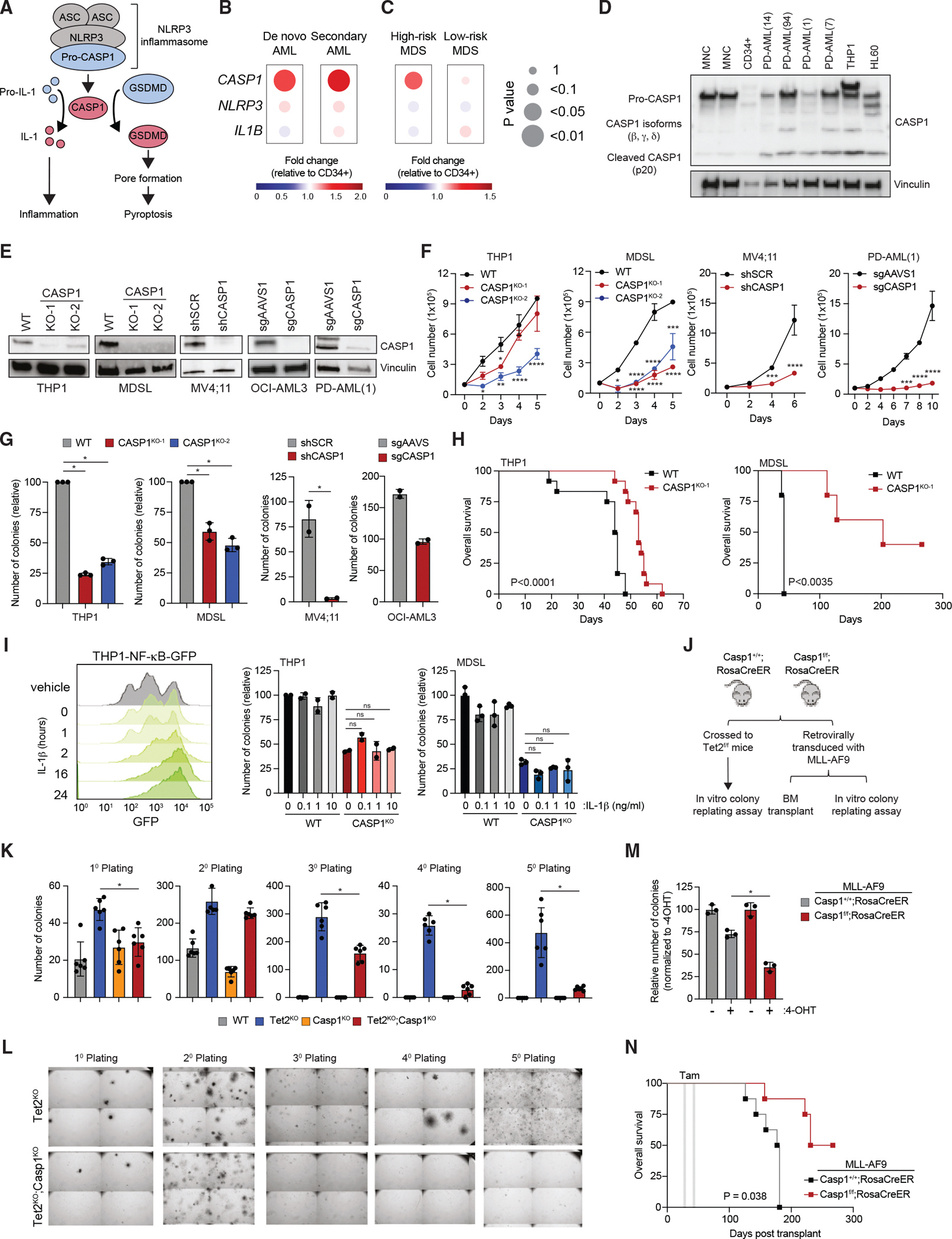
CASP1 but not IL-1β is required for leukemic cells (A) Schematic of Pro-CASP1 activation and function. (B) CASP1 and IL-1β mRNA expression in patients with *de novo* AML (*n* = 476) and secondary AML after prior MDS/MPN (*n* = 25), and healthy human CD34 ^+^ BM cells (*n* = 13) from BEAT-AML. Fold change and *p* values are shown. Refer to Figures S1A and S1B for complete data. (C) CASP1 and IL-1β mRNA expression in high-risk (HR) (*n* = 31) and low-risk (LR) MDS patients (*n* = 24), and healthy human CD34^+^ BM cells (*n* = 17) from Gerstung et al. (GSE58831). Fold change and *p* values are shown. Refer to Figure S1F for complete data. (D) Immunoblot of healthy human mononuclear cells (MNCs) (*n* = 2 donors), healthy human CD34^+^ BM cells, patient-derived (PD) AML cells, and AML cell lines. Error bars represent the standard error of the mean. (E) Immunoblot of the indicated isogenic cell lines and patient-derived (PD) AML cells proficient (WT, sgAAVS1, or non-targeting shRNA [shSCR]) or deficient for CASP1 following CRISPR-Cas9-mediated deletion (KO or sgCASP1) or knockdown (shCASP1). (F) Proliferation of isogenic WT or CASP1-deficient cell lines (*n* = 3 independent replicates). (G) Colony-forming assay of isogenic WT or CASP1-deficient cell lines (*n* = 3 independent replicates). (H) Overall survival of NSGS mice xenografted with isogenic WT or CASP1-deficient THP1 cells (*n* = 12 mice) and MDSL (*n* = 5 mice). Mantel-Cox test was used to determine significance. (I) NF-κB activation in THP1-NF-κB reporter cells (left) and colony-forming assay of isogenic WT or CASP1-deficient THP1 or MDSL (right) cell lines treated with increasing amounts of IL-1β. Colonies were counted at day 7 (THP1) and day 10 (MDSL). (J) Overview of *in vitro* and *in vivo* assays evaluating Casp1-deficient mouse models. (K) Colony-forming assay of mouse cKit+ BM cells isolated from WT;RosaCreER, *Tet2*^f/f^;RosaCreER, *Casp1*^f/f^;RosaCreER, and *Tet2*^f/f^*Casp1*^f/f^;RosaCreER (red) mice treated *in vitro* with 4-OHT. Colonies were counted on day 7 (*n* = 3 independent replicates). Colonies were isolated, and cells were re-plated up to 5 times. (L) Representative images of colonies from (K). (M) Colony-forming assay of lineage negative (Lin-) cells isolated from *Casp1*^f/f^;RosaCreER (red) and *Casp1*^+/+^;RosaCreER (gray) following retroviral expression of MLL-AF9. Transduced AML cells were treated with 4-OHT for 48 h and then plated in methylcellulose. (N) Overall survival of mice engrafted with MLL-AF9 *Casp1*^f/f^;RosaCreER and *Casp1*^+/+^;RosaCreER AML cells. Error bars represent the standard error of the mean. *p < 0.05, **p < 0.01, ***p < 0.001, ****p < 0.0001.

IL-1β processing is controlled by caspase-1 (CASP1), a cysteine-aspartic acid protease that cleaves pro-IL-1β into its active form.^19,20^ CASP1 is expressed as an inactive zymogen (Pro-CASP1) that can be cleaved into its enzymatically active form upon activation of the inflammasome.^20^ The inflammasome is a multiprotein complex that detects cellular stress or pathogenic signals and triggers an inflammatory response.^19^ It typically consists of a sensor protein such as NLRP3 (NOD-, LRR-, and pyrin domain-containing protein 3), an adaptor protein like ASC (apoptosis-associated speck-like protein containing a CARD), and CASP1.^21^ Upon activation, NLRP3 oligomerizes and recruits ASC, activating Pro-CASP1 by inducing its autocleavage into the active subunit.^22^ Activated CASP1 then cleaves pro-IL-1β and pro-IL-18 into their active forms. CASP1 also cleaves gasdermin-D (GSDMD), which induces pyroptosis by forming membrane pores and triggering cell death (Figure 1A). Inflammasome activation is detected in subtypes of MDS and AML.^23–25^ Given the importance of IL-1β and pyroptosis, there has been growing interest in targeting the inflammasome in myeloid malignancies and inflammatory disorders.^26^

One of the key pathways downstream of IL-1β signaling and implicated in MDS/AML involves nuclear factor kappa-light-chain-enhancer of activated B cells (NF-κB).^27,28^ NF-κB activation promotes leukemic cell survival and proliferation; however, sustained or excessive NF-κB signaling can be tumor suppressive by inducing apoptotic or pyroptotic cell death.^29^ Similarly, pre-leukemic cells have acquired an adaptive response to inflammation to subvert the suppressive effects of chronic inflammatory signaling by altering NF-κB activation.^9,13,30–36^ Understanding the regulatory pathways governing inflammatory pathways and responses could expose functional and therapeutic vulnerabilities.

Here, we show that elevated CASP1 expression correlates with poor survival in MDS and AML, implicating CASP1 as a negative prognostic factor. Our data reveal that Pro-CASP1 contributes to leukemogenesis through a non-catalytic mechanism, functioning as a scaffolding hub that regulates NF-κB signaling via its interaction with raptor (RPTOR), a component of mTORC1. Loss of Pro-CASP1 leads to excessive NF-κB activation, leukemic differentiation, and impaired cell function, independent of its enzymatic activity or IL-1β processing. A CRISPR screen identified the CARD domain, rather than the catalytic domain, as essential for leukemic cell function, confirming a scaffolding rather than a proteolytic role of CASP1 in leukemia. To therapeutically exploit this vulnerability, we developed a PROTAC degrader that depletes Pro-CASP1, suppresses colony formation, and drives excessive NF-κB signaling. While IL-1β may contribute to AML progression, our findings indicate that the primary role of CASP1 in leukemia is to scaffold signaling complexes that regulate mTORC1-NF-κB activity.

## RESULTS

### Aberrant CASP1 expression is associated with MDS/AML

The key effectors of the NLRP3 inflammasome implicated in MDS or AML include NLRP3, CASP1, and IL-1β. To examine the gene expression of these inflammasome effectors, we analyzed publicly available data from MDS/AML patients. We found that *CASP1* mRNA expression was higher in *de novo* and secondary AML patient cells relative to healthy CD34^+^ cells (Figures 1B and S1A). Since CASP1 is normally expressed in myeloid cells, we examined CASP1 expression in AML subtypes compared to their respective healthy control cells. *CASP1* mRNA was higher in primitive-like AML (M0 and M1 subtypes) compared to healthy CD34^+^ cells (Figure S1B). Similarly, *CASP1* mRNA was higher in monocytic-like AML (M4 and M5 subtypes) compared to healthy mononuclear cells (MNCs) (Figure S1B). AML patients with elevated CASP1 expression were enriched for mutations in DNMT3A, NPM1, and CBFβ (Figure S1C). In contrast, AML patients with elevated IL-1β expression were not enriched for any of the common mutations (Figure S1D). Across all cancer types, *CASP1* is most highly expressed in AML (Figure S1E), confirming prior studies.^37^ Moreover, *CASP1* mRNA expression was elevated in high-risk MDS CD34^+^ cells as compared to low-risk MDS or age-matched BM CD34^+^ cells (Figures 1C and S1F). In contrast, *NLRP3* and *IL-1B* expression levels were similar between MDS, AML, and healthy controls (Figures 1B, 1C, and S1G). Elevated expression of *CASP1* also correlated with reduced overall survival in both AML and MDS patients (Figure S1H). Notably, full-length zymogen Pro-CASP1 protein was expressed in the majority of human MDS/AML cell lines and patient-derived (PD) AML samples (Figures 1D and S1I). In some cases, we observed both the full-length and activated forms of CASP1, suggesting that inflammasome activation is operational in leukemic cells. In contrast, CD34^+^ cells expressed low levels of Pro-CASP1, while healthy MNCs only expressed the full-length Pro-CASP1 (Figures 1D and S1G). These findings indicate that CASP1 mRNA expression is elevated in MDS/AML and correlates with increased full-length Pro-CASP1 protein expression.

### CASP1, but not IL-1β, is required for MDS/AML leukemic cells

To investigate the role of Pro-CASP1 in leukemic cells, we generated CASP1-deficient (CASP1^KO^) human isogenic cell lines (MDSL, THP1, MV4;11, and OCI-AML3) and PD AML cells (Figures 1E and S2A). As expected, CASP1^KO^ leukemic cells were defective in pyroptosis, including GSDMD cleavage, IL-1β protein expression, and lactate dehydrogenase release (Figures S2B–S2D). Despite impaired pyroptotic cell death, CASP1^KO^ leukemic cells showed a reduction in proliferation in liquid culture (Figure 1F) and colony-forming potential in methylcellulose (Figure 1G). Wright-Giemsa staining of THP1 and MDSL CASP1^KO^ cells showed increased cytoplasmic size and more differentiated cell morphology compared to isogenic wild-type (WT) cells (Figure S2E). This was accompanied by increased expression of myeloid activation markers (CD38 and CD14) and decreased expression of the immature hematopoietic cell marker CD34^38–42^ (Figure S2F). Moreover, CASP1^KO^ leukemic cells exhibited apoptosis and moderate accumulation in S-phase (Figure S2G). However, this phenotype is not indicative of cellular senescence (Figure S2H). This suggests that loss of CASP1 in AML results in premature differentiation, replicative stress, and apoptosis. Re-expression of full-length CASP1 restored leukemic potential in CASP1^KO^ MDSL and THP1 cells, rescuing colony-forming capacity in methylcellulose and morphologic differentiation, indicating that these leukemic cell defects are directly related to CASP1 expression (Figures S2I–S2K). To determine whether CASP1-deficient leukemic cells are defective in their potential to establish leukemia, we xenografted isogenic WT and CASP1^KO^ THP1 and MDSL cells into immunocompromised mice. Deletion of CASP1 in THP1 and MDSL cells significantly reduced leukemic cell burden and extended the overall survival of the recipient mice (Figure 1H). These findings indicate that CASP1 is required for maintaining the undifferentiated and clonogenic potential of human leukemic cells.

Since IL-1β has been shown to support AML cell expansion and survival, we wanted to determine whether the CASP1^KO^ leukemic cells are impaired due to reduced autocrine IL-1β signaling.^11–13^ First, we confirmed that exogenous IL-1β can activate NF-κB in THP1 cells using cells expressing a destabilized GFP driven by an NF-κB response element^43,44^ (Figures 1I and S2L). At these concentrations, we found that the addition of IL-1β to the media was unable to restore the colony-forming defect of CASP1^KO^ THP1 and MDSL cells (Figure 1I). These findings suggest that the impaired function of CASP1-deficient AML cells is not due to reduced IL-1β expression.

MDS and AML arise from mutant HSPCs.^3^ Therefore, we sought to examine the role of CASP1 in pre-leukemic and AML development using HSPCs derived from conditional Casp1 loss-of-function mouse models (Figure 1J). To determine whether Casp1 is required for Tet2-deficient HSPC clonal expansion, we generated *Casp1*^f/f^;*Tet2*^f/f^ mice with an inducible RosaCreER recombinase strain. Upon *in vivo* tamoxifen or *in vitro* 4-hydroxytamoxifen (4-OHT) treatment, Cre induces the deletion of *Casp1* and *Tet2* (Figures S3A and S3B). As expected, deletion of Tet2 (*Tet2*^−/−^) alone resulted in serial colony formation of cKit+ HSPCs of 5 platings in methylcellulose compared to WT (*Tet2*^+/+^;*Casp1*^+/+^) and *Casp1*^−/−^ HSPCs (Figures 1K and 1L). However, deletion of Casp1 in Tet2-deficient HSPCs (*Tet2*^−/−^;*Casp1*^−/−^) significantly suppressed the expansion and self-renewal capacity observed upon deletion of Tet2 alone (Figures 1K and 1L). To determine whether CASP1 is required for overt AML, we retrovirally expressed the oncogenic driver mutation MLL-AF9 in cKit+ BM cells isolated from *Casp1*^f/f^;RosaCreER mice (MLL-AF9+*Casp1*^f/f^) or control Casp1^+/+^; RosaCreER mice (MLL-AF9+*Casp1*^+/+^). *In vitro* 4-OHT treatment resulted in *Casp1* excision and loss of CASP1 expression in MLL-AF9+*Casp1*^f/f^ AML cells (Figure S3C). MLL-AF9+*Casp1*^−/−^cells exhibited a loss of leukemic progenitor cell function in methylcellulose colony assays (Figure 1M). We next determined the requirement of *Casp1* on AML development *in vivo*. MLL-AF9+*Casp1*^f/f^ or MLL-AF9+*Casp1*^+/+^ cells were transplanted along with helper BM cells into lethally irradiated mice. After 4 weeks of engraftment, recipient mice were administered tamoxifen intraperitoneally to induce deletion of *Casp1*. Mice transplanted with MLL-AF9+*Casp1*^+/+^ cells exhibited hallmarks of leukemia including circulating myeloid blasts and overall morbidity (Figures 1N and S3D). In contrast, upon deletion of Casp1, mice engrafted with MLL-AF9+*Casp1*^−/−^ had reduced circulating AML cells and prolonged overall survival as compared to mice engrafted with MLL-AF9+*Casp1*^+/+^ cells (Figures 1N and S3D). In contrast, deletion of Casp1 in healthy BM cells did not impact blood counts in recipient mice (Figure S3E). These findings suggest that CASP1 is necessary for pre-leukemic and transformed leukemic cells but dispensable for normal hematopoiesis.

### The scaffolding function of CASP1 is required for leukemic cells

**Figure 2.**
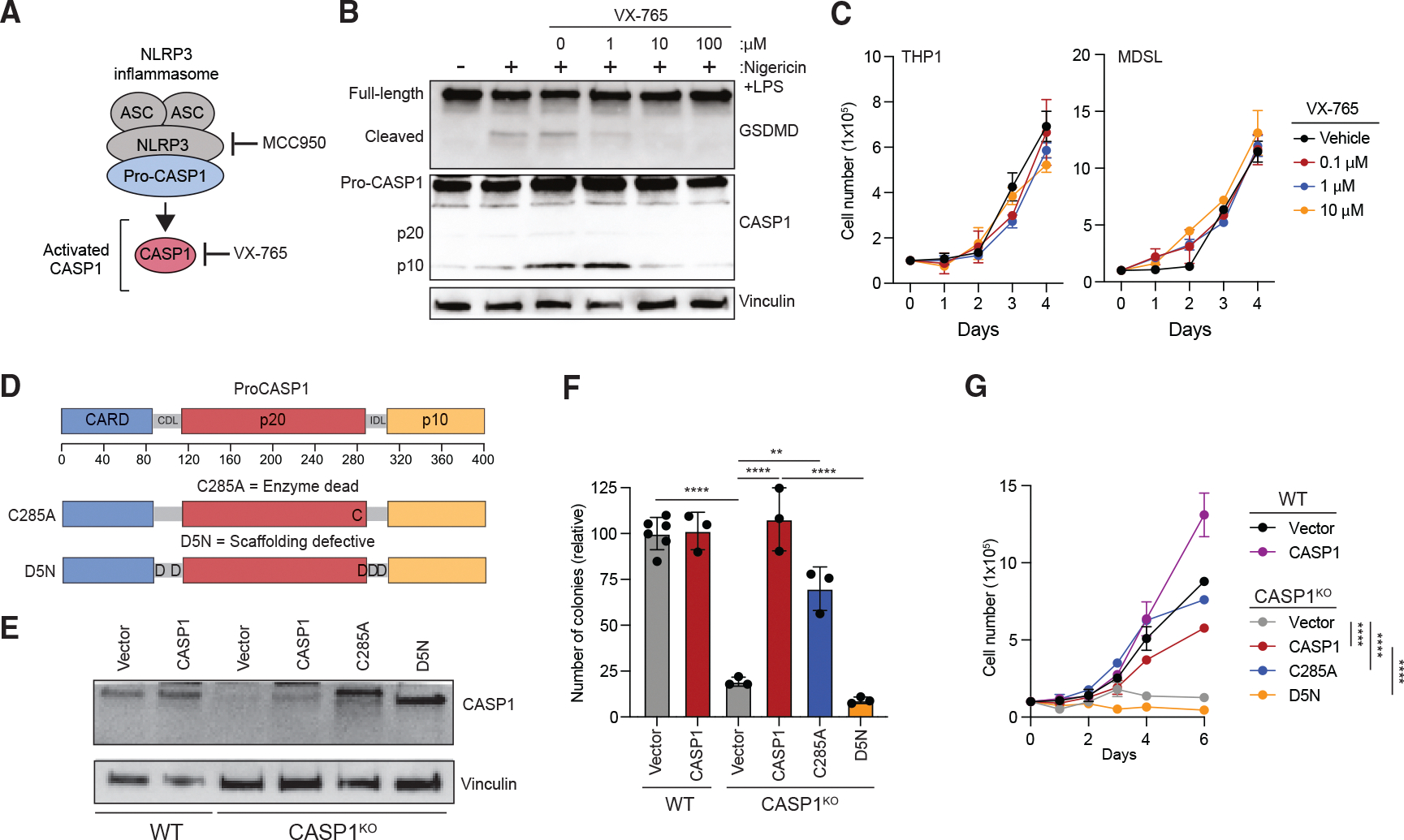
The scaffolding function of CASP1 is required for leukemic cells (A) Schematic of CASP1 and NLRP3 inhibitors VX-765 and MCC950, respectively. (B) Immunoblot of THP1 cells treated with LPS (24 h of 10 μg/mL) and nigericin (2 h of 20 μM) to activate the inflammasome and increasing concentrations of VX-765. (C) Proliferation of the indicated cell lines treated with increasing concentrations of VX-765 (*n* = 3 independent biological replicates). (D) Overview of WT, enzymatically dead (C285A), and scaffolding dead (D5N) CASP1. (E) Immunoblot of WT and CASP1-deficient THP1 cells expressing empty vector, WT CASP1, CASP1-C285A, or CASP1-D5N. (F) Colony-forming assay of WT and CASP1-deficient THP1 cells expressing empty vector, WT CASP1, CASP1-C285A, or CASP1-D5N (*n* = 6 independent biological replicates). (G) Proliferation of WT and CASP1-deficient THP1 cells expressing empty vector, WT CASP1, CASP1-C285A, or CASP1-D5N (*n* = 3 independent biological replicates). Error bars represent the standard error of the mean. *p < 0.05, **p < 0.01, ***p < 0.001, ****p < 0.0001.

Pro-CASP1 becomes an activated cystine-protease upon autocleavage of its N-terminal CARD domain, resulting in the shorter activated form (p20). Although IL-1β appears to be dispensable in CASP1^KO^ AML cells (Figure 1I), we wondered whether the proteolytic function of activated CASP1 might still be necessary for other mechanisms. To test this, we utilized a CASP1-specific cystine protease inhibitor (VX-765) that blocks CASP1’s ability to cleave its substrates (Figure 2A).^45^ VX-765 effectively prevented CASP1 and GSDMD cleavage in AML cells stimulated with lipopolysaccharide (LPS) and nigericin to activate the inflammasome (Figure 2B). However, treating MDS/AML cells with VX-765 had no effect on proliferation and colony formation (Figures 2C and S4A). Similarly, the inflammasome inhibitor MCC950 did not affect leukemic cell growth (Figure S4B).^46^ To confirm the non-catalytic role of CASP1 in leukemic cells, we generated isogenic THP1 cells expressing either WT CASP1, a scaffolding-defective mutant (D5N, with five asparagine substitutions for aspartic acid) that retains enzymatic activity, or an enzymatically inactive mutant (C285A) (Figures 2D and 2E).^20,47,48^ As expected, the scaffolding-defective and enzymatically inactive CASP1 mutants were unable to activate the inflammasome and induce pyroptosis (Figures S4C and S4D). However, expression of both WT CASP1 and the enzymatically inactive mutant (C285A) rescued the colony-forming and proliferation defects in CASP1^KO^ AML cells (Figures 2F and 2G). In contrast, the scaffolding-defective mutant (D5N) failed to restore these functional defects (Figures 2F and 2G). Collectively, these findings indicate that the scaffolding role of CASP1, rather than its catalytic function, is essential for leukemic cell function.

**Figure 3.**
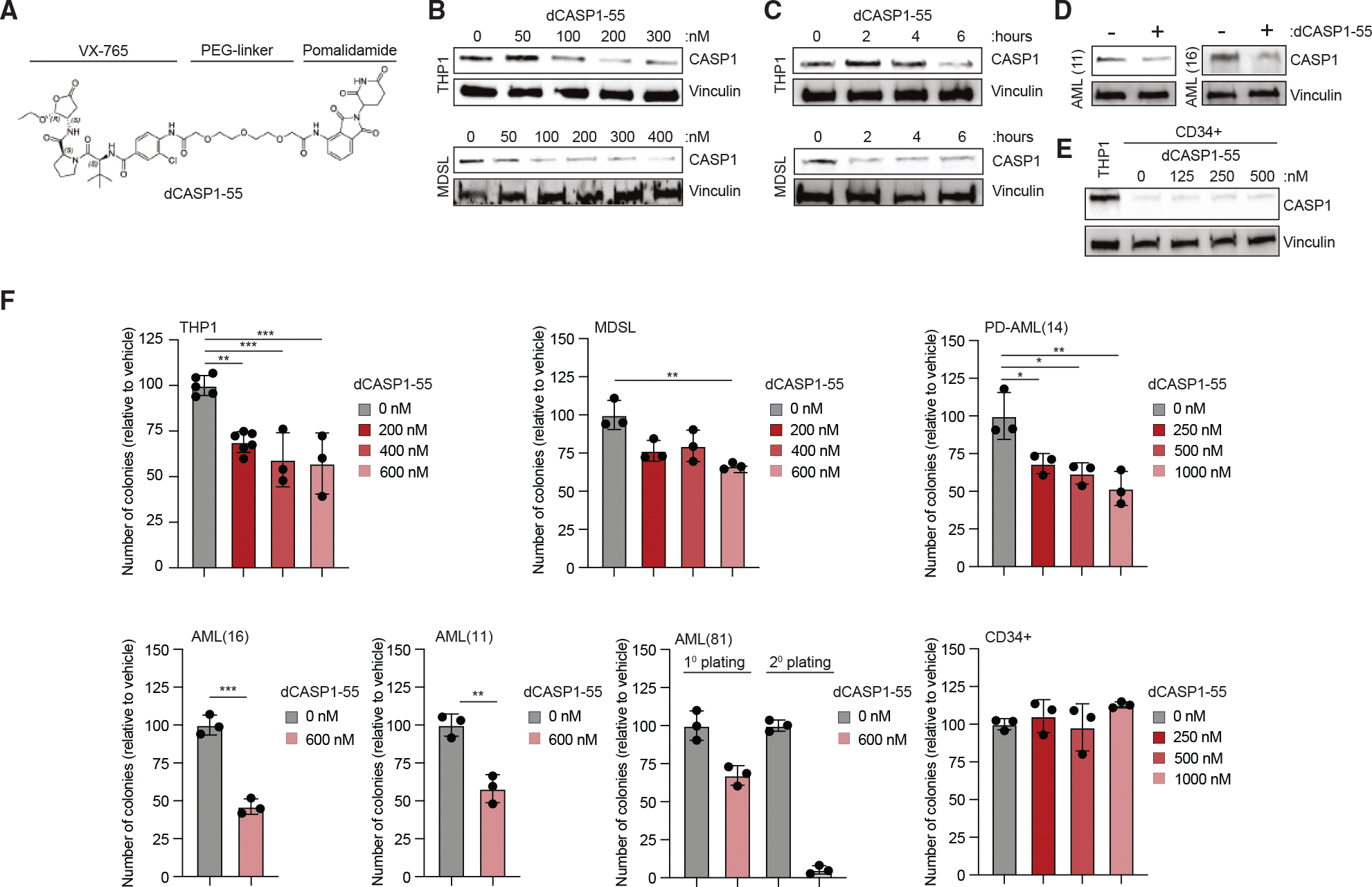
CASP1 PROTAC suppresses leukemic cells (A) Structure of CASP1 PROTAC (dCASP1–55). (B) Immunoblot of the indicated cell lines treated with dCASP1–55 for 6 h. (C) Immunoblot of the indicated cell lines treated with dCASP1–55 for the indicated times. (D) Immunoblot of primary AML patient samples treated with dCASP1–55 for 6 h. (E) Immunoblot of healthy CD34 ^+^ cells treated with dCASP1–55 for 6 h. THP1 cells were included as a positive control for CASP1 expression. (F) Colony-forming assay of the indicated cell lines, patient-derived (PD) AML samples, and primary AML samples treated with increasing concentrations of dCASP1–55 (*n* = 3 independent biological replicates). Error bars represent the standard error of the mean. *p < 0.05, **p < 0.01, ***p < 0.001, ****p < 0.0001.

To further investigate the role of CASP1 as a scaffolding protein and therapeutic target, we aimed to selectively degrade CASP1 rather than inhibiting its enzymatic activity. Since VX-765 is a potent and selective CASP1 inhibitor, we modified it into a proteolysis-targeting chimera (PROTAC) to mediate the selective degradation of CASP1 through the ubiquitin-proteasome system.^49,50^ This was achieved by conjugating VX-765 to the cereblon recruiter pomalidomide using a 3-mer PEG-linker, generating dCASP1–55 (Figure 3A). dCASP1–55 degraded CASP1 protein in a dose- and time-dependent manner in THP1 and MDSL (Figures 3B and 3C) and in PD AML samples (Figure 3D). Treatment of cell lines and PD AML samples with dCASP1–55 also resulted in suppression of leukemic colony formation in methylcellulose (Figure 3F). dCASP1–55 also suppressed both primary and secondary AML colony formation, indicating that CASP1 deletion can target the serially replating AML cell population, an indirect measure of leukemic stem cells (Figure 3F). Importantly, the colony-forming potential of human CD34^+^ BM cells, which express very low levels of CASP1 (Figure 3E), was unaffected by dCASP1–55 (Figure 3F). Similar to CASP1^KO^ AML cells, dCASP1–55 treatment of THP1 cells induced apoptosis and moderate S-phase arrest (Figure S4E). To confirm that dCASP1–55 specifically induces cereblon-mediated degradation of CASP1, WT THP1 cells were transduced with either an empty vector (sgControl) or cereblon single-guide RNA (sgRNA) (sgCRBN) lentiviral vectors expressing Cas9 (Figure S4F). Control THP1-sgAAVS1 cells showed dose-dependent degradation of Pro-CASP1, while no degradation was observed in THP1-sgCRBN cells, confirming that dCASP1–55 selectively degrades CASP1 in a cereblon-dependent manner.^51^ Moreover, dCASP1–55 did not induce degradation of CASP4 (Figure S4G). These results reveal the importance of full-length CASP1 as an essential scaffolding protein for leukemic cell function and establish CASP1 PROTACs as promising therapeutic strategies for targeting CASP1 in MDS/AML.

### CASP1-deficient leukemic cells exhibit excessive NF-κB activation

**Figure 4.**
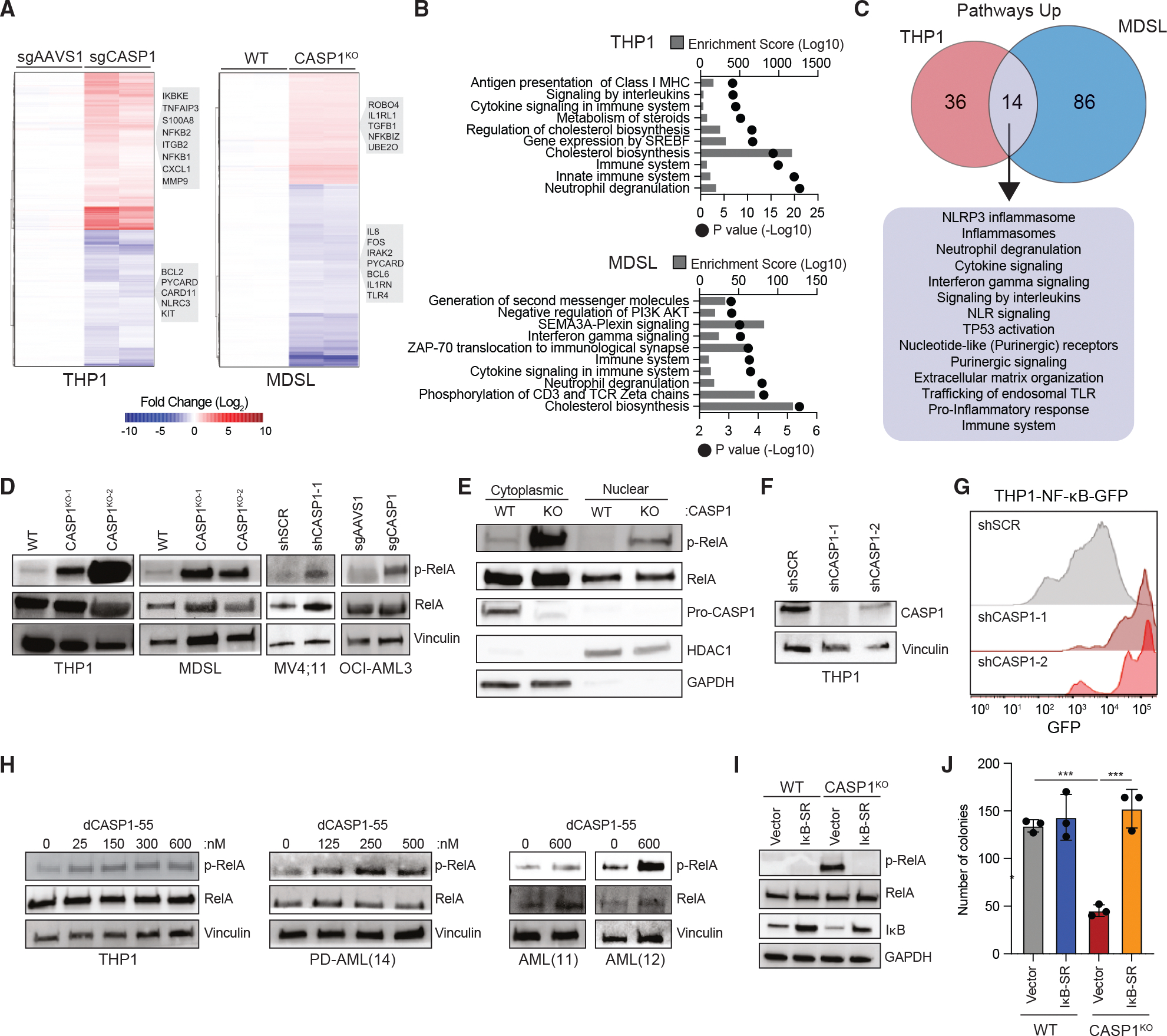
CASP1-deficient leukemic cells exhibit excessive NF-κB activation (A) Differentially expressed genes in the indicated WT and CASP1-deficient THP1 and MDSL cell lines (2-fold, *p* < 0.05, *n* = 2 biological replicates per group). (B) Pathway analysis of differentially expressed genes in WT and CASP1-deficient THP1 and MDSL cell lines. Shown is the top pathways based on normalized enrichment score and corresponding *p* values (−log10). (C) Overlapping Hallmark enriched pathways using the upregulated genes in WT and CASP1-deficient THP1 and MDSL cell lines. (D) Immunoblot of WT and CASP1-deficient cells. (E) Immunoblot of the cytoplasmic and nuclear fractions of WT and CASP1-deficient THP1 cells. (F) Immunoblot of THP1 cells expressing control non-targeting shRNA (shSCR) or two independent shRNAs targeting CASP1. (G) NF-κB reporter activity in THP1 cells expressing pSIRV-NF-κB-eGFP vector was measured by flow cytometry. (H) Immunoblot of the indicated samples treated with dCASP1–55. (I) Immunoblot of WT and CASP1-deficient THP1 cells expressing the IκBα super repressor mutant (IκB-SR). (J) Colony-forming assay of WT and CASP1-deficient THP1 cells expressing IκB-SR. Colonies were counted on day 10 (*n* = 3 independent biological replicates). Error bars represent the standard error of the mean. *p < 0.05, **p < 0.01, ***p < 0.001, ****p < 0.0001.

To understand the molecular basis for CASP1 dependency in leukemic cells, we performed a transcriptomic analysis on isogenic WT and CASP1^KO^ THP1 and MDSL cells (Figure S5A). Deletion of CASP1 resulted in significantly differentially expressed genes as compared to WT isogenic cells (Figure 4A). 605 genes exhibited an increase in expression, while 523 genes showed a decrease in expression in CASP1^KO^ THP1 as compared to WT cells (Table S1). 593 genes exhibited an increase in expression, while 962 genes showed a decrease in expression in CASP1^KO^ MDSL as compared to WT cells (Table S2). Pathway analysis of all differentially expressed genes in CASP1^KO^ THP1 and MDSL cells revealed significant enrichment of pathways related to the immune system, cytokine signaling, and myeloid differentiation (Figure 4B). Among the overexpressed genes, pathway analysis specifically showed enrichment of multiple pathways associated with innate immune signaling, NLPR3 inflammasomes, neutrophil degranulation, and interferon signaling in both CASP1^KO^ THP1 and MDSL cells (Figure 4C). Previous studies have linked CASP1 to inflammasome activation, resulting in the release of IL-1β and IL-18, as well as the induction of pyroptosis. However, the widespread activation of inflammatory-related pathways observed following CASP1 loss was unexpected, suggesting a more complex role for CASP1 in regulating immune signaling.

Since CASP1 deletion led to the activation of immune pathways, we wanted to determine whether this was driven by NF-κB. To investigate this, we examined the phosphorylated form of RelA, the main active subunit of the NF-κB dimer. CASP1-deficient AML cell lines showed significantly higher levels of phosphorylated RelA compared to their isogenic WT counterparts (Figures 4D and S5A). Expression of WT CASP1 into CASP1^KO^ THP1 cells restored NF-κB activation, suggesting that CASP1 is directly responsible for regulating NF-κB in AML cells (Figure S5B). Furthermore, phosphorylated RelA was predominantly localized in the nucleus in CASP1^KO^ cells (Figure 4E), at levels similar to those observed following acute stimulation with IL-1β (Figure S5C), indicating its active state. Knockdown of CASP1 also led to NF-κB-mediated activation of target genes, as demonstrated by an NF-κB reporter assay in THP1 cells (Figures 4F and 4G). Moreover, dCASP1–55 treatment of THP1 cells and patient AML samples resulted in increased phosphorylated RelA (Figure 4H). These findings suggest that deletion of CASP1 results in excessive NF-κB activation in AML cells.

To determine whether excessive NF-κB activation is responsible for the leukemic cell defects observed after CASP1 deletion, we aimed to restore normal NF-κB levels. We did this by expressing a dominant-negative IκBα super repressor (IκB-SR), which is resistant to proteasomal degradation and inhibits RelA.^52^ Expression of IκB-SR in CASP1^KO^ cells successfully reduced NF-κB activation back to baseline levels, similar to what we observed in WT cells (Figure 4I). Additionally, suppressing NF-κB activation with IκB-SR restored leukemic colony formation in CASP1^KO^ cells (Figure 4J). These findings suggest that CASP1-deficient AML cells exhibit excessive NF-κB activation, independent of its catalytic function, which impairs leukemic cells.

### Defining the critical scaffolding domain of CASP1 in leukemia

**Figure 5.**
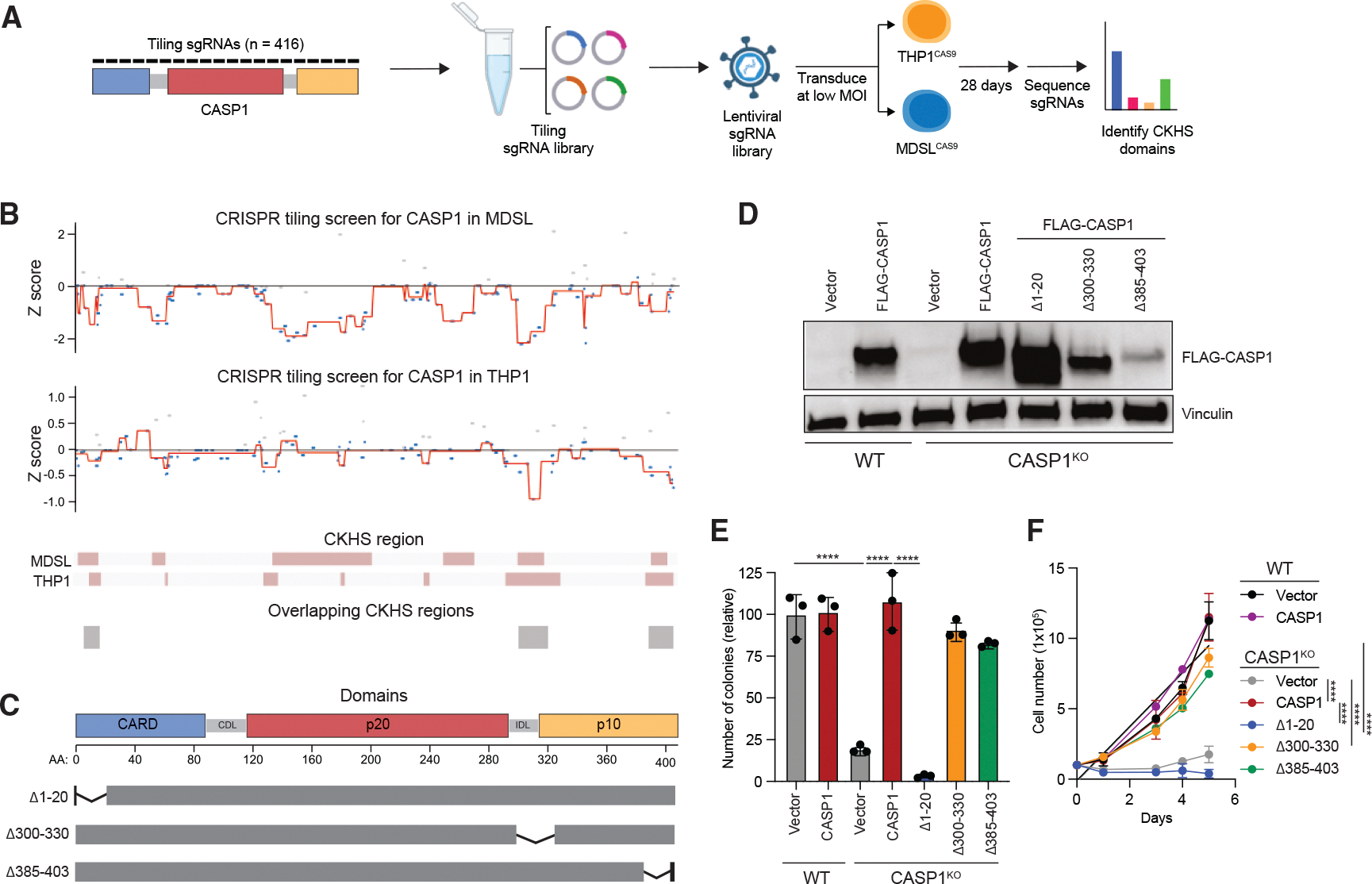
CASP1 scaffolding function is required for leukemic cells (A) Overview of Tiling array CRISPR dropout screen to identify critical domains with CASP1. (B) sgRNA dependency mapping of *Z* scores to the transcript of the Pro-CASP1 in MDSL (top) and THP1 cells using Pro-Tiler. ^54^ CRISPR knockout high sensitivity (CKHS) regions were identified based on elevated *Z* score and mapped to the transcript. Overlapping CKHS regions between MDSL and THP1 cells were identified (gray bars). CKHS was determined as a guide having a *Z* score below −0.25 for THP1 and below −1.0 for MDSL. (C) Overview of prioritized and overlapping CKHS CASP1 regions. (D) Immunoblot of WT and CASP1-deficient THP1 cells expressing the indicated CASP1 mutants. (E) Colony-forming assay of WT and CASP1-deficient THP1 cells expressing the indicated CASP1 mutants (*n* = 3 independent biological replicates). (F) Proliferation of WT and CASP1-deficient THP1 cells expressing the indicated CASP1 mutants (*n* = 3 independent biological replicates). Error bars represent the standard error of the mean. *p < 0.05, **p < 0.01, ***p < 0.001, ****p < 0.0001.

To identify which scaffolding domains of CASP1 are essential for leukemic cells, we performed an *in vitro* tiled CRISPR dropout screen of Pro-CASP1 utilizing the Pro-Tiler pipeline.^53,54^ This approach delineates CRISPR knockout high sensitivity (CKHS) domains (Figure 5A). We generated a library of 416 sgRNAs: 284 sgRNAs targeting every PAM sequence within the CASP1 transcript, 10 non-targeting sgRNAs as controls, and 122 essential control sgRNAs. These were cloned into puromycin-selective lentiviral vectors and transduced into THP1 and MDSL cells expressing Cas9. We achieved a coverage of 4,800× (2 million cells per 416 sgRNAs) for each replicate collected at day 28. By mapping high-dropout CKHS regions to the functional domains of CASP1, we identified that the N-terminal CARD domain, the inter-domain linker (IDL) domain, and C-terminal p10 domain, but not regions near the protease domain (C285), are essential for the growth of both THP1 and MDSL cells (Figure 5B).^48,54^ Since both THP1 and MDSL cells shared CKHS regions, we next generated isogenic CASP1^KO^ cell lines expressing WT CASP1 or mutants with deletions of the CARD (Δ1–20), IDL (Δ300–330), and p10 (Δ386–403) domains (Figures 5C and 5D). Expression of WT CASP1, as well as the Δ300–330 and Δ386–403 mutants, restored colony formation and growth in CASP1^KO^ THP1 cells (Figures 5E and 5F). In contrast, the Δ1–20 CASP1 mutant failed to rescue the growth defects observed in CASP1^KO^ AML cells (Figures 5E and 5F), confirming the essential role of the CARD domain. The CARD domain (aa 1–90) forms a structural interface mediating key electrostatic and hydrophobic contacts, yet the specific role of its first 20 amino acids remains uncharacterized. Interestingly, the reduced function of leukemic cells expressing Δ1–20 CASP1 appears to be largely independent of canonical inflammasome activation and pyroptosis, as the Δ1–20 CASP1 AML cells still exhibit GSDMD cleavage and pyroptotic activity (Figures S5D and S5E). Together, these findings demonstrate that the proteolytic function of CASP1 is dispensable, while the N-terminal CARD domain, a key scaffolding region, is crucial for maintaining AML cells.

### CASP1 regulates NF-κB through mTORC1 in leukemic cells

**Figure 6.**
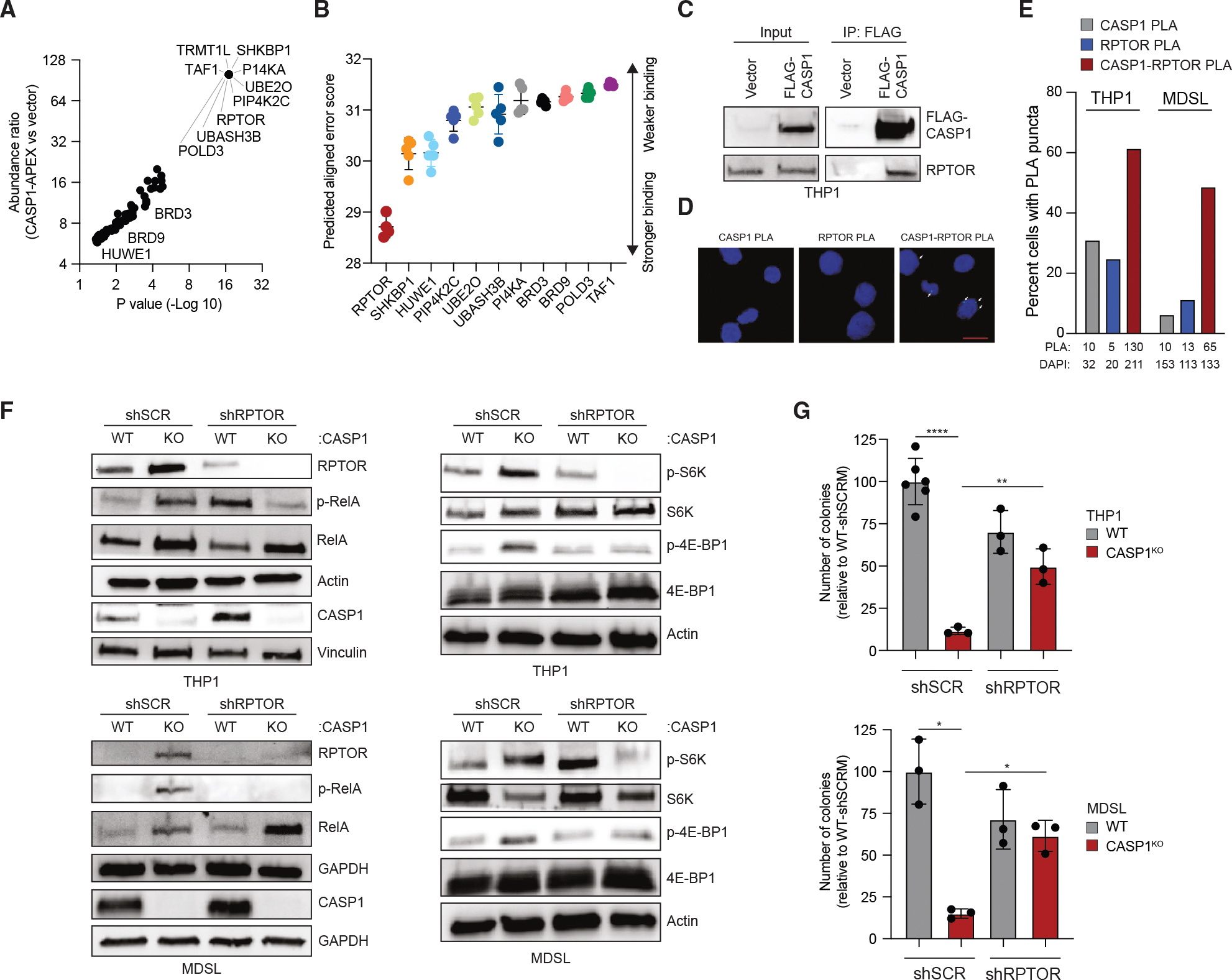
CASP1 regulates NF-κB through mTORC1 (A) Summary of CASP1 proximity interactome analysis in THP1 cells using APEX. Shown are *p* values (−log10) relative to the abundance ratios of CASP1-APEX versus empty vector. (B) Predicted aligned error (PAE), a measure of AlphaFold3’s confidence in the relative positioning of two residues within a predicted protein, was scored between CASP1 scaffolding domains in proximity to the full length of the top proximal protein candidates identified in (G). (C) Co-immunoprecipitation in THP1 cells expressing FLAG-CASP1. (D) Representative confocal images of proximity ligation assay (PLA) between CASP1 and RPTOR in THP1 cells. As negative controls, CASP1 or RPTOR PLA alone was performed. (E) Summary of PLA between CASP1 and RPTOR in THP1 and MDSL cells. The percent of DAPI cells with PLA-positive puncta was calculated. (F) Immunoblots of WT and CASP1-deficient THP1 and MDSL cells. (G) Colony-forming assay of THP1 and MDSL cells expressing control non-targeting shRNA (shSCR) or shRNAs targeting RPTOR (shRPTOR). Colonies were counted on day 10 (*n* = 6 and 3 independent biological replicates, respectively). Error bars represent the standard error of the mean. *p < 0.05, **p < 0.01, ***p < 0.001, ****p < 0.0001.

To understand how the scaffolding function of CASP1 regulates NF-κB in leukemic cells, we mapped the network of proteins that associate with CASP1. We used a proximity-based proteomic labeling approach by tagging the N-terminal CARD domain of CASP1 with APEX2, a biotin-labeling enzyme, and expressing it into CASP1^KO^ THP1 cells.^48^ After biotin labeling, we performed mass spectrometry to identify CASP1 proximal proteins.^55^ In total, we identified 102 proteins (Figure 6A; Table S3). To identify which of the proximal proteins are most likely to directly bind CASP1, we used a computational pipeline based on predicted aligned error (PAE), which measures AlphaFold’s confidence in the spatial relationship between residues of two proteins.^56,57^ We applied this method to map the proximity of the top candidate proteins identified in the proximity screen to the CASP1 CKHS scaffolding domains (Figure 6B). Our analysis revealed that RPTOR, a key co-factor of mTORC1 and PI3K activity, had the lowest mean PAE score, indicating the closest proximity and a high likelihood of direct binding to CASP1 (Figure 6B). To validate the binding between CASP1 and RPTOR, we first performed a co-immunoprecipitation assay using THP1 and MDSL cells expressing an FLAG epitope-tagged CASP1, confirming that CASP1 and RPTOR can interact in leukemic cells (Figures 6C and S5F). To determine whether endogenous CASP1 and RPTOR interact in leukemic cells, we performed a proximity ligation assay that allows *in situ* detection of protein-protein interactions^58^ and found that CASP1 does reside in proximity with RPTOR in >50% of THP1 and MDSL cells (Figures 6D, 6E, and S5G). To extend the proximity-based studies, size-exclusion chromatography was performed on isogenic WT and CASP1^KO^ THP1 cell extracts and analyzed by HPLC column chromatography. Consistent with our observations that CASP1 and RPTOR interact and regulate NF-κB activation, CASP1, RPTOR, IKKα, and IKKβ all eluted in the higher molecular weight fractions (fractions 17–22) (Figure S5H). Moreover, deletion of CASP1 resulted in an increased proportion of RPTOR in the lower molecular weight fractions (fractions 22–25) (Figures S5H and S5I). We also observed a moderate change in the elution distribution of IKKα in CASP1^KO^ THP1 cells (fractions 17–20) (Figures S5H and S5I). This suggests that CASP1 impacts RPTOR-mTORC1 and IKK-NF-κB signaling complexes. These findings were further supported by gene set enrichment analysis on CASP1^KO^ THP1 and MDSL cells, which revealed that mTORC1 and PI3K pathways were enriched when CASP1 was deleted (Figure S5J), suggesting a potential shared dependency between CASP1 and mTORC1 activity.

Lastly, we assessed whether silencing RPTOR could rescue excessive NF-κB activation and the leukemic colony-forming defect of CASP1^KO^ leukemic cells. Expression of short hairpin RNAs (shRNAs) targeting RPTOR, as expected, reduced mTORC1 signaling as indicated by lower levels of phosphorylated S6K and 4E-BP1 (Figures 6F and S5K). Importantly, knockdown of RPTOR rescued excessive NF-κB activation in CASP1^KO^ leukemic cells as observed by reduced phosphorylated RelA (Figure 6F). However, knockdown of RPTOR did not have an impact on inflammasome activation (Figures S5L and S5M). Lastly, knockdown of RPTOR restored leukemic colony formation in CASP1^KO^ cells (Figure 6G). These findings demonstrate that the scaffolding function of CASP1 is crucial for preventing excessive NF-κB signaling through its interaction with the RPTOR-mTORC1 complex in AML.

## DISCUSSION

In this study, we identified a critical function of CASP1 in myeloid malignancies beyond its canonical role in IL-1β processing or pyroptosis induction. Our findings demonstrate that CASP1 acts as a scaffolding hub in leukemia, directly interacting with RPTOR, an essential co-factor of mTORC1, to regulate NF-κB signaling. Loss of CASP1 induces cell-intrinsic excessive NF-κB activation, leading to leukemic cell differentiation at the expense of self-renewal and apoptosis. Importantly, the requirement for CASP1 in leukemic cells is independent of IL-1β. We identified that the scaffolding role of CASP1, rather than its catalytic domain, is essential for leukemic cell function. We further developed a PROTAC-based degrader that specifically degrades CASP1, which induced excessive NF-κB activation and suppression of leukemic cells. Our data suggest that CASP1 primarily functions as a scaffolding protein to regulate mTORC1-NF-κB activity rather than acting as an inflammatory caspase in leukemic cells.

In the context of hematologic malignancies, the role of inflammasomes remains complex.^24,25,59^ For example, NLRP3 has been implicated as a driver of MDS and AML, especially in patients with KRAS mutations.^24^ In a mouse model expressing Kras^G12D^, NLRP3 deficiency reversed myeloproliferation and cytopenia.^60^ However, targeting NLRP3 alone is challenging due to its role as a scaffolding protein and its structural complexity. Additionally, the redundancy among NLR inflammasome family members complicates the development of targeted therapies. IL-1β is a key mediator of inflammation that can regulate hematopoiesis. However, IL-1β signaling can also drive AML progression.^11,14^ Studies have shown that IL-1R knockdown reduces AML colony-forming capacity while restoring IL-1β increases proliferation.^11^ Additionally, increased expression of IL-1 receptor accessory protein correlates with poor prognosis in AML.^14^ However, the direct role of IL-1β in MDS/AML remains controversial.^12,13,31^ IL-1β is not significantly upregulated in AML patient plasma, and targeting IL-1β with monoclonal antibodies has failed in clinical trials for MDS.^16–18^ These findings suggest that while IL-1β may modify the pathogenesis of MDS and AML, it alone may not be the key driver of disease. Compared to IL-1β and NLRP3, the role of CASP1 in MDS/AML has been less clear. Traditionally, CASP1 is known for its role in activating IL-1β and GSDMD, leading to pyroptosis.^19,20,48^ However, in normal hematopoiesis, pharmacological inhibition of CASP1 can induce a myeloid differentiation bias, suggesting broader functional implications.^61^ Our study confirms that CASP1 expression is significantly upregulated in both MDS and AML, correlating with worse survival outcomes. Moreover, our findings challenge the traditional view that CASP1 activation is purely a terminal event leading to cell death. Instead, we demonstrate that CASP1 functions as a scaffolding hub, particularly through interactions with RPTOR, to regulate NF-κB and mTORC1 signaling. This scaffolding function is essential for maintaining leukemic potential, while the production of IL-1β appears secondary.

While often considered pro-tumorigenic, excessive NF-κB activation can be detrimental to cancer cells.^27,29^ Previous studies have shown that sustained elevated canonical NF-κB signaling can trigger a cell-intrinsic immune response leading to cell death.^29,62^ For example, in AML, deletion of the innate immune signaling protein IRF2B2 induced a cell-intrinsic innate immune response, which resulted in cell death.^29^ Our results similarly suggest that leukemic cells require precise tuning of NF-κB signaling to maintain viability. Consistent with these results, previous studies have shown that leukemic cells can adapt to restraining the detrimental effects of elevated canonical NF-κB by shifting to non-canonical NF-κB activation, which allows the cells to persist in an inflammatory environment.^36,63^ Deletion of CASP1 disrupts this balance, leading to uncontrolled NF-κB activation, which ultimately suppresses the leukemic potential of MDS/AML cells. Our study reveals that CASP1, through its interaction with RPTOR, acts as an organizational center for mTORC1, modulating NF-κB signaling in leukemic cells.^64,65^

Herein, we demonstrate that the loss of CASP1 leads to the constitutive activation of mTORC1 and NF-κB signaling in leukemic cells, resulting in a loss of leukemic potential. We propose targeting CASP1 as a therapeutic approach to sensitize AML cells to standard-of-care therapies. By eliminating the scaffolding function of CASP1, particularly through the CASP1 PROTAC, we can disrupt the mTORC1-NF-κB axis, rendering leukemic cells more vulnerable to differentiation and cell death.^66^ Thus, our findings position CASP1 as a promising therapeutic target in leukemia.

### Limitations of the study

While our study provides compelling mechanistic and functional evidence that CASP1 operates as a non-catalytic scaffolding hub regulating mTORC1-NF-κB signaling in leukemia, several limitations should be noted. First, the precise molecular details of how CASP1 coordinates these complexes remain incompletely defined. Second, most functional studies were performed in leukemic cell lines and xenograft models, which may not fully recapitulate the heterogeneity and microenvironmental influences present in patients. Third, although we demonstrate proof-of-principal activity of the CASP1 PROTAC, its pharmacologic properties, specificity *in vivo*, and potential off-target effects require further optimization. Finally, while our data suggest that the effects of CASP1 loss are primarily cell intrinsic, we cannot exclude the possibility that cytokine-mediated immune activation contributes to the *in vivo* phenotype.

## RESOURCE AVAILABILITY

### Lead contact

Further information and requests for resources and reagents should be directed to and will be fulfilled by the lead contact, Daniel T. Starczynowski (daniel.starczynowski@cchmc.org).

### Materials availability

Plasmids used in this study were either purchased from or are publicly available through several online repositories, including Addgene, or were gifted by colleagues. Sources and identifiers of these constructs are provided in the key resources table. All unique resources generated in this study are available from the lead contact with a completed materials transfer agreement.

### Data and code availability

- The RNA sequencing data have been deposited at NCBI’s GEO repository with accession numbers (GSE294653 and GSE294654) and are publicly available as of the date of publication. RNA sequencing data of AML patients ^67^ were downloaded from the GDC Data Portal (https://portal.gdc.cancer.gov/) and the BEAT AML (Vizome, http://www.vizome.org/aml/).^68^ Published microarray data of patients with MDS^69^ and respective age-matched controls were downloaded from GSE58831. Source data are provided with this paper (Data S1, S2, and S3).
- This paper does not report original code.
- Any additional information required to reanalyze the data reported in this paper is available from the lead contact upon request.

## STAR★METHODS

### EXPERIMENTAL MODELS AND STUDY PARTICIPANT DETAILS

#### Animals

All animal studies were approved by the CCHMC IACUC (Protocol# 2019–0072). Mice were housed at the vivarium at Cincinnati Children’s Hospital Medical Center under a 14h light/10h darkness schedule, 30–70% humidity and at 22.2 ± 1.1°C. An equal mix of male and female Boy J mice (C57BL/6J; CCHMC), or female NSGS (CCHMC) used in this study were 8–10 weeks old and not involved in previous procedures. Primary NSGS mice were given a single 30 mg/kg intraperitoneal dose of Busulfan 24 h prior to intravenous or intrafemoral injection. All mice were bred, housed, and handled in the Association for Assessment and Accreditation of Laboratory Animal Care-accredited animal facility of Cincinnati Children’s Hospital Medical. *Casp1*^*f/f*^ and *Casp1*^*KO*^ mice were both gifts from Chandrashekhar Pasare Laboratory. Male and female recipient and donor mice were used. Mice that appeared hunched, scruffy, lethargic, cytopenic, or distressed were euthanized in accordance with the approved IACUC protocol.

#### Human samples

Human CD34^+^ cells from healthy individuals were obtained from the Yale Cooperative Center of Excellence in Hematology (YCCEH). Human healthy peripheral blood mononuclear cells (MNCs) were purchased from Lonza. Patient AML cells were obtained from donors who gave informed consent for sample procurement under the Princess Margaret Leukemia Tissue Bank. These samples had been obtained within the framework of routine diagnostic BM aspirations after written informed consent in accordance with the Declaration of Helsinki. Human CD34^+^ patient cells were maintained in StemSpan Serum-Free Expansion Media (Cat# #09650, Stemcell Techologies) supplemented with 10 ng/mL of recombinant human stem cell factor (SCF) (Cat# 300-07-50UG, PeproTech), recombinant human thrombopoietin (TPO) (Cat# 300-18-50UG, PeproTech), recombinant human FLT3 ligand (FLT3L) (Cat# 300-19-50UG, PeproTech), recombinant human interleukin-3 (IL-3) (Cat# 200-03-50UG, PeproTech), and recombinant human interleukin-6 (IL-6) (Cat# 200-06-50UG, PeproTech).

#### Cell lines and patient-derived AML samples

Adult peripheral blood-mobilized CD34^+^ cells were purchased from the Yale School of Medicine Cooperative Center of Excellence in Hematology Cell Preparation and Analysis Core. OCI-AML2 (male), and OCI-AML3 (male) cells were purchased from DSMZ. U937 (male), TF1 (male), HL60 (female), MOLM14 (male), and THP1 (male) cells were purchased from ATCC. HEL (male) (G Huang Laboratory, CCHMC), NB4 (female) (A. Volk Laboratory, CCHMC), MV4;11 (male) (HL Grimes Laboratory, CCHMC), 293T (S. Wells Laboratory, CCHMC) and MDSL (male) (K. Tohyama Laboratory, Kawasaki Medical School, Okayama, Japan) were gifts. Analysis of short tandem repeat loci STR Profiling (Cat no. ATCC 135-XV-10, ATCC) was performed on all cell lines when received and after experimentation was complete. All cell lines were routinely tested and confirmed to be negative for mycoplasma. Previously generated patient-derived (PD) models were obtained from the Humanized Mouse Resource at Cincinnati Children’s Hospital Medical Center, as previously described.^4^ PD-AML(14) (female), PD-AML(94) (female), PD-AML(1) (not recorded), PD-AML(7) (female) were obtained from bone marrow or peripheral blood of patients at initial diagnosis with written informed consent and approval of the institutional review board of Cincinnati Children’s Hospital Medical Center and University of Cincinnati, or from the Eastern Cooperative Oncology Group (ECOG) (Table S4).

#### Institutional Animal Care and Use Committee and institutional review board

The experiments in this study were performed in compliance with the Institutional Animal Care and Use Committee (IACUC) protocols (2023–0049, 2022–0054). AML samples were obtained from bone marrow or peripheral blood of patients at initial diagnosis with written informed consent and approval of the institutional review board of Cincinnati Children’s Hospital Medical Center and University of Cincinnati. These samples had been obtained within the framework of routine diagnostic BM aspirations after written informed consent in accordance with the Declaration of Helsinki. De-identified leukemic cells from peripheral blood and BM of patients were obtained at CCHMC following consent under the IRB approved Studies (2008–0021, 2017–2224, 2023–0031).

### METHOD DETAILS

#### Culture conditions

MOLM14 were cultured in RPMI-1640 medium (Fisher, Cat# MT10040CV) with 20% FBS (Biotechne, Cat# S11550) and 1% penicillin–streptomycin (Fisher, Cat# NC2077587). U937, HEL, NB4, MV4;11, HL-60 and THP1 cells were cultured in RPMI-1640 medium with 10% FBS and 1% penicillin–streptomycin. TF1 and MDSL cells were cultured in RPMI-1640 medium with 10% FBS and 1% penicillin–streptomycin supplemented with 10 ng/mL recombinant human IL-3 (Peprotech, Cat# 200-03). OCI-AML2 and OCI-AML3 cells were cultured with Modified Eagle Medium (VWR, Cat# SH30265.FS) with 20% FBS and 1% penicillin–streptomycin. 293T cells were cultured in Dulbecco Modified Eagle Medium (Fisher, Cat# SH30022FS) with 10% FBS and 1% penicillin–streptomycin. For short-term *in vitro* culture, AML PD cells were maintained in Iscove’s modified Dulbecco’s medium (Cellgro, Cat# 10-016-CV) containing 20% FBS, 1% penicillin–streptomycin, and 10 ng/mL human SCF, TPO, FLT3L, IL3, and IL6 (Peprotech, Cat# 300-07, 300-18, 300-19, 200-03, and 200-06). Primary human mononuclear cells (MNCs) were cultured in 10% FBS Complete RPMI with 25 mM HEPES (Fisher, Cat# NC9984971) and 1mM Sodium pyruvate (Fisher, Cat# 11360070). Primary murine bone marrow monocular cells (BMNCs) were cultured in Iscove’s MDM with 10% FBS, 1% penicillin-streptomycin, and supplemented with 50 ng/mL of human IL6, TPO and FLT3L, mouse SCF (PeproTech, Cat# 250-03), and mouse IL3 (PeproTech, Cat# 213-13) cytokines.

#### Colony forming assays

One thousand human CD34^+^ BM cells, PD-AML cells, or primary AML cells per replicate were plated in methylcellulose (Stemcell Technologies, Cat# M4434) in triplicate. Colonies were scored at day 7, and for longitudinal studies, were pooled and replated at 1.0 × 10^3^ cells per replicate and scored after 7 days. For human cell lines (THP1, MDSL), five hundred cells/mL methylcellulose were plated (Stemcell Technologies, Cat# H4434) in triplicate. Colonies were quantified after 10–14 days using the STEMvision counter (StemCell Technologies). For MLL-AF9 transformed mouse BM cells, five hundred cells/mL methylcellulose were plated (Stemcell Technologies, Cat# H3434) in triplicate. Colonies were quantified after 10–14 days using the STEMvision counter (StemCell Technologies). All absolute colony numbers are included in Table S5.

#### Liquid culture growth assays

Cells were initially plated at a uniform density in 12-well plates. Cells were counted every other day by mixing 1-to-1 with trypan blue (Fisher, Cat# ICN1691049) and quantifying trypan blue-negative cells on a Countess II automated cell counter (Life Technologies). Cells were split to maintain appropriate confluency, and dilution factors were recorded to adjust cell counts. In growth assays involving drug treatment, cells were collected and resuspended in fresh media with drug every other day.

#### Immunoblot

For immunoblots, total protein lysates were obtained from cells by lysing the samples in cold RIPA buffer (50mM Tris-HCl, 150 mM NaCl, 1 mM ethylenediaminetetraacetic (EDTA), 1% Triton X-100 and 0.1% sodium dodecyl sulfate (SDS), in the presence of phenylmethylsulfonyl fluoride (PMSF), sodium orthovanadate, and protease and phosphatase inhibitors. After being resuspended in RIPA, cells were lysed by vortex followed by incubation on ice for 20 min. Protein concentration was evaluated by bicinchoninic acid (BCA) assay (Pierce, Cat#23225). SDS sample buffer was added to the lysates and the proteins were separated by SDS-polyacrylamide gel electrophoresis, transferred to PVDF or nitrocellulose membranes (BIO-RAD, Cat#1620112), and analyzed by immunoblotting. Western blot analysis was performed with the following antibodies: Vinculin (Cell Signaling, 13901T), GAPDH (Cell Signaling, #D16H11), Actin (Cell Signaling Technology, 4968), HDAC1 (Cell Signaling, #5356), Caspase-1 (Cell Signaling, #2225), Gasdermin-D (Novus Biologicals, #nbp2–33422), M2 Flag (Sigma, F3165), phospho-p65/RelA (Ser536) (Cell Signaling, #3033), Total p65/RelA (Cell Signaling, #8242), Total IκB (Cell Signaling, #4812), Raptor (Santa Cruz, #sc-81537), Phospho-p70 S6 Kinase (Thr389) (108D2) (Cell Signaling, #9234), P70 S6 Kinase (Cell Signaling, Cat No #2708), Phospho-4E-BP1 (Thr37/46) (236B4) (Cell Signaling, #2855), Total 4E-BP1 (Cell Signaling, Cat No #9644), Cereblon (Sigma, #SAB2106014), peroxidase-conjugated AffiniPure goat anti-rabbit IgG (Jackson ImmunoResearch Laboratories, #111–035-003), and peroxidase-conjugated AffiniPure goat anti-mouse IgG (Jackson ImmunoResearch Laboratories, #115–035-003). Membranes were visualized using ECL Western Blotting Substrate (Pierce, #32106) or SuperSignal West Femto Substrate (Thermo Scientific, #34096), imaged on a BIO-RAD ChemiDoc Touch Imaging system and analyzed with Image lab software 6.0.1 (Biorad) or ImageJ (22930834).

#### Co-immunoprecipitation

For Co-Immunoprecipitation, >4 mgs of protein were collected in Complete Triton Lysis Buffer (30 mM Tris-HCL, 300 mM NaCl, 1 mM EDTA, 1% Triton, 1% NP40, Halt protease inhibitor, Phosphatase inhibitor cocktail 2, Phosphatase inhibitor cocktail 3, 0.75 mM PMSF, and 0.25 mM DTT. Overnight, lysates are incubated with anti-FLAG M2 Affinity agarose beads (Sigma, Cat# A2220) at 4°C. Flag agarose beads are eluted using 3X Flag Peptide (Sigma, Cat# F4799) and then immunoblotted.

#### Flow cytometry

Red blood cells in peripheral blood, bone marrow, and dissociated spleen were lysed by incubating for 5 min in 1X PharmLyse buffer (BD Biosciences, Cat# 555899). After washing twice in 1X PBS, cells were resuspended in 2% BSA/1X PBS and analyzed by flow cytometry on a BD Biosciences FACSCanto. Cells growing in culture were washed twice in 1X PBS, and either resuspended in 2% BSA/1X PBS and analyzed by flow cytometry on a BD Biosciences FACSCanto followed by or resuspended in Sorting Buffer (Ca/Mg2+-free 1X PBS, 2% BSA, 25mM HEPES pH 7.0, 1mM EDTA) and sorted for BFP or GFP-positive cells on a BD Biosciences FACSAria. Cells analyzed by flow cytometry were incubated with the following antibodies: CD14-PE (eBiosciences, #12–0149-41), CD34-APC (Biolegend, #343510), CD38-PerCP (Biolegend, #303520). Annexin V was performed as described in the Thermofisher APC Annexin V Apoptosis Detection Kit (Cat# A35110) protocol. Cell cycle analysis was performed as described in the Click-iT EdU Alexa Fluor 647 (Cat# C10424) kit protocol, along with a DAPI stain (1:10,000)(Biolegend, Cat# 422801). Heat-shocked THP1 cells were used as positive compensation controls for DAPI-containing panels. THP1 cells (1 × 10^6^) were resuspended in PBS and heated to 95°C for 3 min. The FACS controls are in Data S2.

#### Small molecule compounds and reagents

*In vitro* 4-hydroxytamoxifen (4-OHT; Sigma, Cat# H7904), dissolved in 100% EtOH, was performed at a dose of 1 μM for 48–96 h. 100% EtOH was used as the vehicle control. *In vivo* tamoxifen treatment was performed by intraperitoneally injecting mice with 1 mg of tamoxifen (Sigma, Cat# T-5648) resuspended in corn oil each day for 5 days. Cells were dosed with VX-765 (InvitroGen, Cat# INH-VX765i-1) for 2 h before being stimulated for pyroptosis.^80^ Cells were dosed with 0.1, 1, or 10 μM VX-765 (InvitroGen, Cat# INH-VX765i-1) and 0.1, 1, or 10 μM of MCC950 (InvitroGen, Cat# INH-MCC). dCASP1–55 was synthesized by WuXi AppTech. Synthesis information and validation is provided in Data S3.

#### Pyroptosis induction

Pyroptosis was induced by stimulation with 10 ug/mL Ultrapure LPS (InvivoGen, Cat# TLRL-PEKLPS) for 24 h followed by 2 h of 20 μM Nigericin (InvivoGen, Cat# TLRL-NIG), as preformed in previous studies.^81^

#### Lactate dehydrogenase (LDH) release assay

Cells were plated at a uniform density and then treated with 24 h of 10 ug/mL LPS followed by 2 h of 20 μM Nigericin. The LDH-GloTM cytotoxicity assay (Promega, Cat# J2380) was performed according to the manufacturer’s protocol.

#### Transductions, cell sorting, and viral constructs

Assembled vectors were transformed into One Shot Stbl3 E. coli (ThermoFisher Cat #C737303) and individual colonies were expanded. Vectors were maxi-prepped and sequenced to confirm the fidelity of the inserts and then transfected into HEK-293T with. To generate virus, transfection of 293T cells (for transducing human cells) or PlatE cells (for transducing mouse cells) with viral packaging vectors and transfer plasmid was performed using TransIT-LT1 Transfection Reagent (Mirus, Cat# MIR2305) according to the manufactures’ recommendation. Third generation viral packaging plasmids pMDG.2 (Addgene, Cat# 12259) (2 μg) and psPAX.2 (Addgene, Cat# 12260) (6 μg) for lentivirus production, or pCMV-VSV-G (Cell Biolabs, Cat# RV-110) (10 μg) and pCMV-Gag-Pol (Cell Biolabs Cat# RV-111) (3 μg) for retrovirus production were used with 12 μg of transfer plasmid. The following day, the media was exchanged. After 24 h, viral supernatant was collected, filtered using a 0.45 μm filter, and stored at −80C. Cells were transduced by incubation with viral supernatant and 8μg/mL of polybrene (Millipore, Cat# TR-1003-G) overnight. GFP-positive cells were either sorted 72 h post-transduction or, in the case of MLL-AF9-transduced cells, expanded in M3434 methylcellulose every 7 days for 3 weeks. Methylcellulose-expanded cells were analyzed by flow cytometry to ensure >95% GFP-positive cells. pMSCV MLL-AF9 pGK-GFP was a gift from A. Kumar Laboratory (CCHMC). Lentiguide-BFP sgAAVS1 was a gift from L. Lee Laboratory (CCHMC) and was subcloned from LentiCRISPRV2-Cas9-GFP (Addgene #52961)^70^ by substituting the Cas9-GFP expression cassette for a mTagBFP2 cassette, thus generating a sgRNA-BFP only plasmid. pCW57.1-eGFP-APEX2-V5-EV was a gift from A. Volk Laboratory (CCHMC) and was subcloned from (#41393) by substituting the puromycin cassette for eGFP. Cas9-GFP (#51760), pSIRV-NF-kB-eGFP (#118093),^43^ pBABE-GFP Empty Vector (#10668), pBABE-IkB-superrepressor-GFP (#15263), pCDH-IRES-eGFP (#128059), pLKO.1 shSCRM-Puro (#162011),^72^ pLKO.1 shRPTOR-1-Puro (#1857),^73^ pLKO.1 shRPTOR-2-Puro (#1858),^73^ lentiCRISPRv2-Cas9-sgCRBN-puro (#166240),^51^ lentiCRISPRv2-Cas9-puro (#52961) were purchased from Addgene. pLKO.1 shCASP1–1 puro (TRCN0000003503) and pLKO.1 shCasp1–2 puro (TRCN0000003503) were purchased from MissionBio. For the pCW57.1-eGFP-APEX2-V5-CASP1, the cDNA sequences for wildtype CASP1 were optimized using the IDT Codon Optimization tool. To produce N-terminal fusions, gBlocks encoding 5′-APEX2-spacer-V5 tag-CASP1 cDNA-3′ with overhangs for Gibson assembly were obtained from IDT. NEBuilder HiFi DNA Assembly master mix (NEB Cat# E2621S) was used to Gibson assemble the CASP1 gBlock into the pCW57.1-eGFP plasmid into NheI and MluI sites.

To generate Lentiguide-BFP-sgCASP1, the sgRNA sequence (ACAGACAAGGGTGCTGAACA) was cloned into the guide RNA scaffold site on LentiCRISPRV2-sgRNA backbone (subcloned from gift from L. Lee) using a cloning strategy that has been described previously.^71^ Briefly, complementary oligos containing the desired hairpin sequence were annealed together and then cloned into the BsmBI and Esp3L sites of the LentiCRISPRV2-sgRNA (gift from L. Lee Lab). Oligo sequences are listed in Table S6.

To generate pCDH-CASP1WT-Flag-GFP vector, a WT Casp1-Flag gBlock was subcloned into the sites Nhe1 and BamHI on pCDH IRES eGFP (#128059). To generate functional CASP1 mutant vectors pCDH-CASP1-C285A-GFP and pCDH-CASP1-D5N-GFP, CASP1-C285A and CASP1-D5N gBlocks were cloned into the sites Nhe1 and BamHI on pCDH IRES eGFP (#128059). To generate CASP1 isoform vectors pCDH-IRES eGFP-CASP1Δ1–20-Flag, pCDH-IRES eGFP-CASP1Δ300–330-Flag, pCDH-IRES eGFP CASP1Δ385–403-Flag, gBlocks of Casp1 Δ1–20-Flag, Casp1 Δ300–330-Flag, Casp1 Δ385–403-Flag were subcloned into the sites Nhe1 and BamHI on pCDH-IRES-eGFP (#128059).

#### Generation of CRISPR/Cas9 mutant cells

To generate Pro-CASP1KO clones through Neon transfecting sgRNAs, THP1 and MDSL WT cells were suspended in buffer R with Cas9-NLS and three concomitant modified synthetic gRNAs targeting exon 3 of *CASP1* (Synthego; AATTAATGTCAAGGAAGATG, GGAGTACTTTCTTCCTTTCC and CTTTTCATAGATCAAACATC) and electroporated (1700 mV × 20 ms × 1 pulse) using the Neon Transfection system (Invitrogen). Transfected cells were recovered for 48 h in antibiotic-free RPMI-1640 with 1% FBS. Following recovery, transfected cells were plated in 96 well plates at a target density of 0.25 cells/well to isolate single clones. Clones were expanded and screened for CASP1 deletion by immunoblotting. Deletion was confirmed by PCR amplification of the PAM site for Sanger sequencing. Lentiviral CRISPR/Cas9 can infect a broad variety of mammalian cells by co-expressing a mammalian codon-optimized Cas9 nuclease along with a single guide RNA (sgRNA) to facilitate genome editing and was described previously.^71^ To generate Pro-CAPS1 KO cells through lentiviral transduction of sgRNAs, WT THP1, MDSL and PD-AML(1) cells were first transduced with Cas9-GFP (#51760)^71^ and sorted on GFP+ expression to obtain pure populations. Then, Cas9-GFP+ expressing cells were subsequently transduced with BFP lentiviral vectors expressing sgRNA targeting either 1) safe harbor gene AAVS1 ([Gift from L. Lee Lab, CCHMC], GGGGCCACTAGGGACAGGAT) or 2) Exon 2 of CASP1 (ACAGACAAGGGTGCTGAACA). sgCASP1-BFP and sgAAVS1-BFP transduced into Cas9-GFP-expressing THP1, MDSL and PD-AML(1) cells. THP1, MDSL and PD-AML(1) cells subsequently sorted on both BFP X GFP expression to obtain pure populations. This strategy allowed us to independently monitor Cas9 and sgRNA expression due to their separate plasmids.

#### Tiled CRISPR screen

A library of 416 sgRNAs were designed: 284 sgRNAs targeting every possible PAM sequence along the length of the CASP1 transcript, 10 non-targeting (NT) sgRNAs as controls, and 122 essential control sgRNAs. CRISPOR^82^ (https://crispor.gi.ucsc.edu/) was used to generate a pool of 284 sgRNAs optimized to contain every possible PAM sequence while also maintaining specificity to *CASP1*, a strategy used in previous screens.^53,83^ sgRNA sequences are listed in Table S7. NT and essential control sgRNA sequences were like those used in prior human CRISPR screens.^53,84^ The pooled library of sgRNA oligo sequences were cloned into a puromycin-selective pLentiguide-Puro backbone by Genscript, electroporated into Endura Electrocompetent Cells (Lucigen, Cat# 60242-1), and lentivirus was made as described previously. THP1 and MDSL cells expressing Cas9-GFP were transduced in duplicate with pooled lentivirus and selected in puromycin for 5 days at an MOI of 0.2–0.5 to achieve a gRNA coverage >30X. Cells were maintained in liquid culture for 28 days and periodically split while maintaining gRNA representation of >100X. 2 million cells were collected at each time point (Days 1, 7, 14, 21, 28), and a coverage of 4,800X (2 million cells/416 sgRNAs) was attained for each replicate sample (*N* = 2). Genomic DNA was harvested using the DNeasy Blood and Tissue kit (Qiagen Cat#69504). The genomic library was amplified and indexed following the Broad Institute protocol for PCR of sgRNAs for Illumina sequencing. The barcoded libraries were pooled and sequenced on an Illumina MiSeq at a read depth of ~21X. First, original sequencing reads were trimmed using cutadapt (v.2.1.0, https://cutadapt.readthedocs.io) before using CRISPRO^74^ [v.1.0.2] to calculate sgRNA read counts and scores based on the GRCh37-based annotation file (https://gitlab.com/bauerlab/crispro/-/tree/main/annotations). Finally, ProTiler^54^ [v.1.0.2] was used to map and visualize CRISPR knockout hyper-sensitive (CKHS) regions, which refer to the protein regions associated with a strong sgRNA dropout. CRISPRO’s guide score output files were used as input files to ProTiler.

#### APEX affinity purification

Cells transduced with pCW57.1-eGFP-APEX2-V5-EV and pCW57.1-eGFP-APEX2-V5-CASP1 were sorted on GFP expression to obtain pure populations. Inducible expression of fusion proteins and functional rescue of canonical signaling was confirmed by immunoblot. To perform APEX2 the proximity labeling, 2 × 107 doxycycline (Dox)-induced (0.5 mg/mL, 48 h) and uninduced cells were plated in 1 mL of pre-warmed media in quadruplicate. Cells were preloaded by incubating with 500 μM biotin phenol for 30 min at 37°C. The APEX2 enzyme was then activated by adding H2O2 at a final concentration of 1 mM and gently agitating for 45 s. The reaction was immediately quenched by adding quenching buffer (PBS with 10 mM sodium azide, 10 mM sodium ascorbate, 5 mM Trolox). Cells were washed with quenching buffer two additional times and then lysed and sonicated in 500 μL RIPA containing protease inhibitor and quenchers. Lysates were rotated overnight with Sera-Mag blocked streptavidin SpeedBeads (Cytiva, Cat# 21152104011150) to precipitate biotinylated proteins. Beads were washed and stored at −80°C prior to further analysis. Biotinylated proteins were extracted for the bead from the uninduced controls and dox-induced samples were eluted with Laemmli sample buffer containing 20 mM DTT. Eluents from 1 of the 4 replicates were run on an SDS gel which was stained with imperial stain to ensure capture of biotinylated proteins above background in the dox-induced samples. The remaining 3 replicates of each were prepared for digestion and mass spectrometry as described.^85^ Briefly, the eluted proteins were run into an SDS-PAGE gel for about 2 cm, then the entire protein region of each sample was excised from the gel, subjected to in-gel trypsin digestion and peptide recovery followed by nanoLC-MSMS on a Thermo Orbitrap Eclipse mass spectrometry system. Data were collected using Xcaliber 4.3 software (ThermoScientific) with label free quantitation comparative profiling of proteins detected from the uninduced control and dox-induced samples achieved using Proteome Discoverer 2.4 (ThermoScientific). Proteins with the minimum of 2 high (99%) confidence peptides with significant proteins differences (*p* < 0.05) and minimum of 2-fold charge between the groups are reported.

#### RNA-sequencing and analysis

Total RNA was extracted using a Quick-RNA MiniPrep kit (Zymo Research, Cat# R1055). RNA libraries were prepared according to the Illumina TruSeq Stranded mRNA (polyA capture) library protocol by the Genomics Sequencing Facility at CCHMC (https://www.cincinnatichildrens.org/research/shared-facilities/genomics-sequencing). The sequencing reads were aligned to the human genome hg38 using the HISAT2 (v.2.2.1, http://daehwankimlab.github.io/hisat2/) aligner.^75^ The featureCounts (v.2.0.3, https://subread.sourceforge.net/) was utilized to generate raw gene counts.^76^ Raw counts were then normalized and used to test for differential expression using edgeR,^78,86^ implemented in iGEAK.^77^ Transcription factor enrichment analysis was performed using Enrichr.^87^ Gene set enrichment analysis (GSEA) was performed as previously described.^79^ *p* values were computed from a one-sided Fisher’s exact test (hypergeometric test).

#### Humanized mouse xenograft

Experiments were performed by the Humanized Mouse Resource (HMR) at Cincinnati Children’s Hospital (https://www.cincinnatichildrens.org/research/divisions/e/ex-hem/resources/humanized). Busulfan-conditioned NSGS mice were xenografted with 500,000 human cells. Engraftment was checked on days 20 and 31 by femoral bone marrow aspirate. Moribund mice were sacrificed and assessed for leukemic burden as above. For survival analyses, time of death was recorded, and Kaplan-Meier survival analysis was performed using GraphPad Prism version 10 for Mac (GraphPad Software, www.graphpad.com).

#### Bone marrow transplantation

Male and female Boy J mice was lethally irradiated and intravenously injected with whole bone marrow containing 500,000 white blood cells from a healthy Boy J mouse (“helper cells”) along with 500,000 MLL-AF9 leukemic cells. At 4 weeks post-BM transplantation, recipient mice were injected intraperitoneally (I.P.) with 50 μL of tamoxifen (1 mg dissolved in corn oil) daily for 2 weeks to allow tamoxifen-inducible excision of floxed regions and expression of *Casp1*^*f/f*^. Engraftment was checked by femoral bone marrow aspirate two weeks following the final tamoxifen injection. At day 183, MLL-AF9 Casp1^w/w^;RosaCreER and Casp1^f/f^;RosaCreER mice underwent femoral bone marrow aspiration and were checked for leukemic burden by percent GFP-positive cells by flow cytometry. Moribund mice were sacrificed and assessed for leukemic burden. Briefly, mice were euthanized with carbon dioxide, following the AVMA Guidelines for the Euthanasia of Animals followed by cervical dislocation. Bone marrow cells were immediately extracted by crushing the leg bones (two iliac crests, two femurs, and one tibia) with a mortar and pestle. Spleens were dissociated by crushing through a 0.45μm filter. Red blood cells in peripheral blood, bone marrow, and dissociated spleen were lysed by incubating for 5 min in 1X PharmLyse buffer (BD Biosciences, Cat# 555899). Cells were washed twice in 1X PBS and then either assessed for leukemic burden. Leukemic burden was assessed by percent GFP-positive cells by flow cytometry, the presence of blasts in bone marrow and spleen cytospins and peripheral blood smears after Wright-Geimsa staining using an automated slide stainer (Siemens Hematek), appearance of the bone marrow in one tibia, spleen, and complete blood counts of peripheral blood. For survival analyses, time of death was recorded, and Kaplan Meier survival analysis was performed using GraphPad Prism version 9 for Mac (GraphPad Software, www.graphpad.com). For *Casp1*^+/+^ and *Casp1*^−/−^ BM transplantation assays, male and female Boy J mice were lethally irradiated and intravenously injected with whole bone marrow containing 500,000 white blood cells from *Casp1*^+/+^ and *Casp1*^−/−^ mice. ^88^ Peripheral blood was taken from mice at 30, 42, 54, and 71 days post-transplant, and evaluated on a Genesis Hemavet (Oxford Scientific) to quantify CBC (Complete Blood Count).

#### Clonogenic progenitor assays

BMNCs were isolated from femur and tibia, followed by RBC lysis and incubation with cKit enrichment magnetic antibodies against CD117 (Miltenyi, Cat# 130-091-224) and positively selected or purified in LS magnetic separation columns (Cat# 130-042-401). CD117+ HSCs were resuspended in Iscoves MDM (Corning, Cat#10-016-CV), and 1000 cKit+ BM were treated with 1 μM 4-OHT and plated in MethoCult GF (M3434 or M3534; StemCell Technologies). Colonies were enumerated using StemVision (StemCell Technologies) on days 12–14. To assess serial replating capacity, colonies were pooled, resuspended in Iscoves MDM, washed twice, and then replated serially at 10 × 10^3^ cells per replicate in the same medium.

#### PCR

For genotyping, genomic DNA was extracted by incubating cells with 4 parts 40mM NaOH at 95°C for 15 min, followed by the addition of 1 part 10mM Tris-HCl, pH 6.8. Polymerase chain reaction (PCR) was performed on the genomic DNA using primers flanking the DNA sequence containing flox sites and PCR master mix (Life Tech, Cat# 4359187). Thermocycler conditions used were the PCR master mix manufacturer’s recommendations.

#### Proximity ligation assays

Proximity Ligation Assays (PLA) were performed per the manufacturer’s instructions according to Duolink PLA Fluorescence Protocol (Cat# DUO92101). 80,000 THP1 and MDSL cells were washed with 1X PBS, centrifuged at 500×G, aspirated, and then resuspended in 50 uL of 1X PBS. Cells were then adhered onto Superfrost Plus Charged Slides (Fisher, Cat# 12-550-15) by cytospin at 400 rpm (THP1) and 500 rpm (MDSL) for 4 min. After, the slides were washed for 5–10 min in 1X PBS. Cells were fixed onto slides with 4% PFA in PBS for 8 min, and permeabilized with 0.1% Triton X-100 in PBS for 16 min at room temperature. Proximity Ligation Assays were conducted according to Duolink PLA Fluorescence Protocol (https://www.sigmaaldrich.com/US/en/technical-documents/protocol/protein-biology/protein-and-nucleic-acid-interactions/duolink-fluorescence-user-manual?msockid=3871123a414e6f1b16a406c840c46eb8), and primary antibodies for Caspase-1 (Cat# sc-56036) and RPTOR (Cat# ab40768) were treated at 1:100 dilution in PLA diluent provided by the manufacturer. Cells were washed in 1:10,000 DAPI in PLA wash buffer, and mounted with ProLong Diamond antifade mounting media (Cat# P36965), and were analyzed using an C2 confocal system (Nikon) attached to an inverted microscope (Nikon Eclipse Ti) equipped with a CFI S Plan Fluor ELWD 60XC oil immersion lens. Individual nuclei and PLA were counted by establishing a threshold for a binary object in each respective channel with the analysis software NIS Elements (Nikon).

#### Size exclusion chromatography (SEC)

Size exclusion chromatography studies on THP1 WT and THP1 CASP1^KO^ cells were performed as previously described.^89^ Lysates were concentrated and transferred onto a PVDF membrane and immunoblotted.

### QUANTIFICATION AND STATISTICAL ANALYSIS

The number of animals, cells, and experimental/biological replicates can be found in the figure legends. Based on our extensive experience, mouse experiments were performed using >5 recipients per condition to detect 65% relative treatment differences with 80% power at a significance level of 0.05. Differences among multiple groups were assessed by one-way analysis of variance (ANOVA) followed by Tukey’s multiple comparison posttest for all possible combinations. Comparison of two group was performed using a Student’s *t* test (unpaired, two-tailed). Unless otherwise specified, results are depicted as the mean ± standard deviation. For correlation analysis, Pearson correlation coefficient (r) was calculated. D’Agostino and Pearson and Shapiro-Wilk tests were performed to assess data distributions. For Kaplan-Meier analysis, Mantel-Cox test was used. GraphPad Prism (version 10.1.1) was used for statistical analysis. For correlative analyses, Spearman rank test was used. The number of animals, cells, and experimental/biological replicates can be found in the figure legends. Differences among multiple groups were assessed by one-way analysis of variance (ANOVA) followed by Tukey’s multiple comparison posttest for all possible combinations. Comparison of two group was performed using the Mann-Whitney test or the Student’s *t* test (unpaired, two tailed) when sample size allowed. Unless otherwise specified, results are depicted as the mean ± standard deviation or standard error of the mean. A normal distribution of data was assessed for datasets >30. For correlation analysis, Pearson correlation coefficient (r) was calculated. For Kaplan-Meier analysis, Mantel-Cox test was used. All graphs and analyses were generated using GraphPad Prism software. No data were excluded from the analyses. The experiments were not randomized, and the investigators were not blinded during experiments or outcome assessments.

## Supplementary Material

Table S1

Table S2

Table S3

Table S4

Table S5

Table S6

Table S7

Data S1-S3

1

SUPPLEMENTAL INFORMATION

Supplemental information can be found online at https://doi.org/10.1016/j.chembiol.2025.12.002.

## Figures and Tables

**KEY RESOURCES TABLE:** 

REAGENT or RESOURCE	SOURCE	IDENTIFIER

Antibodies		

Vinculin	Cell Signaling	Cat# 13901T; RRID: AB_2728768
GAPDH	Cell Signaling	Cat# 5174; RRID: AB_10622025
Pan-Actin	Cell Signaling	Cat# 4968; RRID: AB_2313904
HDAC1	Cell Signaling	Cat# 5356; RRID: AB_10612242
Caspase-1	Cell Signaling	Cat# 2225; RRID: AB_2243894
GSDMDC1	Novus Biologicals	Cat# nbp2-33422; RRID: AB_2687913
FLAG^®^ M2 antibody - clone M2	Sigma Aldrich	Cat# F3165; RRID: AB_259529
Phospho-NF-κB p65 (Ser536) (93H1)	Cell Signaling	Cat# 3033; RRID: AB_331284
NF-κB p65 (D14E12)	Cell Signaling	Cat# 8242; RRID: AB_10859369
IκBα (44D4)	Cell Signaling	Cat# 4812; RRID: AB_10694416
Raptor	Santa Cruz	Cat# sc-81537; RRID: AB_2130791
Phospho-p70 S6 Kinase (Thr389) (108D2)	Cell Signaling	Cat# 9234; RRID: AB_2269803
p70 S6 Kinase (49D7)	Cell Signaling	Cat# 2708; RRID: AB_390722
Phospho-4E-BP1 (Thr37/46) (236B4)	Cell Signaling	Cat# 2855; RRID: AB_560835
4E-BP1 (53H11)	Cell Signaling	Cat# 9644; RRID: AB_2097841
Anti-CRBN, (N-terminal)	Sigma-Aldrich	Cat# SAB2106014; RRID: AB_10744812
IL-1 beta	Abcam	Cat# ab9722; RRID: AB_308765
IKKα	Cell Signaling	Cat# 2682; RRID: AB_331626
IKKβ (2C8)	Cell Signaling	Cat# 2370; RRID: AB_2122154
Anti-MDM2 [2A10]	Abcam	Cat# ab16895; RRID: AB_2143534
Peroxidase-conjugated AffiniPure goat anti-rabbit IgG	Jackson ImmunoResearch	Cat# 111-035-003; RRID: AB_2313567
CD14-PE	eBiosciences	Cat# 12-0149-41; RRID: AB_10597598
CD34-APC	Biolegend	Cat# 343510; RRID: AB_1877153
CD38-PerCP	Biolegend	Cat# 303520; RRID: AB_893313
APC Annexin V	Thermofisher	Cat# A35110; RRID: AB_3720098
Alexa Fluor 647 EdU	Fisher	Cat# C10424; RRID: AB_3720099
Caspase-1 (14F468)	Santa Cruz	Cat# sc-56036; RRID: AB_781816
Anti-Raptor antibody [EP539Y]	Abcam	Cat# ab40768; RRID: AB_777622

Bacterial and virus strains		

VSVG lenti- and retrovirus	Cincinnati Children’s Applied Gene and Cell Therapy Center	https://www.cincinnatichildrens.org/research/divisions/a/applied-gene-cell-therapy/services
One Shot^™^ Stbl3^™^ E. coli	ThermoFisher	Cat# C737303
Endura Electrocompetent Cells	Lucigen	Cat# 60242-1

Biological samples		

Normal human CD34^+^ cells	Yale Cooperative Center of Excellence in Hematology	N/A
Human healthy peripheral blood mononuclear cells (MNCs)	Lonza	N/A
AML (11) Patient Sample	University of Cincinnati Cancer Center/Cincinnati Children’s Cancer and Blood Disease Institute	N/A
AML (16) Patient Sample	University of Cincinnati Cancer Center/Cincinnati Children’s Cancer and Blood Disease Institute	N/A
AML (81) Patient Sample	University of Colorado Cancer Center	N/A
AML (12) Patient Sample	University of Cincinnati Cancer Center/Cincinnati Children’s Cancer and Blood Disease Institute	N/A

Chemicals, peptides, and recombinant proteins		

StemSpan Serum-Free Expansion Media	Stem Cell Technologies	Cat# 09650
Methocult H4434 Classic	Stem Cell Technologies	Cat# 04434
Methocult H4435 Enriched	Stem Cell Technologies	Cat#12-1899
Methocult GF M3434	Stem Cell Technologies	Cat# 03434
RPMI-1640	Fisher	Cat# MT10040CV
IMDM	Cellgro	Cat# 10-016-CV
DMEM	Fisher	Cat# SH30022FS
Modified Eagle Medium (MEM)-alpha	VWR	Cat# 200-03
Fetal Bovine Serum (FBS)	Biotechne	Cat# S11550
Penicillin/Steptomycin	ThermoFisher	Cat# SV30010
Human stem cell factor (SCF)	PeproTech	Cat# 300-18-50UG
Human FLT3 ligand (FLT3L)	PeproTech	Cat# 300-19-50UG
Human interleukin-3 (IL-3)	PeproTech	Cat# 200-03-50UG
Human interleukin-6 (IL-6)	PeproTech	Cat# 200-06-50UG
Human Thrombopoietin (TPO)	PeproTech	Cat# 300-18
HEPES	Fisher	Cat# NC9984971
Sodium pyruvate	Fisher	Cat# 11360070
Mouse stem cell factor (SCF)	PeproTech	Cat# 250-03
Mouse interleukin-3 (IL-3)	PeproTech	Cat# 213-13
TransIT^®^-LT1 Transfection Reagent	Mirus	Cat# MIR2305
Polybrene	Sigma-Aldrich	Cat# TR-1003-G
NEBuilder HiFi DNA Assembly master mix	New England Biosciences	Cat# E2621S
VX-765	Invitrogen	Cat# INH-VX765i-1
MCC950	Invitrogen	Cat# INH-MCC
RG-7112	VWR SelleckChem	Cat# S7030
dCASP1-55	This Study, WuXi AppTech	N/A
Precision Plus Protein Dual Color Standards (Ladder)	BioRad	Cat# 1610374
PageRuler Prestained Protein Ladder, 10 to 180 kDa	ThermoFisher	Cat# 26616
Ultrapure LPS	Invivogen	Cat# TLRL-PEKLPS
Nigericin	Invivogen	Cat# TLRL-NIG
Doxycycline Hydrochloride	Fisher	Cat# BP26535
4-hydroxytamoxifen (4-OHT)	Sigma-Aldrich	Cat# H7904
Tamoxifen	Sigma-Aldrich	Cat# T-5648
CD117 MicoBeads	Miltenyi Biotech	Cat# 130-091-224
3X FLAG(R) Peptide	Sigma-Aldrich	Cat# F4799-4MG
ECL Western Blotting Substrate	Pierce	Cat# 32106
DAPI (4′,6-Diamidino-2-Phenylindole, Dilactate)	Biolegend	Cat# 422801
Sera-Mag blocked streptavidin Speed Beads	Cytiva	Cat# 21152104011150
ANTI-FLAG M2 Affinity Gel	Sigma-Aldrich	Cat# A2220
1X PharmLyse buffer	BD Biosciences	Cat# 555899
Trypan Blue	Fisher	Cat# ICN1691049
Superose 6 Increase 3.2/300 column	GE Healthcare	Cat# 29-0915-98
Gel filtration calibration kits	GE	Cat#28-4038-41
Gel filtration calibration kits	GE	Cat#28-4038-42
Gel filtration calibration kits	Sigma	Cat#MWGF200-1KT
LS Columns	Miltenyi Biotech	Cat# 130-042-401
Duolink *In Situ* Detection Reagents Green	Millipore Sigma	Cat# DUO92014
Superfrost Plus Charged Slides	Thermofisher	Cat# 12-550-15
ProLong Gold antifade mounting media	Thermofisher	Cat# P36965

Critical commercial assays		

LDH-Glo Cytotoxicity Assay	Promega	Cat# J2380
Quick-RNA MiniPrep	ZymoResearch	Cat# R1055
PCR master mix	Life Tech	Cat# 4359187
PE Annexin V Apoptosis Detection kit	BD Pharmingen	Cat# 559763
Click-iT^™^ EdU Alexa Fluor^™^ 647 Flow Cytometry Assay Kit	Fisher	Cat# C10424
High-Capacity cDNA Reverse Transcription Kit	ZymoResearch	Cat# R1055
DNeasy Blood and Tissue kit	Quiagen	Cat# 69504
Duolink PLA Fluorescence Protocol	Millipore Sigma	Cat# DUO92101

Deposited data		

Bulk RNAseq profiling of WT MDSL or Casp1KO MDSL cells generated by Crispr Cas9 and grown in liquid culture. GEO Token: kxwjcocahdgzdoh	This Study	GEO: GSE294653 https://www.ncbi.nlm.nih.gov/geo/query/acc.cgi?acc=GSE294653
Bulk RNAseq profiling of THP1-Cas9 cells transduced with Lentiviral delivery vectors specific for safe harbor control gene sgAAVS1 (WT), or sgCASP1 (KO). Token: epqboykkdxkbfct	This Study	GEO: GSE294654 https://www.ncbi.nlm.nih.gov/geo/query/acc.cgi?acc=GSE294654
Gene expression data from bone marrow CD34^+^ cells of patients with myelodysplastic syndromes (MDS) and healthy controls	Gerstung et al.^69^	GEO: GSE58831 https://www.ncbi.nlm.nih.gov/geo/query/acc.cgi?acc=GSE58831

Experimental models: Cell lines		

OCI-AML2	DSMZ; authenticated	RRID:CVCL_1619
OCI-AML3	DSMZ; authenticated	RRID:CVCL_1844
THP-1	ATCC; authenticated	ATC - TIB-202
MV4;11	ATCC; authenticated	ATC - CRL-9591
U-937	ATCC; authenticated	ATC CRL-1593.2
TF-1	ATCC; authenticated	ATC CRL-2003
HL-60	From Aly Karsan Laboratory; authenticated	ATC CCL-240
MOLM-14	From Neil Shah Laboratory; authenticated	RRID:CVCL_7916
293T	From Susanne Wells Laboratory; authenticated	ATC CRL-3216
MDS-L	From Kaoru Tohyama Laboratory; authenticated	RRID:CVCL_A8QV
HEL	From Gang Huang Laboratory; authenticated	ATC TIB-180
NB-4	From Andrew Volk Laboratory	RRID:CVCL_0005
PD-AML(7) [AML (2016-7)]	Humanized Mouse Resource at Cincinnati Children’s Hospital Medical Center	https://www.cincinnatichildrens.org/research/divisions/e/ex-hem/resources/humanized
PD-AML(94) [AML (2017-94)]	Humanized Mouse Resource at Cincinnati Children’s Hospital Medical Center	https://www.cincinnatichildrens.org/research/divisions/e/ex-hem/resources/humanized
PD-AML (14) [AML (2017-14)]	Humanized Mouse Resource at Cincinnati Children’s Hospital Medical Center	https://www.cincinnatichildrens.org/research/divisions/e/ex-hem/resources/humanized
PD-AML (1) AML (2016-1)]	Humanized Mouse Resource at Cincinnati Children’s Hospital Medical Center	https://www.cincinnatichildrens.org/research/divisions/e/ex-hem/resources/humanized

Experimental models: Organisms/strains		

Mice: *Casp1^f/f^* mice. C57/B6	From Chandrashekhar Pasare Laboratory	N/A
Mice: *Casp1^KO^* mice. C57/B6	From Chandrashekhar Pasare Laboratory	N/A
Mice: Tet2^f/f^; *Casp1*^*f*/f^;RosaCreERT2 mice. C57/B6	This study	N/A
Mice: Tet2^f/f^;RosaCreERT2 mice. C57/B6	This study and Muto et al.^36^	N/A
Mice: *Casp1*^*f*/f^;RosaCreERT2 mice. C57/B6	This study	N/A

Oligonucleotides		

See Table S6 for Oligonucleotide sequences	This study	N/A

Recombinant DNA		

pMDG.2	pMD2.G was a gift from Didier Trono	Addgene: Cat# 12259
psPAX2	psPAX2 was a gift from Didier Trono	Addgene: Cat# 12260
pCMV-VSV-G Envelope Vector	Cell Biolabs	Cat# RV-110
pCMV-Gag-Pol Vector	Cell Biolabs	Cat# RV-111
pMSCV MLL-AF9 pGK-GFP	Ashish Kumar Laboratory	N/A
Lentiguide-BFP sgAAVS1	Lynn Lee Laboratory	N/A
LentiCRISPRV2-Cas9	Sanjana et al.^70^	Addgene: Cat# 52961
LentiCRISPR-Cas9-GFP	Shalem et al.^71^	Addgene: Cat# 51760
pCW57.1_eGFP_APEX2_V5_EV	Andrew Volk Laboratory	N/A
pSIRV-NF-kB-eGFP	Jutz et al.^43^	Addgene: Cat# 118093
pBABE GFP (Empty Vector)	pBABE GFP was a gift from William Hahn	Addgene: Cat# 10668
pBABE_IkB Superrepressor_GFP	Boehm et al.^52^	Addgene: Cat# 15264
pCDH EF1-IRES-eGFP	pCDH-EF1-IRES eGFP was a gift from Oskar Laur	Addgene: Cat# 128059
pLKO.1 shSCRM_Puro	Roman-Fernandez et al.^72^	Addgene: Cat# 162011
pLKO.1 shRPTOR-1_Puro	Sarbassov et al.^73^	Addgene: Cat# 1857
pLKO.1 shRPTOR-2_Puro	Sarbassov et al.^73^	Addgene: Cat# 1858
lentiCRISPRv2_Cas9_sgCRBN_puro	Lier et al.^51^	Addgene: Cat# 166240
lentiCRISPRv2_Cas9_sgControl_puro	Sanjana et al.^70^	Addgene: Cat# 52961
pLKO.1 shCASP1_1 puro	MissionBio	TRCN# 0000003503
pLKO.1 shCasp1_2 puro	MissionBIo	TRCN# 0000003503
pCW57.1_eGFP_APEX2_V5_WTCASP1	This Study	N/A
pCDH_CASP1[C285A]-GFP	This Study	N/A
pCDH-CASP1-D5N[GFP]	This Study	N/A
pCDH IRES eGFP_CASP1Δ1-20-Flag	This Study	N/A
pCDH IRES eGFP_CASP1Δ300-330-Flag	This Study	N/A
pCDH IRES eGFP_CASP1Δ385-403-Flag	This Study	N/A
pCW57.1_eGFP_APEX2_V5_EV	This Study	N/A
pCW57.1_eGFP_APEX2_V5_WTCASP1a	This Study	N/A

Software and algorithms		

Prism 10	GraphPad	N/A
FlowJo 10	BD BioSciences	N/A
Image Lab	BioRad	https://www.bio-rad.com/en-us/product/image-lab-software?ID=KRE6P5E8Z
ImageJ	National Institutes of Health	https://imagej.nih.gov/ij/
CRISPOR	Schoonenberg et al.^74^	https://crispor.gi.ucsc.edu/
Cutadapt [v.2.1.0]	Schoonenberg et al.^74^	https://cutadapt.readthedocs.io)
CRISPRO [v.1.0.2]	Schoonenberg et al.^74^	N/A
Pro-Tiler [v.1.0.2]	He et al.^54^	N/A
Xcaliber 4.3 software	Thermofisher	https://www.thermofisher.com/us/en/home/industrial/mass-spectrometry/liquid-chromatography-mass-spectrometry-lc-ms/lc-ms-software/lc-ms-data-acquisition-software/xcalibur-data-acquisition-interpretation-software.html
Proteome Discoverer 2.4	Thermofisher	https://www.thermofisher.com/us/en/home/industrial/mass-spectrometry/liquid-chromatography-mass-spectrometry-lc-ms/lc-ms-software/multi-omics-data-analysis/proteome-discoverer-software.html
HISAT2 (v.2.2.1)	Kim et al.^75^	http://daehwankimlab.github.io/hisat2/
featureCounts (v.2.0.3)	Liao et al.^76^	https://subread.sourceforge.net/
iGEAK	Choi et al.^77^	https://sites.google.com/view/iGEAK
edgeR	Chen et al.^78^	https://bioconductor.org/packages/release/bioc/html/edgeR.html
Enricher	Chen et al.^78^	https://maayanlab.doud/Enrichr/
GSEA	Subramanian et al.^79^	https://www.gsea-msigdb.org/gsea/index.jsp
AlphaFold	Varadi et al.^56^	https://alphafold.ebi.ac.uk/
PAE Viewer	Elfmann et al.^57^	https://pae-viewer.uni-goettingen.de/
Nikon NIS Elements	Nikon	https://www.microscope.healthcare.nikon.com/products/software/nis-elements

Other		

Nitrocellulose membrane	Bio-Rad	Cat# 162-0112
PVDF membrane	Millipore Sigma	Cat# IPVH00010

## SIGNIFICANCE

Inflammation contributes to the development and progression of many blood cancers, yet the specific inflammatory pathways that support leukemic growth remain unclear. Caspase-1 (CASP1) is best known for promoting cytokine activation and pyroptotic cell death, but its broader role in leukemia has not been fully explored. Here, we identify an unexpected, non-enzymatic role for CASP1 as an essential scaffolding factor in leukemia. CASP1 enables leukemia cells to coordinate signaling between RAPTOR/mTORC1 and NF-κB, two pathways broadly involved in inflammation and cancer progression. Disrupting CASP1, either genetically or through domain-specific mutations, interferes with RAPTOR function and drives excessive NF-κB activity, impaired survival, and loss of self-renewal. Moreover, we developed a CASP1 degrader that selectively suppresses leukemic growth, demonstrating that CASP1’s scaffolding function is a druggable vulnerability. These findings expand the understanding of inflammasome-related proteins by revealing a non-catalytic role for CASP1 in cancer and establish CASP1 as a therapeutic target in myeloid malignancies. More broadly, our results suggest that targeting the structural or organizational functions of inflammatory enzymes may offer new opportunities for therapeutic intervention across cancers driven by dysregulated inflammatory signaling.

## Highlights

- CASP1 loss impairs leukemia growth independent of IL-1β and pyroptosis
- CASP1 scaffolds RPTOR to prevent excessive mTORC1-NF-κB signaling
- The CARD domain, not catalytic activity, of CASP1 is essential for leukemic cells
- PROTAC degrader, dCASP1–55, targets Pro-CASP1 and suppresses leukemic cells
