# Supplementary material for: Scaffolding-dependent CASP1 constrains excessive cell-intrinsic inflammatory signaling in leukemia
Source: Cell Chem Biol. Author manuscript; Available in PMC 2026 Jun 28. (PMC13310425; doi:10.1016/j.chembiol.2025.12.002)
Supplement: Data S1-S3 [file NIHMS2173962-supplement-Data_S1-S3.pdf]

Data S1: Source data, related to Figure 1

Fig 1D

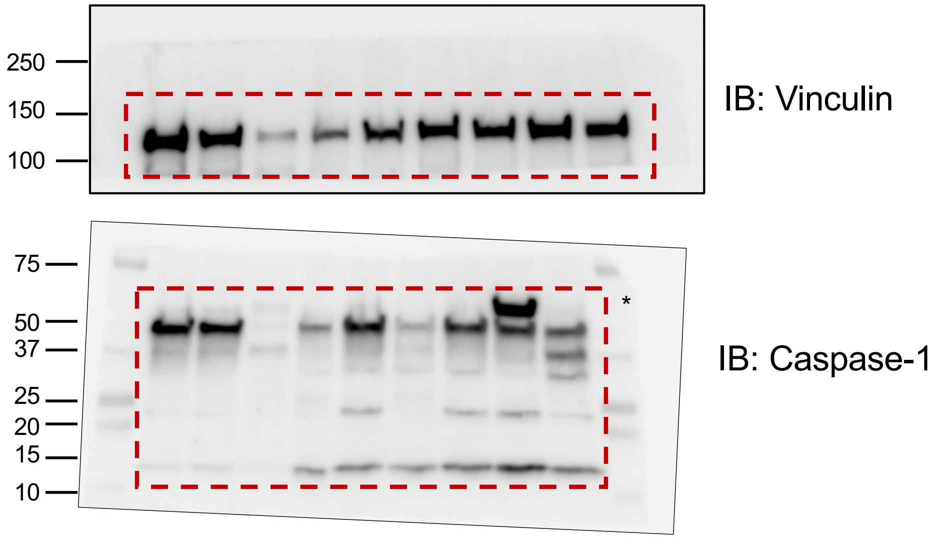

*, non-specific band

Fig 1E

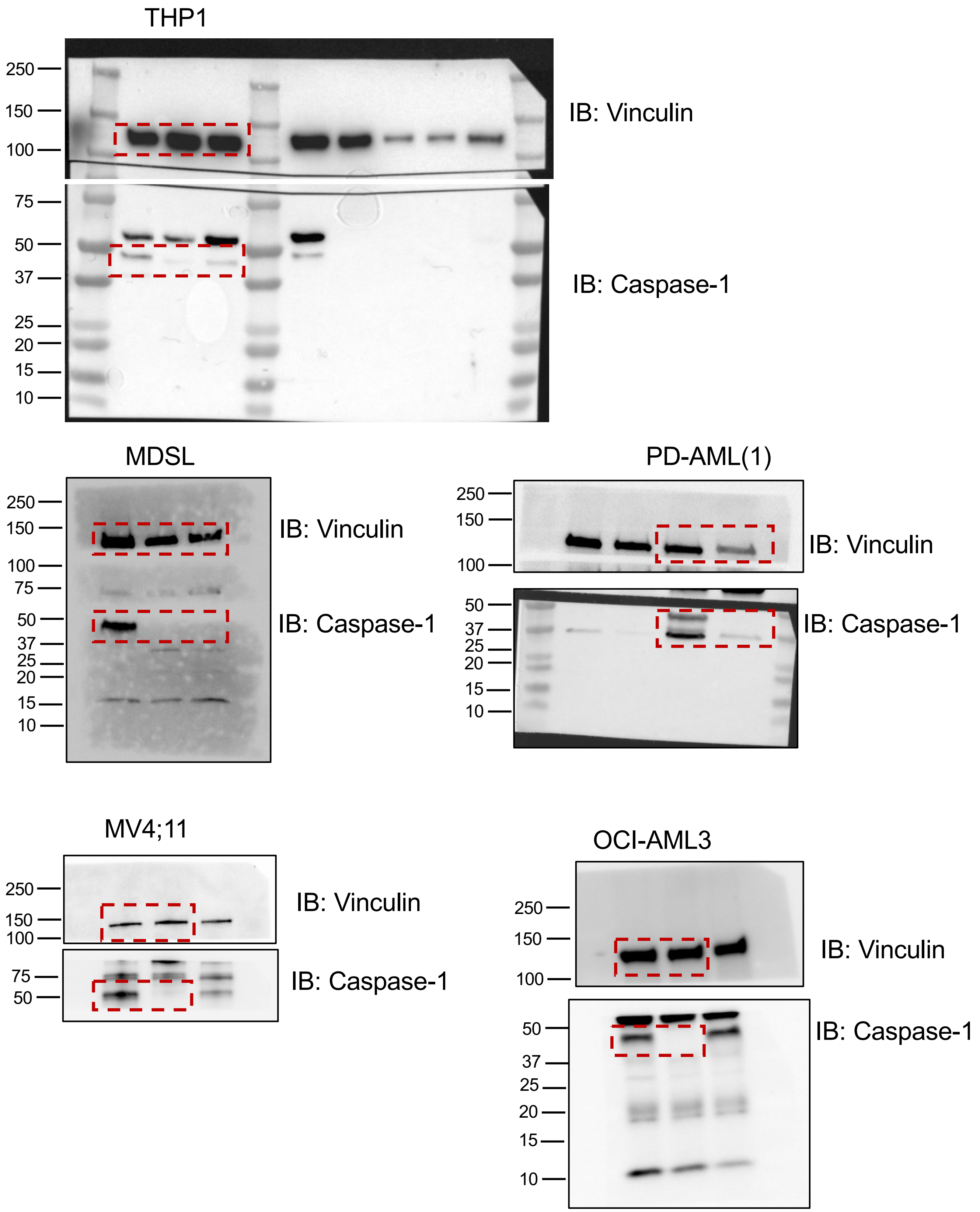
THP1
250
150
100
IB: Vinculin
75
50
37
25
20
15
10
IB: Caspase-1
MDSL
250
150
100
75
50
37
25
20
15
10
IB: Vinculin
IB: Caspase-1
PD-AML(1)
250
150
100
IB: Vinculin
50
37
25
20
15
10
IB: Caspase-1
MV4;11
250
150
100
IB: Vinculin
75
50
IB: Caspase-1
OCI-AML3
250
150
100
IB: Vinculin
50
37
25
20
15
10
IB: Caspase-1

Data S1: Source data, related to Figure 2

Fig 2B

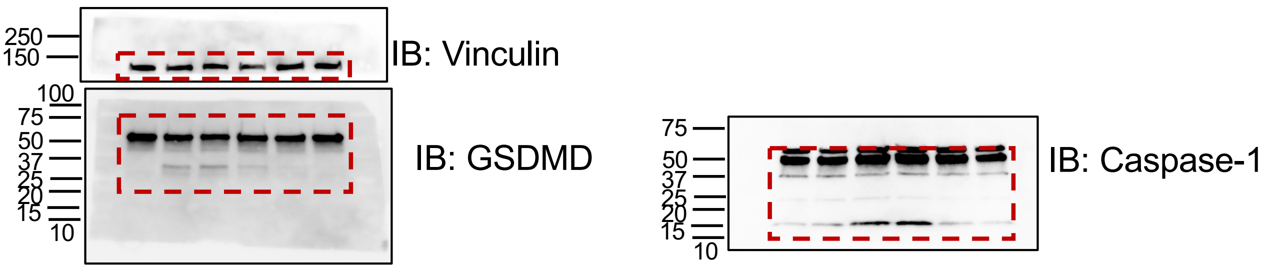

Fig 2E

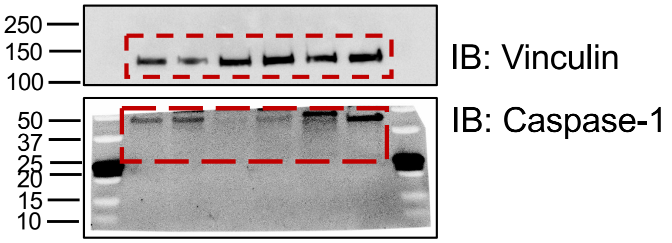

Fig 2I

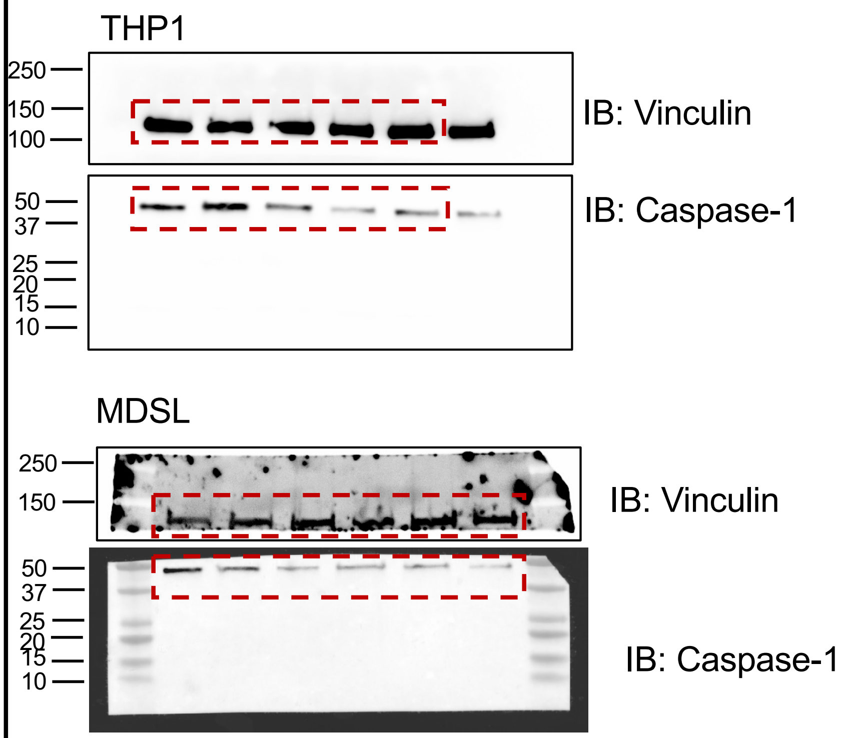

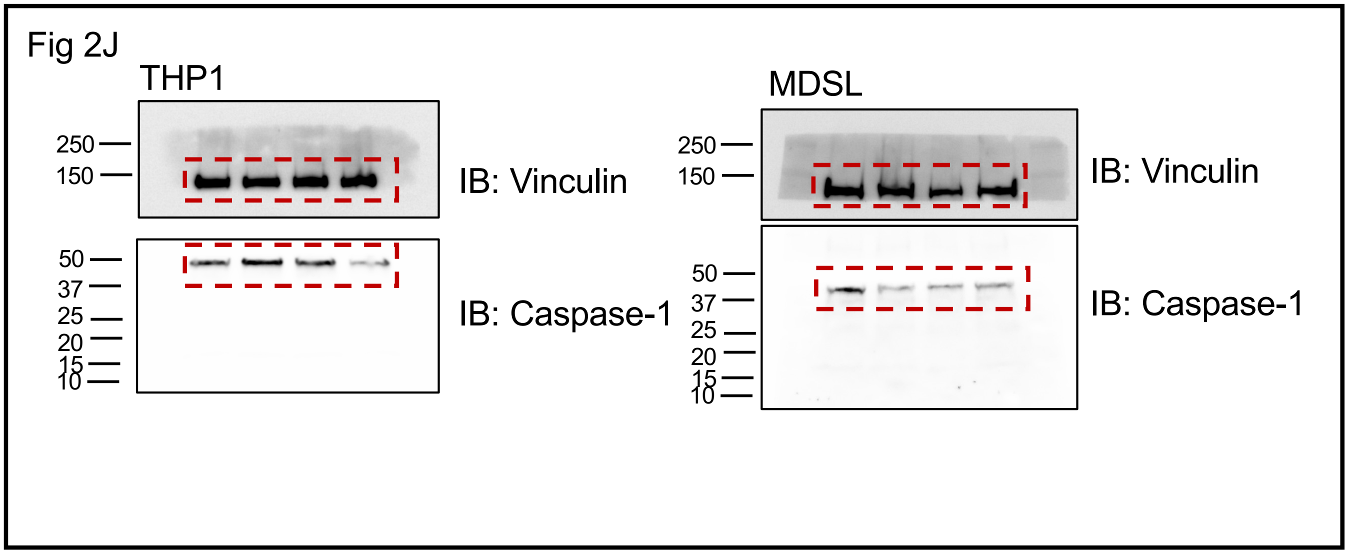
Fig 2J
THP1
250
150
IB: Vinculin
50
37
25
20
15
10
IB: Caspase-1
MDSL
250
150
IB: Vinculin
50
37
25
20
15
10
IB: Caspase-1

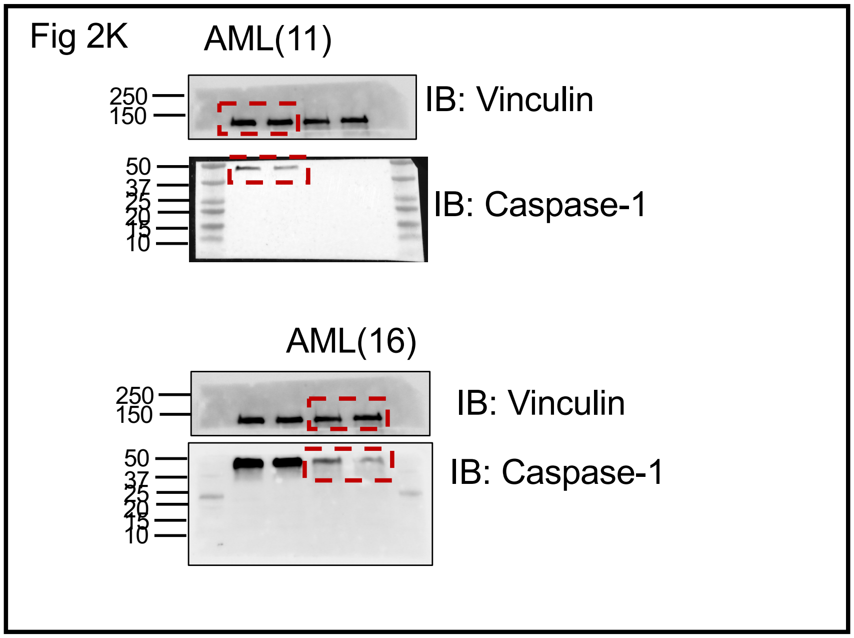
Fig 2K
AML(11)
250
150
IB: Vinculin
50
37
25
20
15
10
IB: Caspase-1
AML(16)
250
150
IB: Vinculin
50
37
25
20
15
10
IB: Caspase-1

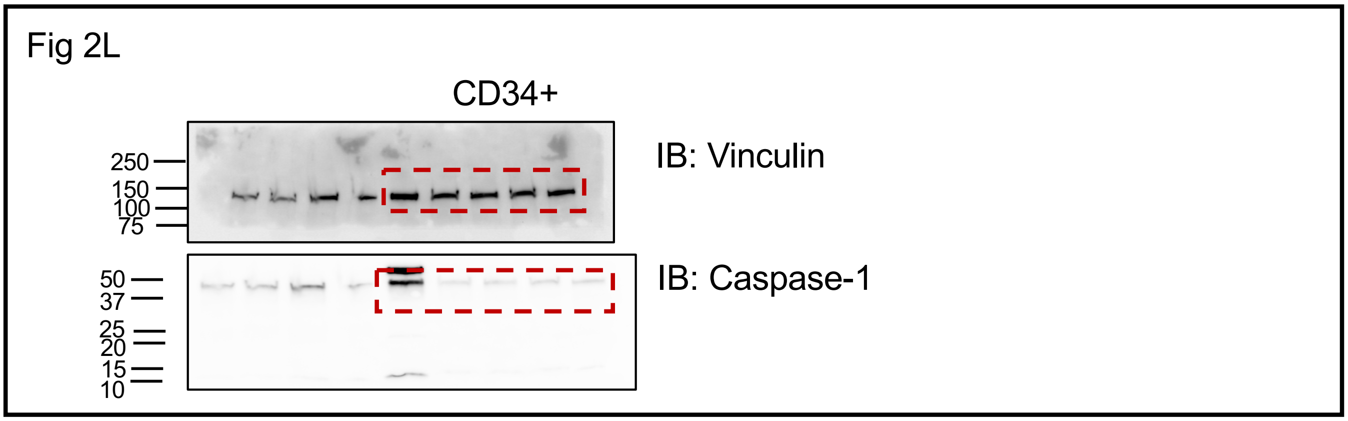
Fig 2L
CD34+
250
150
100
75
IB: Vinculin
50
37
25
20
15
10
IB: Caspase-1

Data S1: Source data, related to Figure 3

Fig 3D

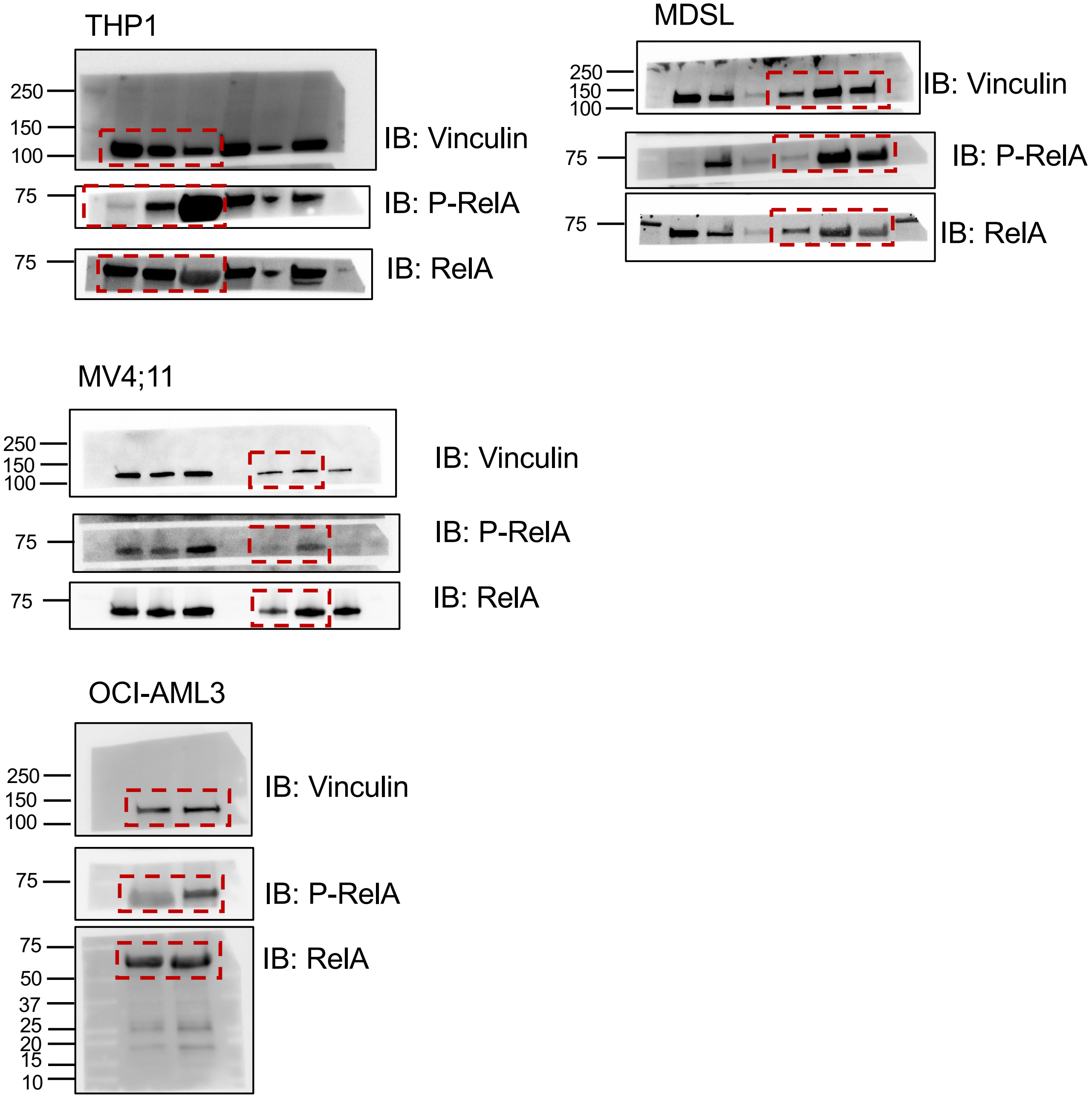

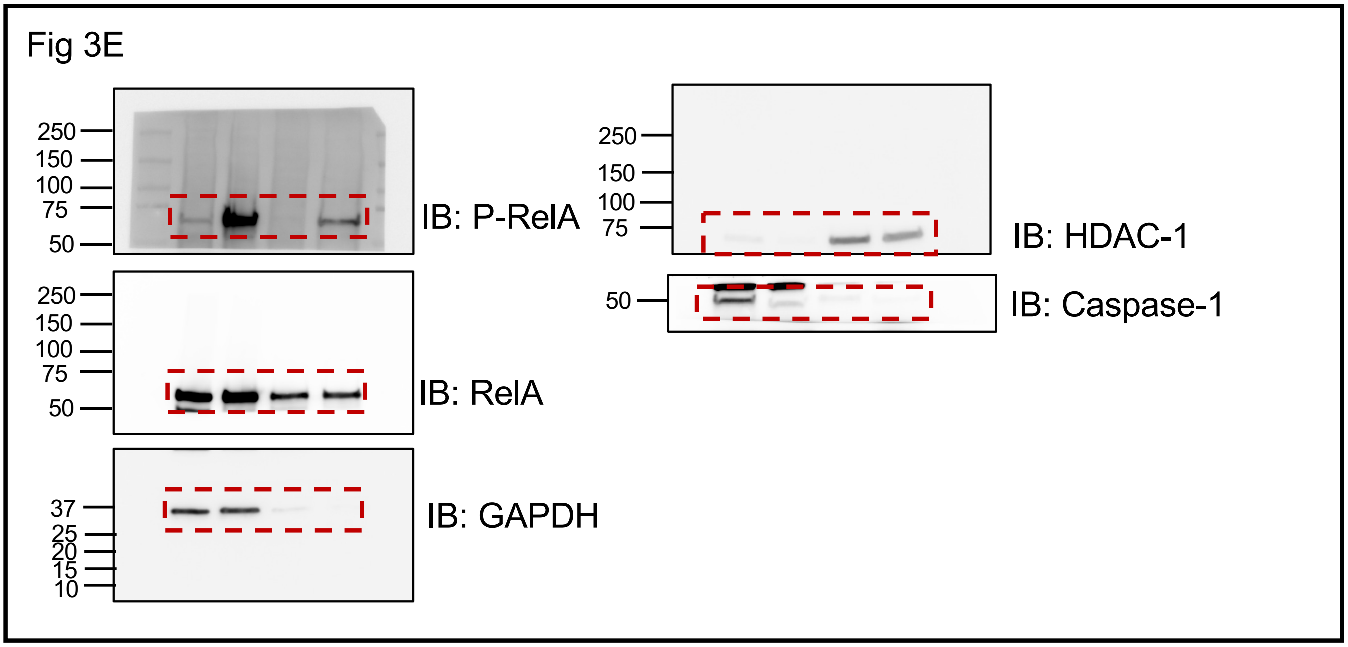
Fig 3E
250
150
100
75
50
IB: P-RelA
IB: RelA
37
25
20
15
10
IB: GAPDH
IB: HDAC-1
IB: Caspase-1

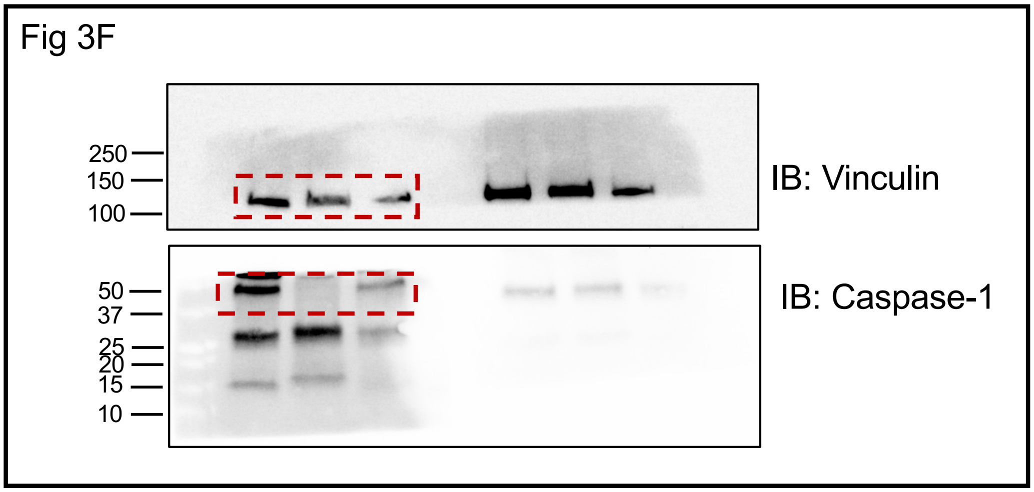
Fig 3F
250
150
100
IB: Vinculin
50
37
25
20
15
10
IB: Caspase-1

Fig 3H

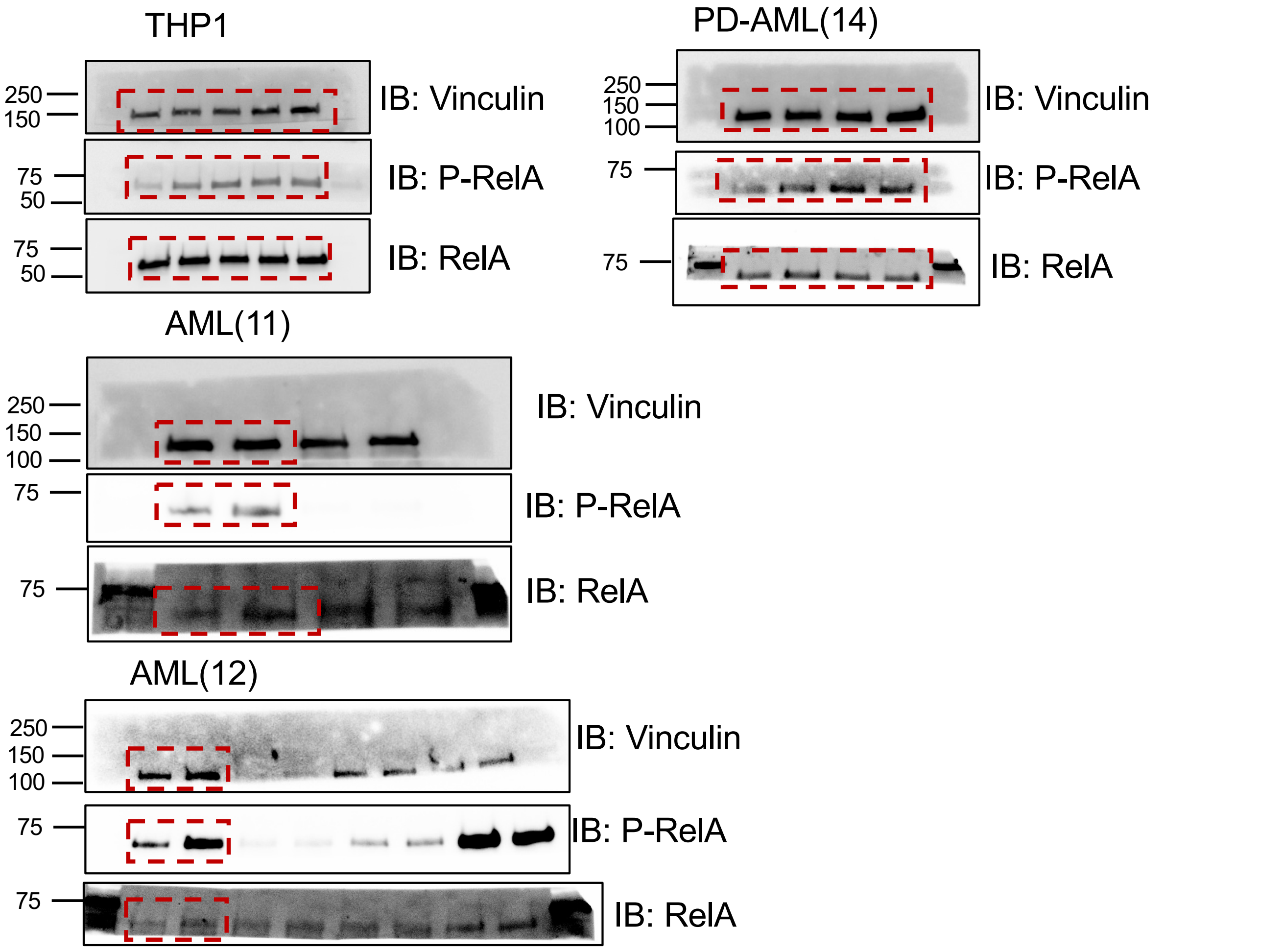
THP1
250
150
IB: Vinculin
75
50
IB: P-RelA
75
50
IB: RelA
PD-AML(14)
250
150
100
IB: Vinculin
75
IB: P-RelA
75
IB: RelA
AML(11)
250
150
100
IB: Vinculin
75
IB: P-RelA
75
IB: RelA
AML(12)
250
150
100
IB: Vinculin
75
IB: P-RelA
75
IB: RelA

Fig 3I

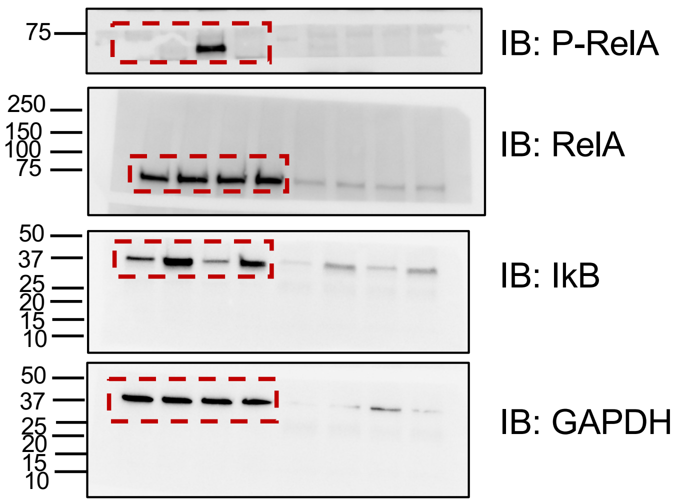
75
IB: P-RelA
250
150
100
75
IB: RelA
50
37
25
20
15
10
IB: IkB
50
37
25
20
15
10
IB: GAPDH

Data S1: Source data, related to Figure 4

Fig 4D

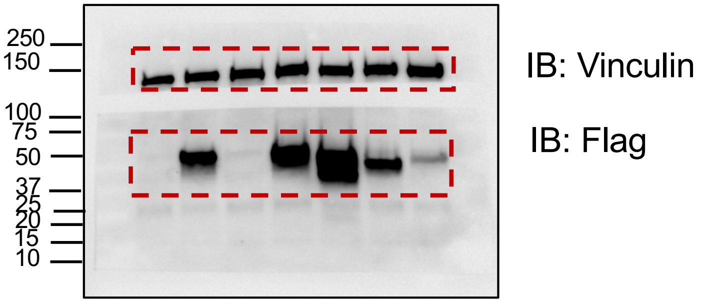

Fig 4I

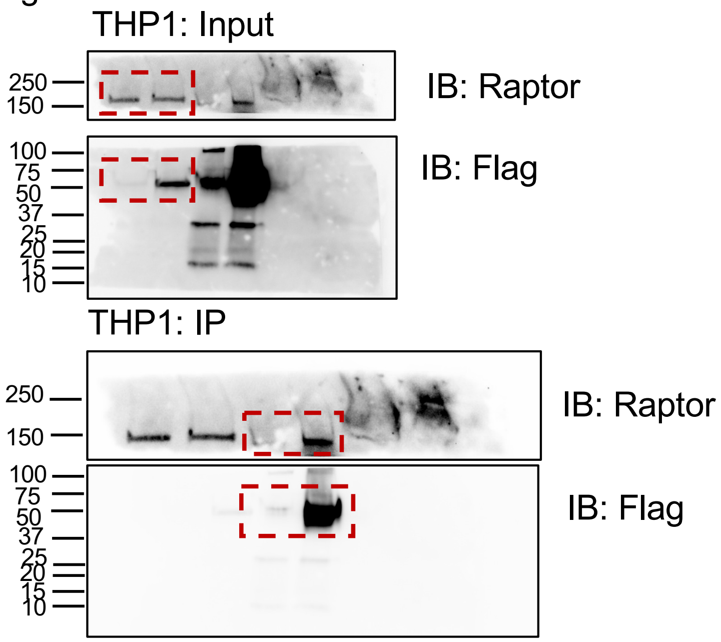

Fig 4L

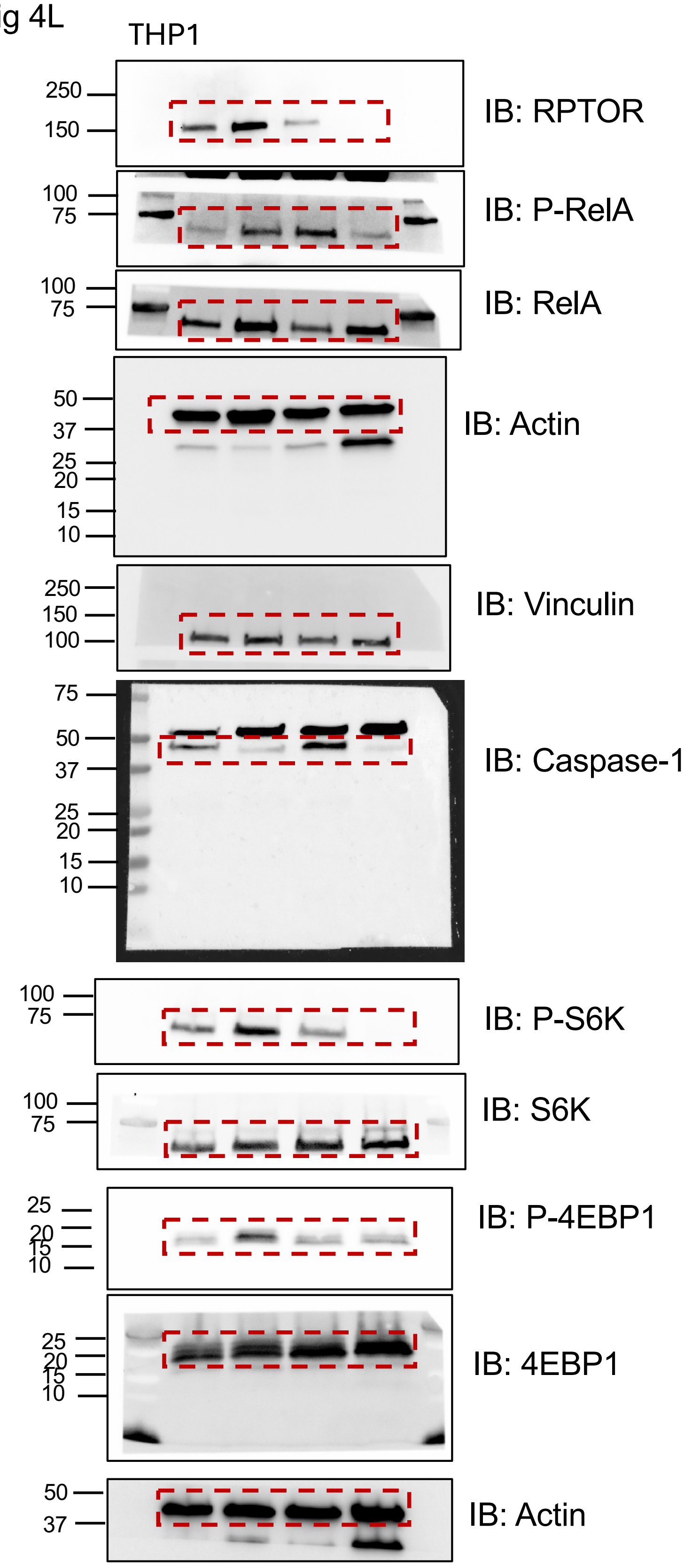
THP1
250
150
IB: RPTOR
100
75
IB: P-RelA
100
75
IB: RelA
50
37
25
20
15
10
IB: Actin
250
150
100
IB: Vinculin
75
50
37
25
20
15
10
IB: Caspase-1
100
75
IB: P-S6K
100
75
IB: S6K
25
20
15
10
IB: P-4EBP1
25
20
15
10
IB: 4EBP1
50
37
IB: Actin

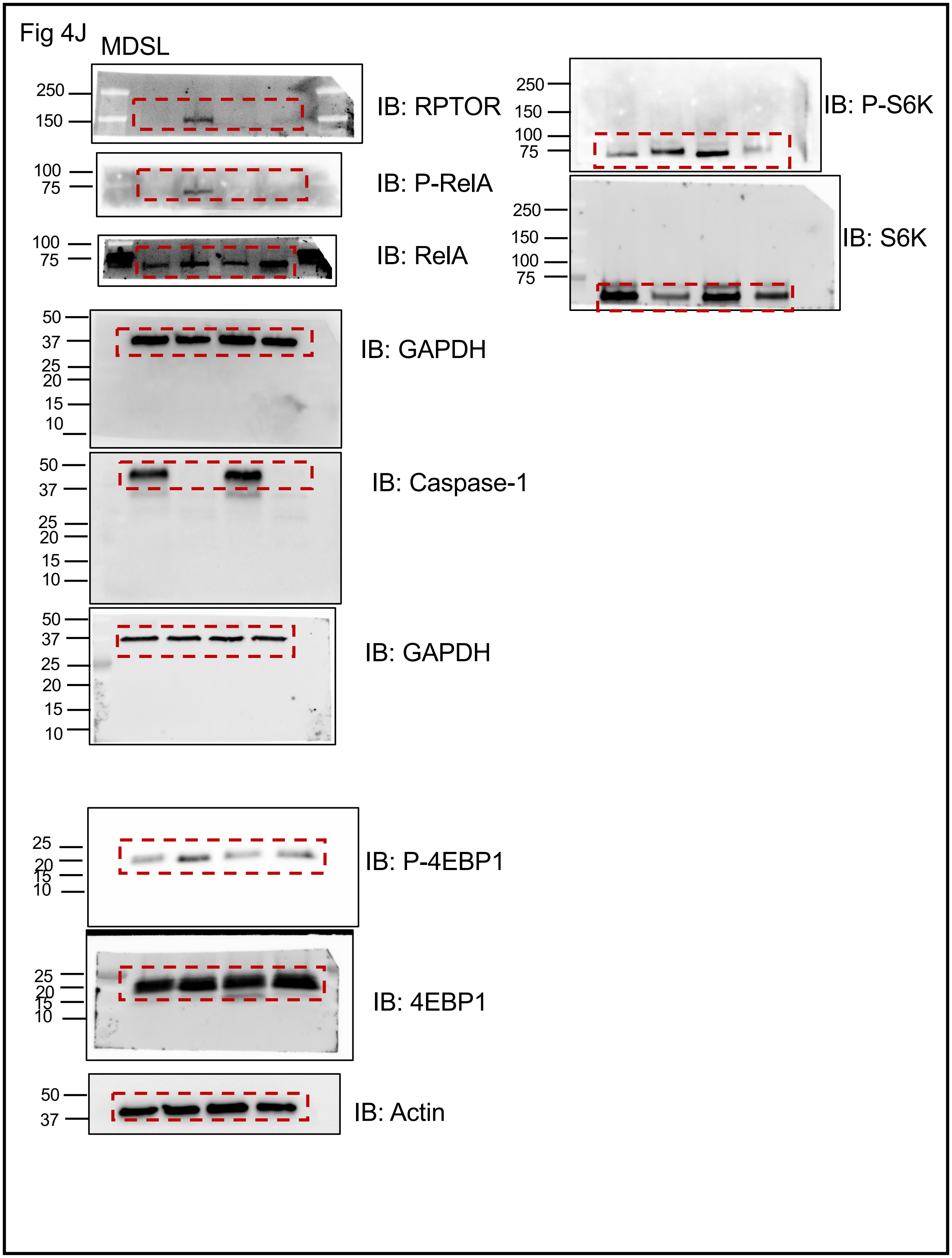
Fig 4J
MDSL
250
150
IB: RPTOR
100
75
IB: P-RelA
100
75
IB: RelA
50
37
25
20
15
10
IB: GAPDH
50
37
25
20
15
10
IB: Caspase-1
50
37
25
20
15
10
IB: GAPDH
25
20
15
10
IB: P-4EBP1
25
20
15
10
IB: 4EBP1
50
37
IB: Actin
250
150
100
75
IB: P-S6K
250
150
100
75
IB: S6K

Data S1: Source data, related to Supplemental Figure 1

Supplemental Fig 1G

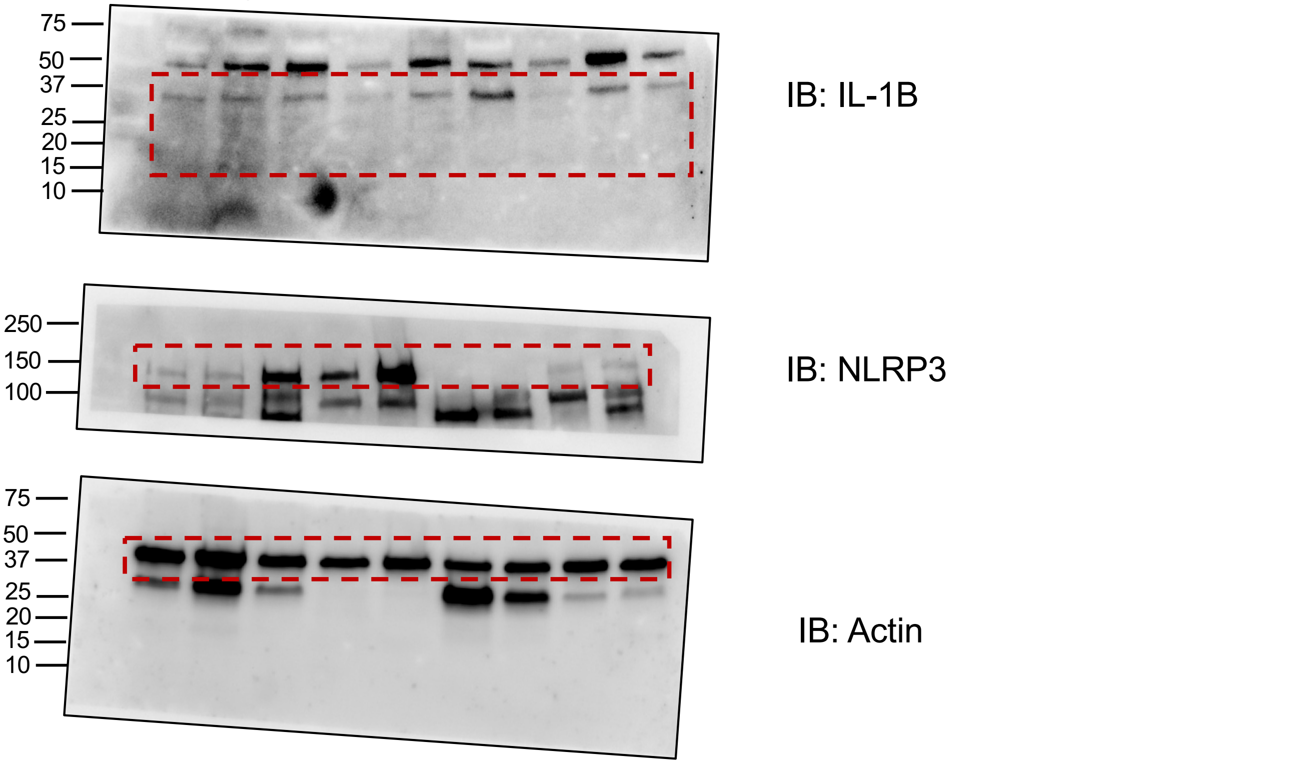

Supplemental Fig 1I

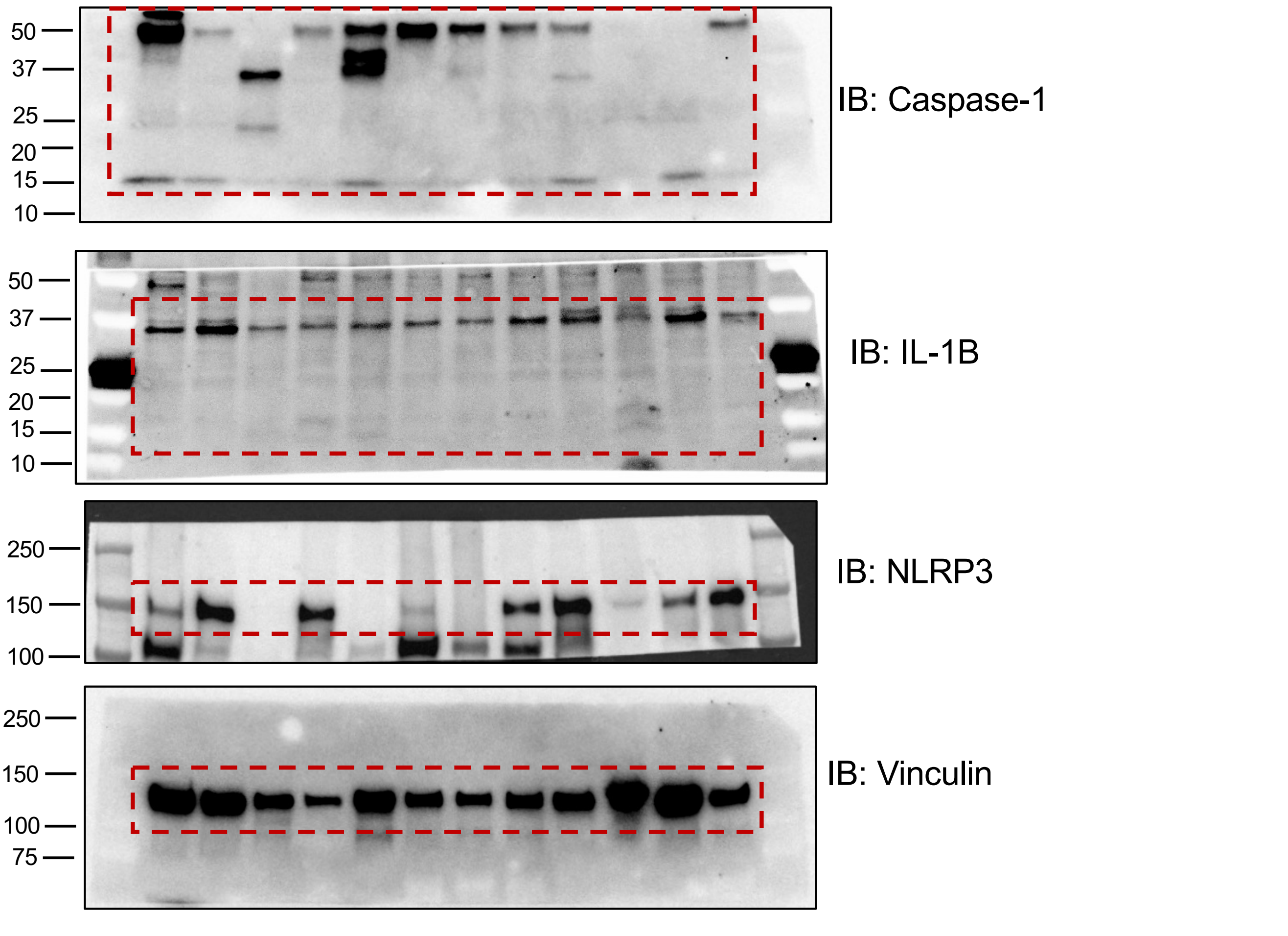

Data S1: Source data, related to Supplemental Figure 2

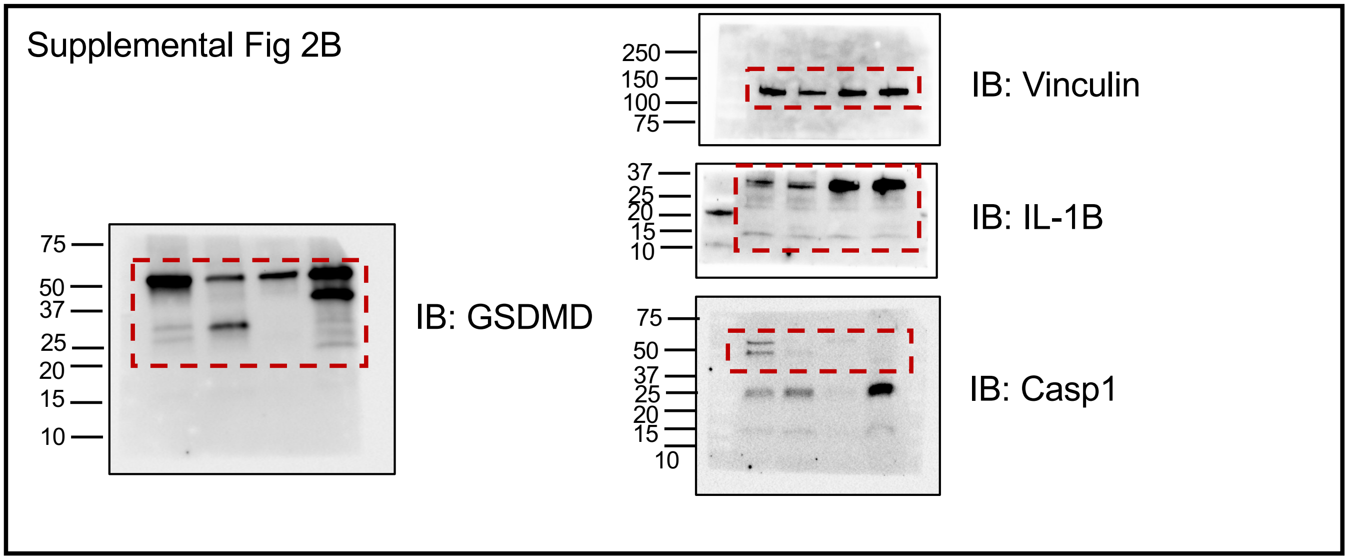

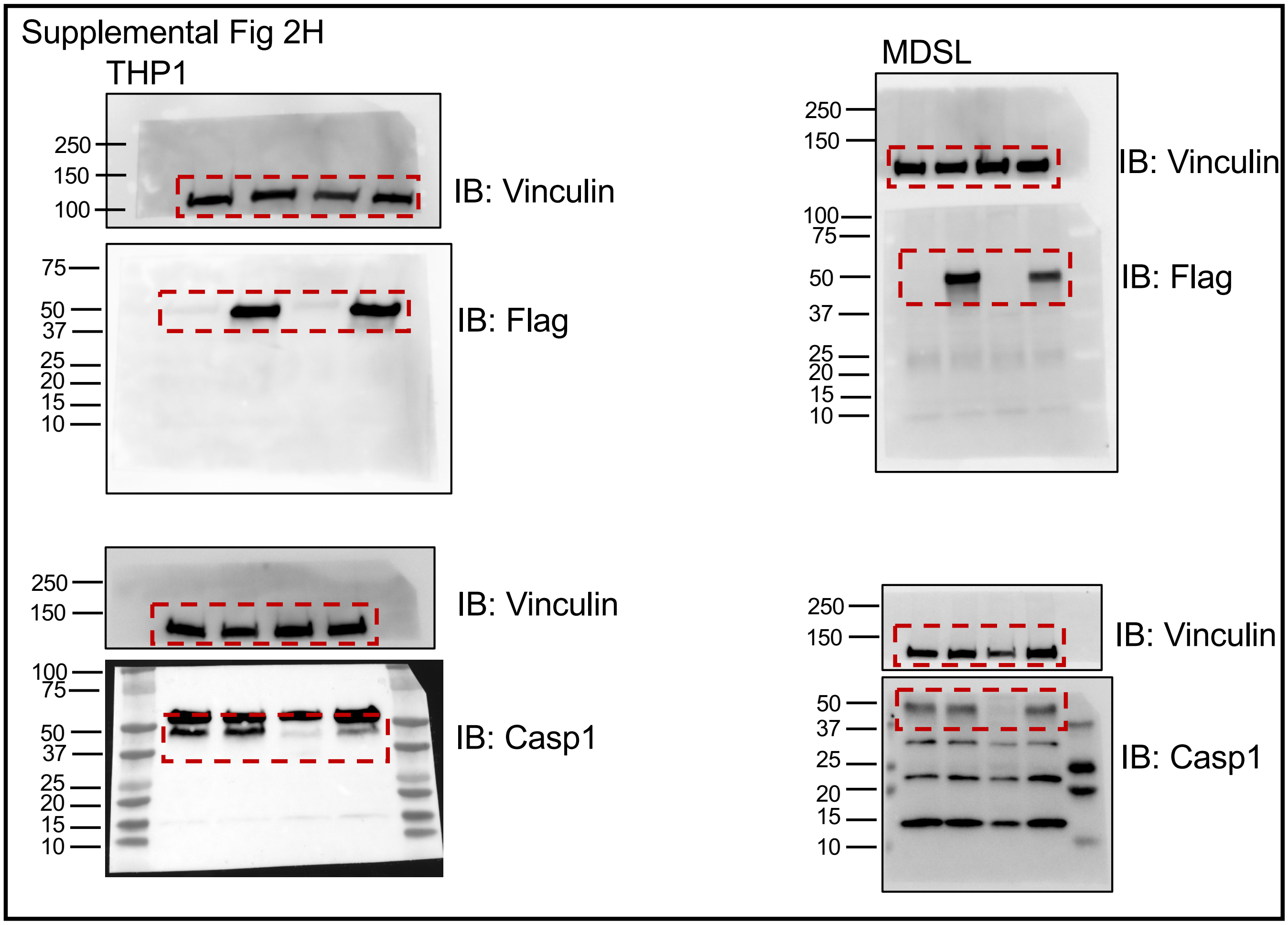

Supplemental Fig 2K

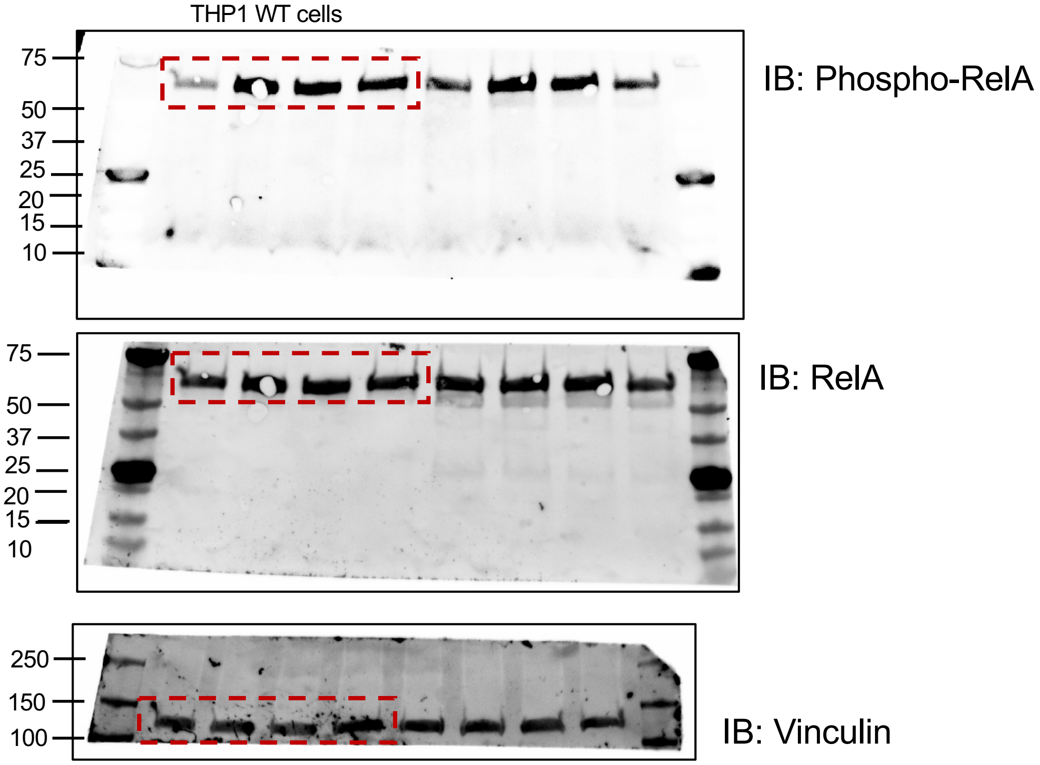

Data S1: Source data, related to Supplemental Figure 3

Supplemental Fig 3A

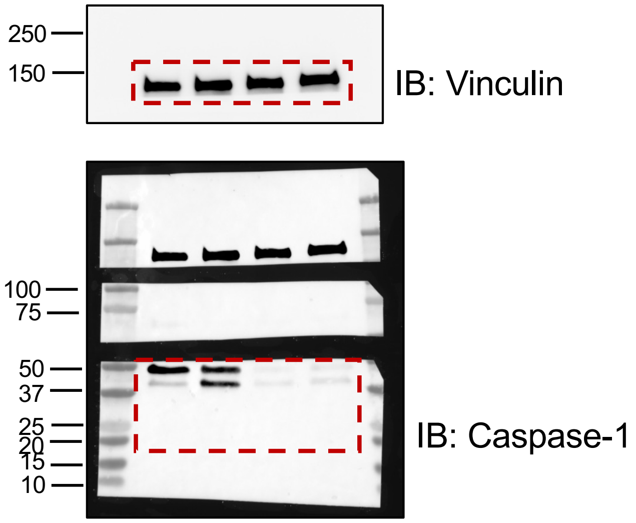

Supplemental Fig 3C

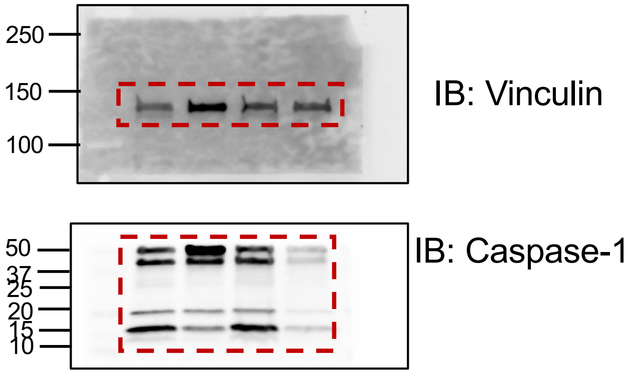

Data S1: Source data, related to Supplemental Figure 4

Supplemental Fig 4F

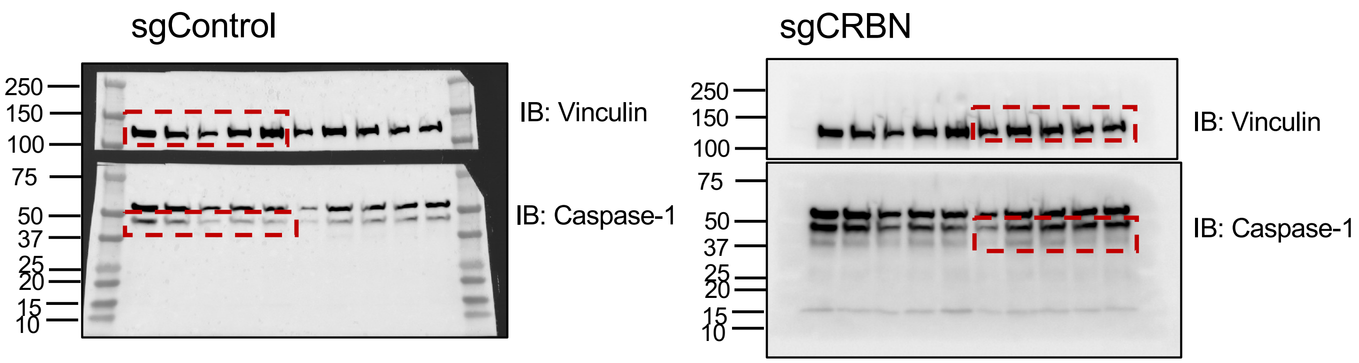

Supplemental Fig 4C

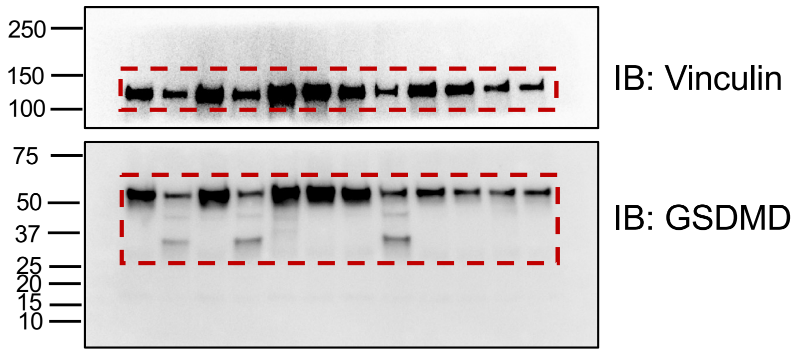

Supplemental Fig 4G

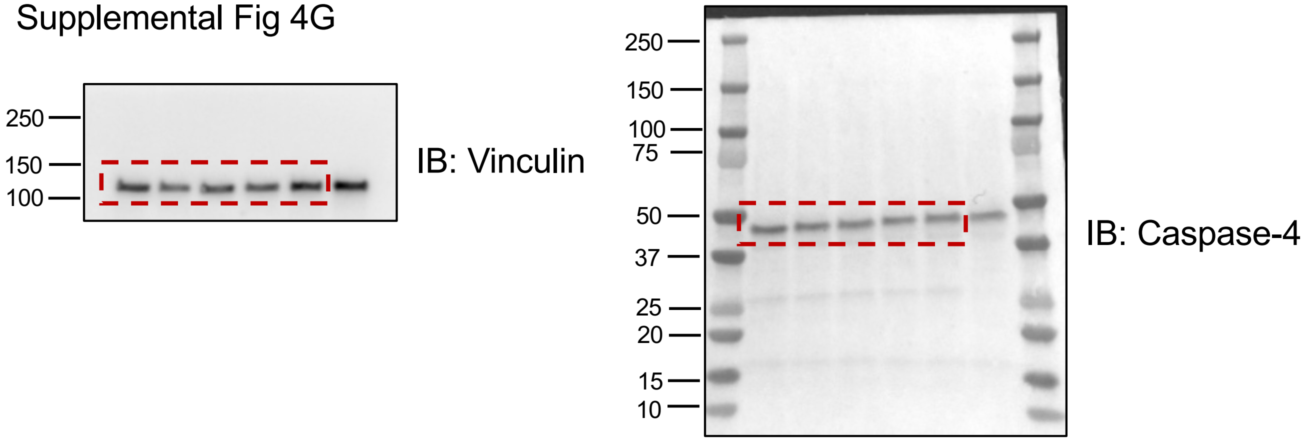

Data S1: Source data, related to Supplemental Figure 5

Supplemental Fig 5A

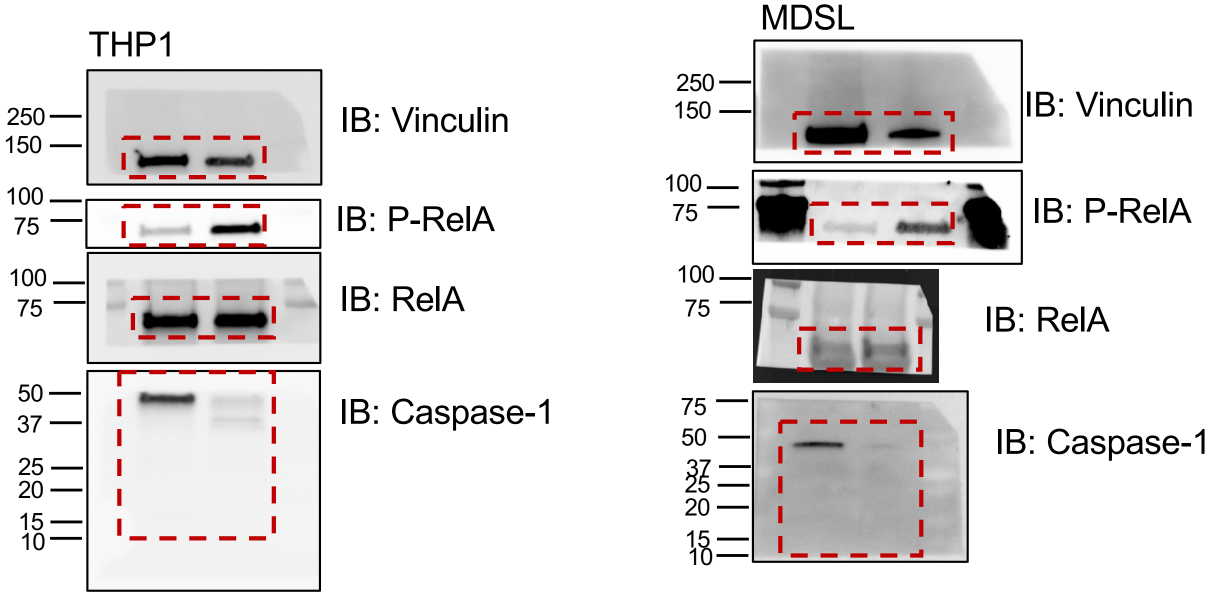

Supplemental Fig 5B

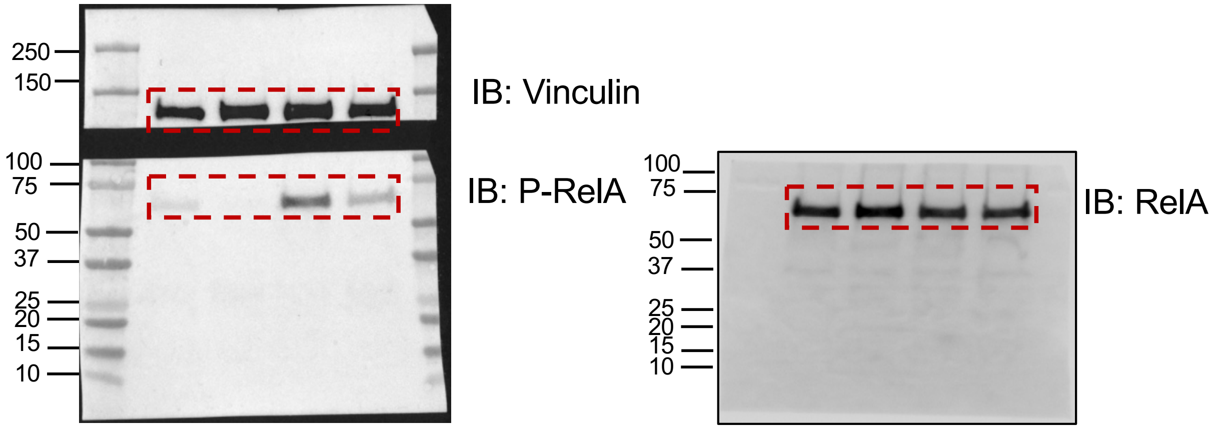

Supplemental Fig 5C

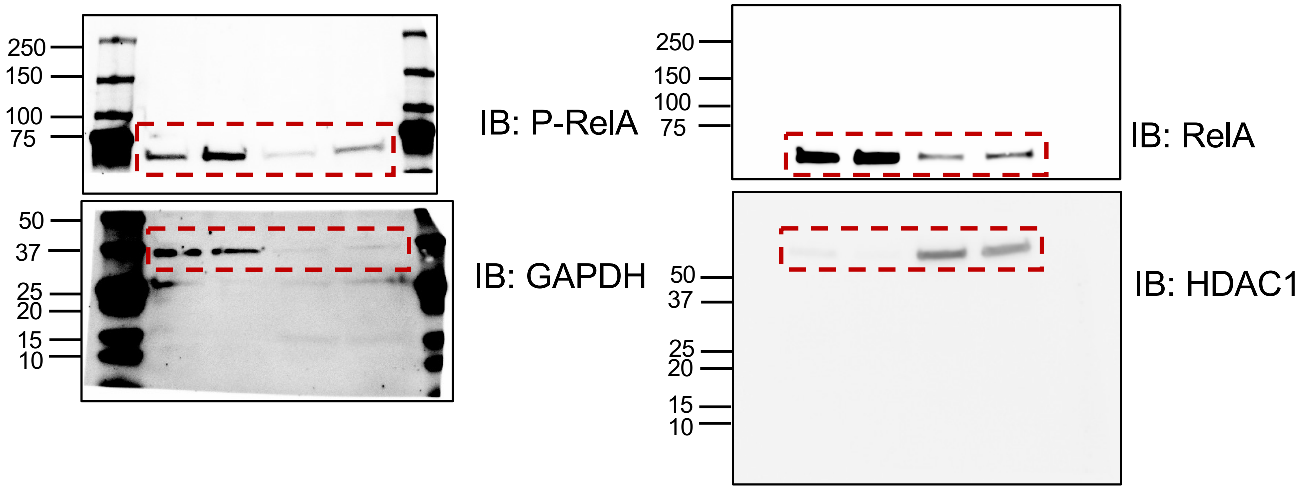

Supplemental Fig 5D

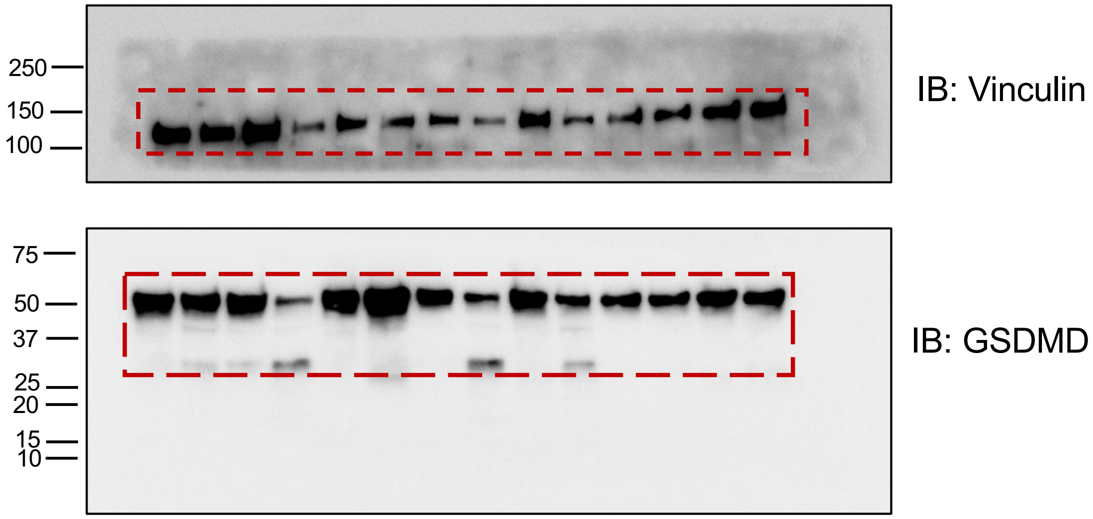

Supplemental Fig 5F

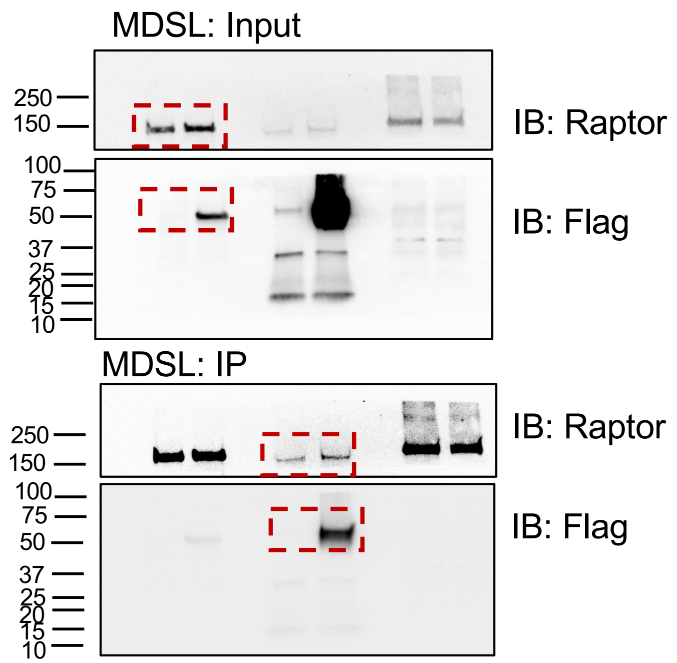

Supplemental Fig 5G

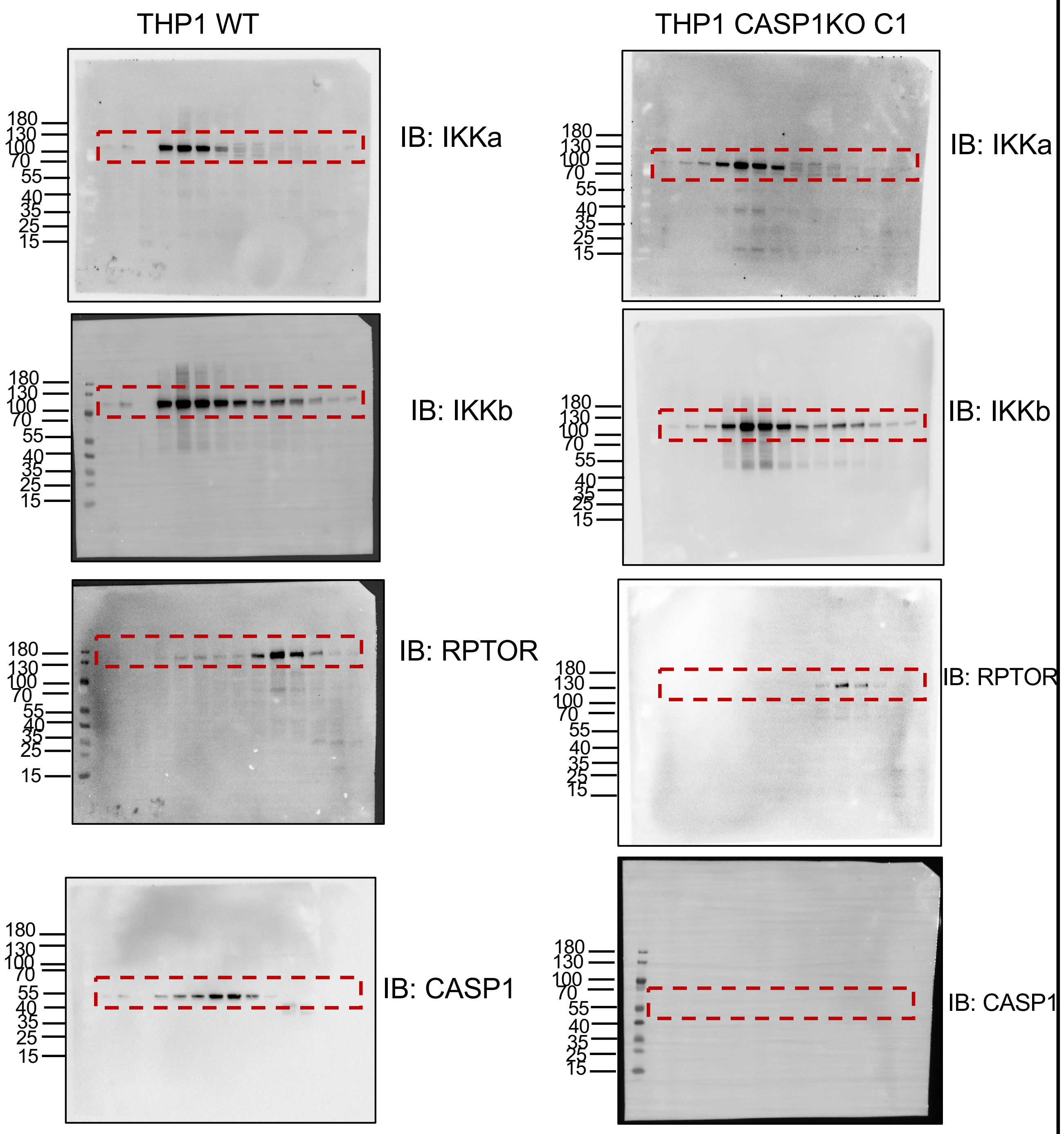

Supplemental Fig 5K

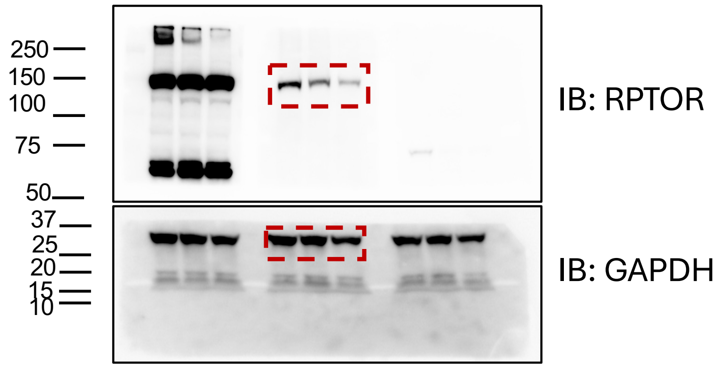

Supplemental Fig 5L

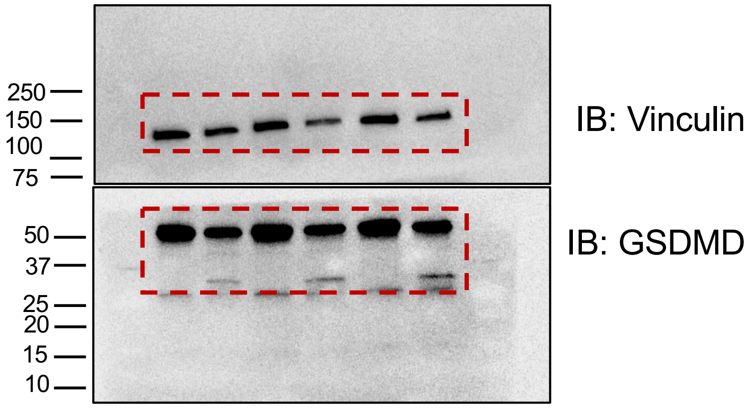

Data S2: Gating strategy, related to Supplemental Figure 2G and 4F

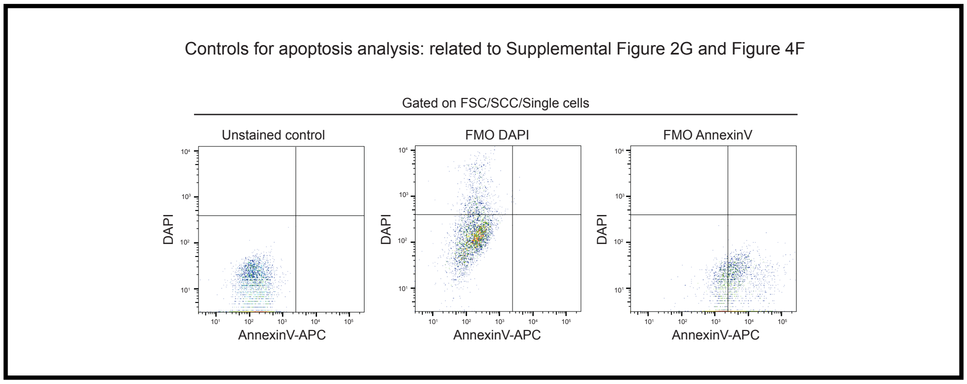

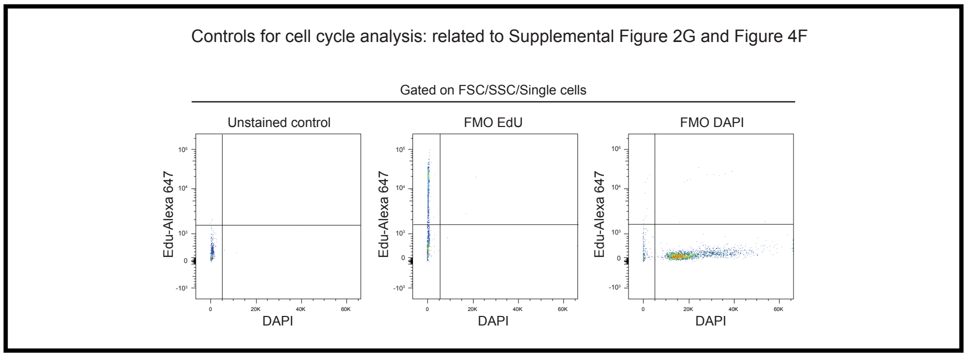

FMO: fluorescence minus one
FSC: forward scatter
SCC: side scatter

Data S3: Chemical synthesis and validation, related to Figure 3A

Synthetic route for dCASP1-55

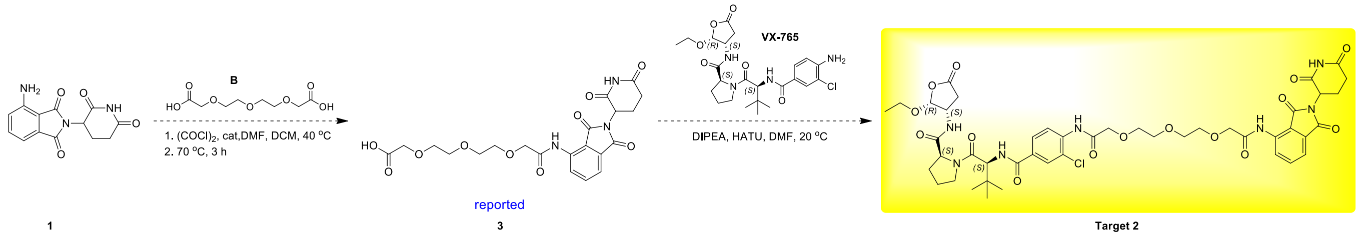

Spectra of dCASP1-55

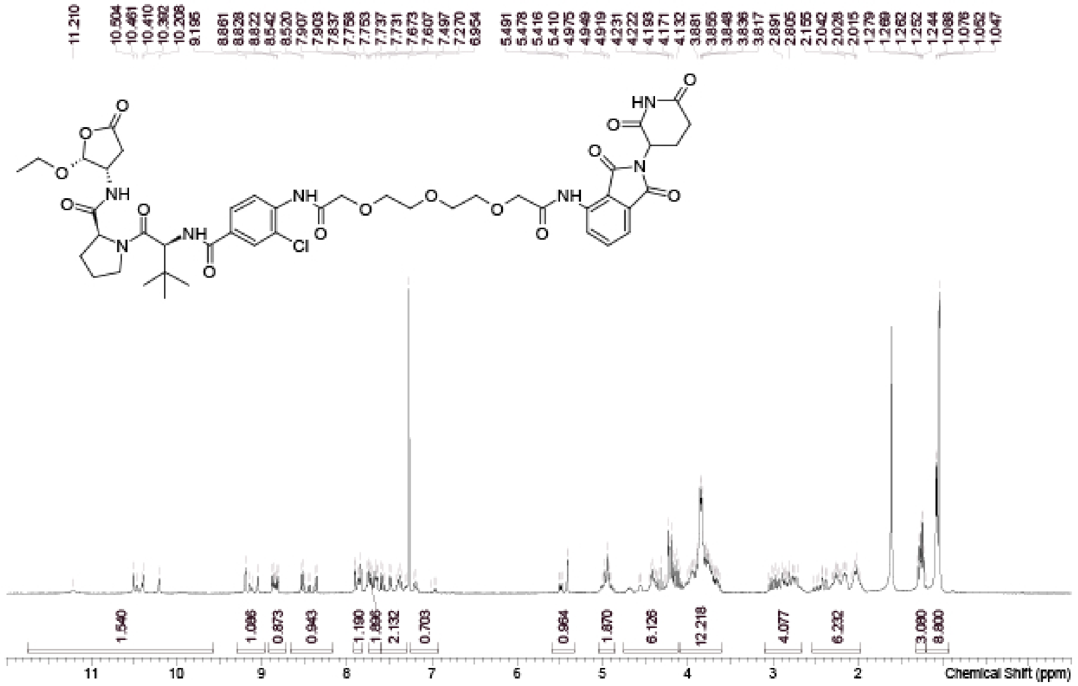

Acquisition Time (sec)	1.9988
Comment	ET94973-8-P1A1 CDCl3 Bruker_02_L_400MHz
Date	12 Sep 2024 15:32:34 (GMT+08:00)
Frequency (MHz)	400.2800
Nucleus	1H
Number of Transients	64
Origin	Avance
Original Points Count	16384
Owner	nmr
Points Count	65536
Pulse Sequence	zg30
Receiver Gain	101.00
SW(cyclical) (Hz)	8196.72
Solvent	CHLOROFORM-d
Spectrum Offset (Hz)	2466.0955
Spectrum Type	standard
Sweep Width (Hz)	8196.60
Temperature (degree C)	25.197

LCMS

LCMS REPORT

Compound ID	:	Target 2
Sample ID	:	ET94973-8-P1Q1
Injection Date	:	11. Sep. 2024
Location	:	P1-D-03
Inj. Vol.	:	3.00 uL
Acq Method	:	D:\DATA\2409\240911 24\5-95CD_6MIN_220-254.M
Data Filename	:	D:\DATA\2409\240911 24\ET94973-8-P1Q1.D
Instrument	:	02-LCMS-CF

Chromatogram

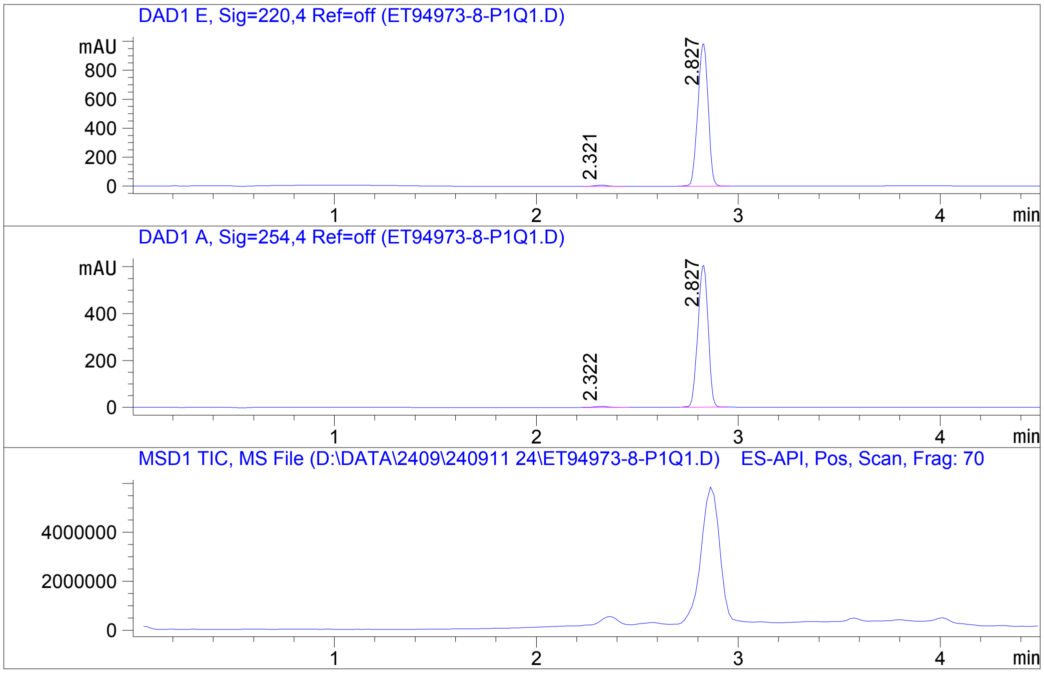

Integration Result

Signal 1 : DAD1 E, Sig=220,4 Ref=off

Peak #	RT [min]	Height	Height %	Width [min]	Area	Area %
1	2.321	8.426	0.845	0.066	34.604	0.983
2	2.827	988.952	99.155	0.055	3487.262	99.017

Signal 2 : DAD1 A, Sig=254,4 Ref=off

Peak #	RT [min]	Height	Height %	Width [min]	Area	Area %
1	2.322	4.466	0.728	0.071	19.500	0.904
2	2.827	608.823	99.272	0.055	2136.877	99.096

MS Spectrum

MS Spectrum

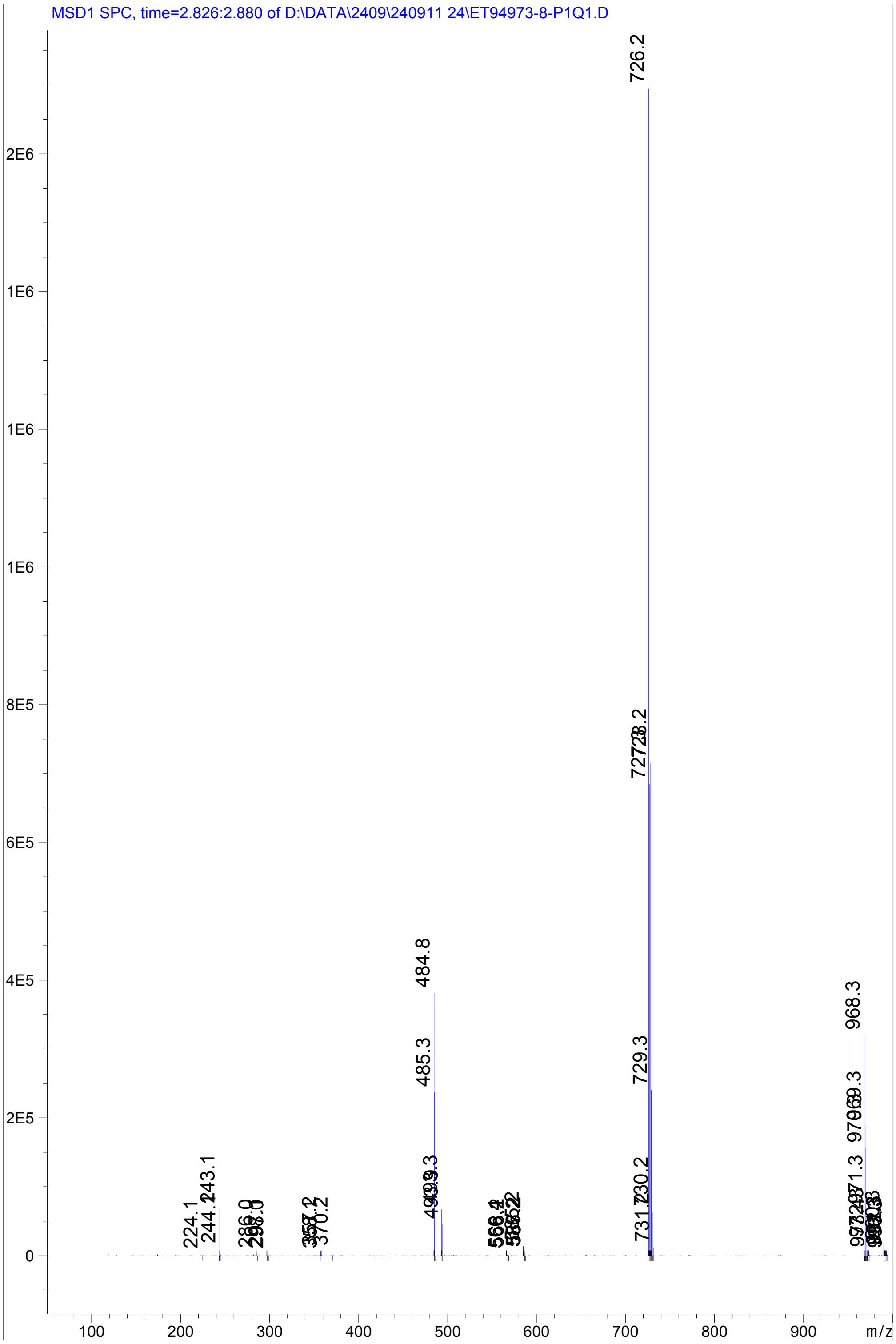

Chiral analytical report

Sample Information

Sample ID:	ET94973-8-P1S1	Acquired By:	System
Compound ID:	Target 2	Sample Set Name:	20240912_N1
Acq Method :	IK_H_E_ACN_IPAm_i50_13_30	Processing Method:	10215
Date Acquired:	9/13/2024 2:16:49 PM CST	Injection Volume:	7.20 ul
Date Processed:	9/13/2024 2:24:18 PM CST	Vial:	2:F,4
Channel Name:	254.0nm	Run Time:	7.0 Minutes
Project Name:	2024\Anal-HPLC-K-20240820	Raw Data:	D:\Data\ID_m32_result_ 27951
User Name:	CASTJ_CA (CASTJ_CA)	Label:	Develop forAnal
Instrument:	CAS-TJ-ANA-Chiral HPLC-K (Waters Arc with 2998)		
Proc. Chnl. Descr.:	2998 PDA 254.0 nm (2998 (190-500)nm)		

Test Results

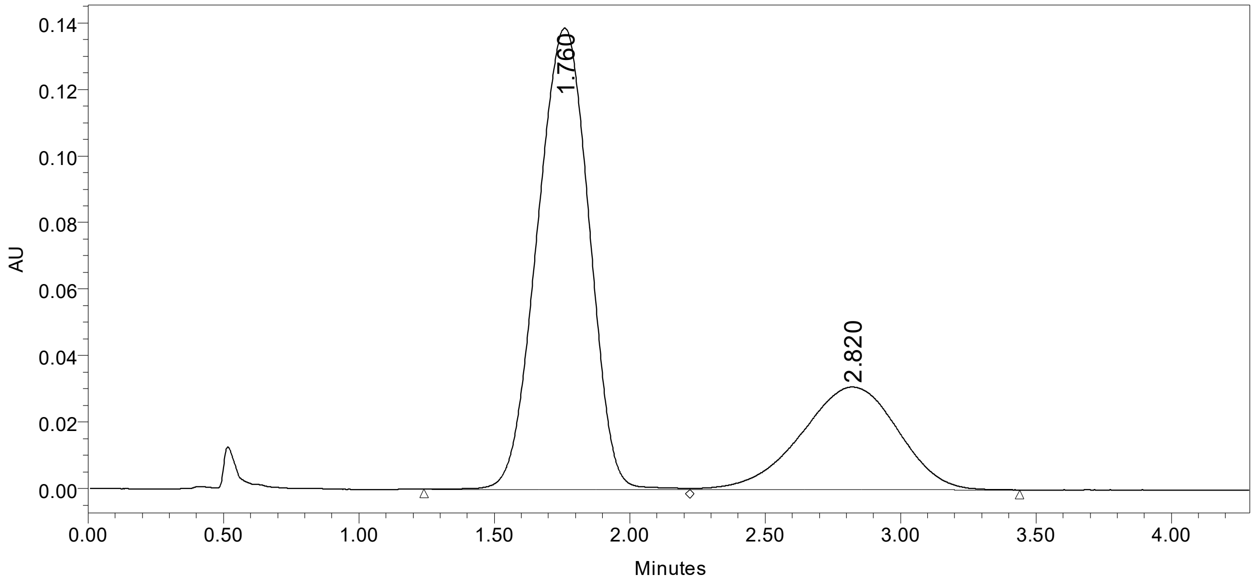

	RT	Area	% Area	Height
1	1.760	1838017	70.40	138698
2	2.820	772864	29.60	30868

Method information

Instrument Method: IK_H_E_ACN_IPAm_i50_13_30

Stored: 8/26/2024 3:35:34 PM CST

Method Information

Method Comments	Column: Chiralpak IK-3, 50×4.6mm I.D., 3um<br>Mobile phase: A: Hexane B: EtoH+ACN(4:1)(0.1%IPAm,v/v<br>Gradient: A:B=50:50<br>Flow rate: 1.3mL/min<br>Column temp.: 30°C
Method Modified User	CASTJ_CA
Method Locked	No
Method Id	8172
Old Id	
Method Version	2
Method Edit User	
Source S/W Info	Empower 3 Software Build 3471 SPs Installed: Service Release 3 DB ID: 292669548[illegible]
