# Supplementary material for: Scaffolding-dependent CASP1 constrains excessive cell-intrinsic inflammatory signaling in leukemia
Source: Cell Chem Biol. Author manuscript; Available in PMC 2026 Jun 28. (PMC13310425; doi:10.1016/j.chembiol.2025.12.002)
Supplement: 1 [file NIHMS2173962-supplement-1.pdf]

Cell Chemical Biology, Volume *33*

Supplemental information

Scaffolding-dependent CASP1 constrains excessive cell-intrinsic inflammatory signaling in leukemia

Emma E. Uible, Issac Choi, Courtnee A. Clough, Aishlin Hassan, Annabelle J. Anandappa, Julianna Fisher, Bibek Karki, Kathleen Hueneman, Kwangmin Choi, Eric J. Vick, William Seibel, Kenneth D. Greis, Lynn Lee, Courtney Jones, Timothy M. Chlon, Jorge Henao-Mejia, Chandrashekhar Pasare, John T. Cunningham, Andrew G. Volk, and Daniel T. Starczynowski

Supplemental information

Scaffolding-dependent CASP1 constrains excessive cell-intrinsic inflammatory signaling in leukemia

Emma E. Uible, Issac Choi, Courtnee A. Clough, Aishlin Hassan, Annabelle J. Anandappa, Julianna Fisher, Bibek Karki, Kathleen Hueneman, Kwangmin Choi, Eric J. Vick, William Seibel, Kenneth D. Greis, Lynn Lee, Courtney Jones, Timothy M. Chlon, Jorge Henao-Mejia, Chandrashekhar Pasare, John T. Cunningham, Andrew G. Volk, Daniel T. Starczynowski

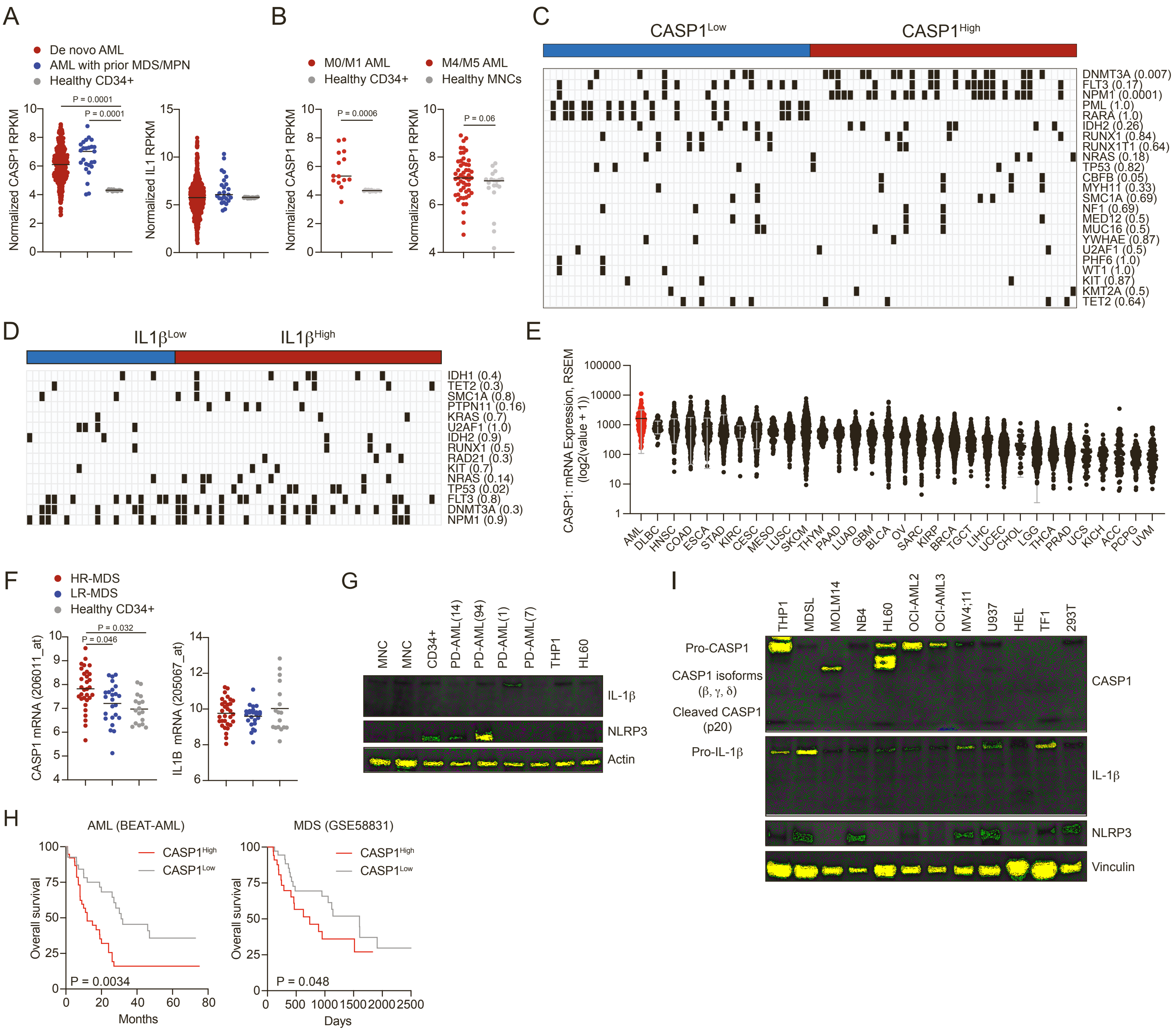

Figure S1. CASP1 expression in human cancer, related to Figure 1. (A) CASP1 (left) and IL-1β (right) expression in patients with de novo (n = 476) and secondary AML after prior MDS/MPN (n = 25), and healthy human CD34+ BM cells (n = 13) from BEAT-AML (left panel). **(B)** CASP1 expression in patients with M0/M1 or M4/M5 AML subtypes and healthy human CD34+ BM cells or BM mononuclear cells (MNCs) from BEAT-AML, respectively. **(C)** Mutations in AML patients from TCGA clustered in Group 1 (low CASP1 expression) and Group 2 (high CASP1 expression). P values were determined with hypergeometric testing. **(D)** Mutations in AML patients from TCGA clustered in Group 1 (low IL-1β expression) and Group 2 (high IL-1β expression). P values were determined with hypergeometric testing. **(E)** Expression of CASP1 mRNA across human cancer subtypes in TCGA. **(F)** CASP1 (left) and IL-1β (right) expression in CD34+ cells from high-risk (HR) (n = 31) and low-risk (LR) MDS patients (n = 24), and healthy human CD34+ BM cells (n= 17) from Gerstung et al, 2015 (GSE58831). **(G)** Immunoblot of the indicated patient samples, cell lines, and healthy CD34+ or MNCs. **(H)** Overall survival of AML patients (left, n = 174), and MDS patients (right, n = 184) stratified on highest CASP1 (top 25%) or lowest CASP1 (bottom 25%) mRNA expression. Mantel-Cox test was used to determine significance. **(I)** Immunoblots of the indicated cell lines.

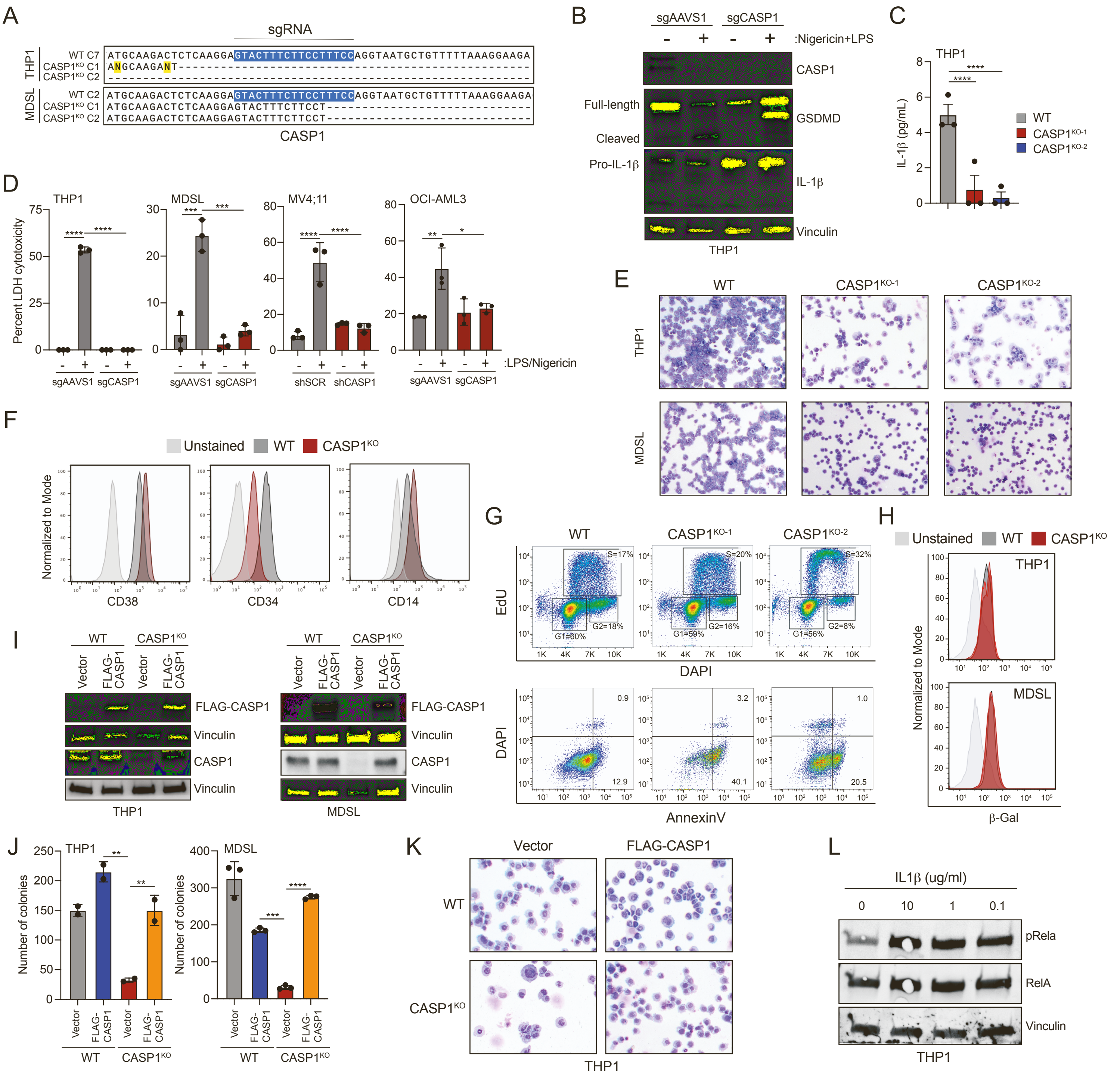

Figure S2. Generation and evaluation of CASP1-deficient leukemic cells, related to Figure 1. (A) Trace of reference genomic DNA sequence (top) and genomic DNA sequences for THP1 and MDSL sgCASP1 clones. **(B)** Immunoblot of WT and CASP1-deficient THP1 cells treated with LPS (24 hours of 10 ug/mL) and Nigericin (2 hours of 20 μM) to activate the inflammasome. **(C)** Protein expression of IL-1β assessed by ELISA in the media of WT and CASP1-deficient THP1 cells. **(D)** LDH release assay was performed on WT and CASP1-deficient cells treated with LPS (24 hours of 10 ug/mL) and Nigericin (2 hours of 20 μM) to activate the inflammasome. **(E)** Wright-Giemsa staining of isogenic WT or CASP1-deficient cell lines. **(F)** Expression of the indicated cell surface markers by flow cytometry on isogenic WT or CASP1-deficient THP1 cell lines. **(G)** AnnexinV and EdU staining of WT and CASP1-deficient THP1 cells. **(H)** β-galactosidase staining of WT and CASP1-deficient THP1 and MDSL cells. **(I)** Immunoblot of WT and CASP1-deficient THP1 and MDSL cells expressing CASP1 (FLAG-CASP1) or empty vector. **(J)** Colony forming assay of WT and CASP1-deficient THP1 and MDSL cells expressing CASP1 (FLAG-CASP1) or empty vector. **(K)** Wright-Giemsa staining of WT and CASP1-deficient THP1 and MDSL cells expressing CASP1 (FLAG-CASP1) or empty vector. **(L)** Immunoblot of THP1 cells treated with increasing concentrations of IL-1β.

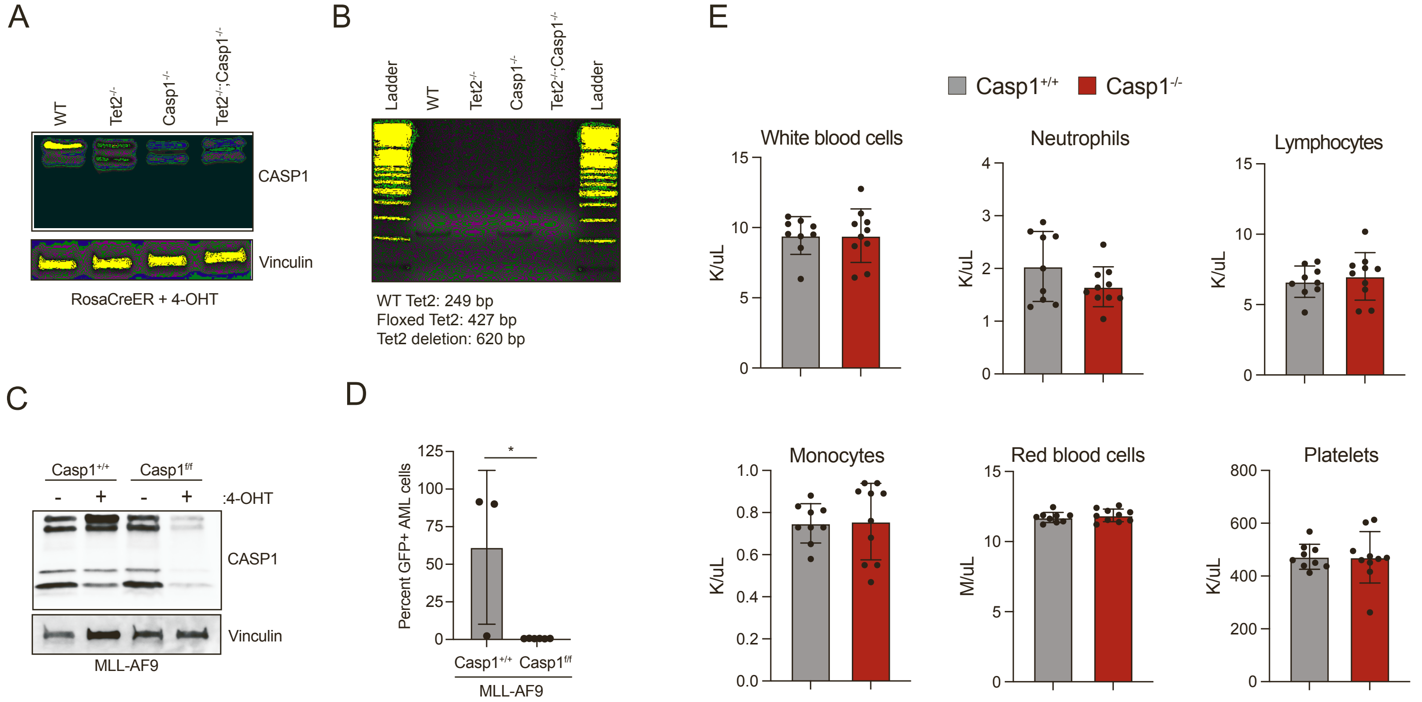

Figure S3. Generation of Casp1-deficient mouse models, related to Figure 1. (A) Immunoblot of cKit+ BM cells isolated from WT;RosaCreER, Tet2$^{f/f}$;RosaCreER, Casp1$^{f/f}$;RosaCreER, and Tet2$^{f/f}$Casp1$^{f/f}$;RosaCreER mice treated with 1 μM 4-OHT for 48 hours in liquid culture. **(B)** Tet2 genotyping of cKit+ BM cells isolated from WT;RosaCreER, Tet2$^{f/f}$;RosaCreER, Casp1$^{f/f}$;RosaCreER, and Tet2$^{f/f}$Casp1$^{f/f}$;RosaCreER treated with 1 μM 4-OHT for 48 hours in liquid culture. **(C)** Immunoblot of MLL-AF9-expressing Casp1$^{f/f}$;RosaCreER and Casp1$^{+/+}$;RosaCreER AML cells treated with 1 μM 4-OHT for 48 hours in liquid culture. **(D)** Leukemic burden in the BM of the indicated mice as determined by GFP expression. **(E)** Peripheral blood counts in recipient mice engrafted with Casp1$^{+/+}$ or Casp1$^{-/-}$ BM cells at 70 days post transplantation.

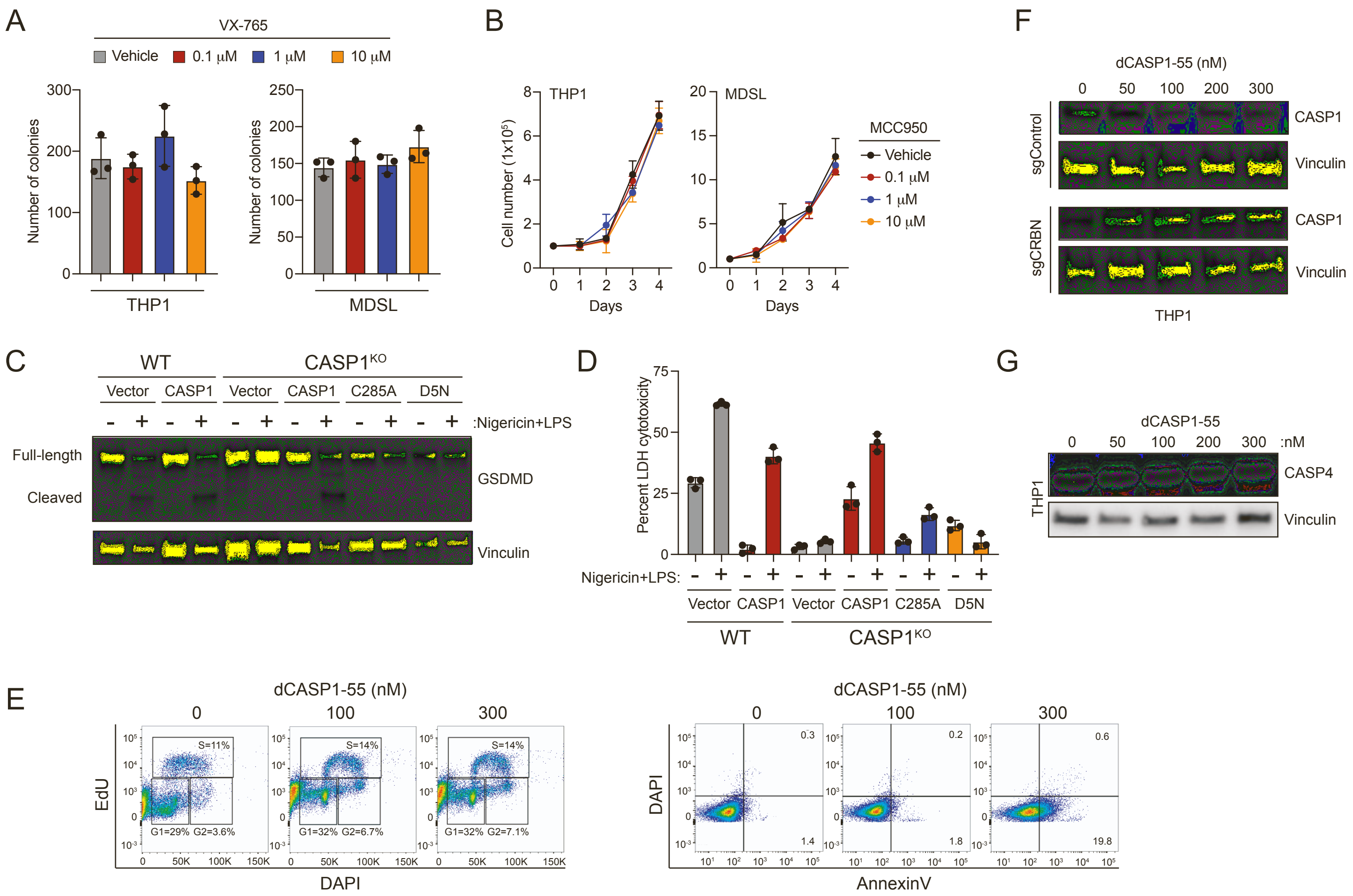

Figure S4. Pharmacological modulation of inflammasome activation and CASP1 degradation in leukemia, related to Figures 2 and 3. (A) Colony formation of THP1 and MDSL cells treated with increasing concentrations of the CASP1 inhibitor (VX-765). **(B)** Proliferation of THP1 and MDSL cells treated with increasing concentrations of the NLRP3 inhibitor MCC950 (n = 3 independent biological replicates). **(C)** Immunoblot of WT and CASP1-deficient THP1 cells expressing the indicated FLAG-CASP1 mutants treated with LPS (24 hours of 10 μg/mL) and Nigericin (2 hours of 20 μM) to activate the inflammasome. **(D)** LDH release assay was performed on the indicated cells treated with LPS and Nigericin. **(E)** AnnexinV and EdU staining of THP1 cells treated with the indicated concentrations of dCASP1-55. **(F)** Immunoblot of Cereblon-deficient (sgCRBN) THP1 cells treated with increasing concentrations of dCASP1-55 to confirm CRBN-dependent degradation of CASP1. **(G)** Immunoblot of THP1 cells treated with increasing concentrations of dCASP1-55. Related to Figure 3B.

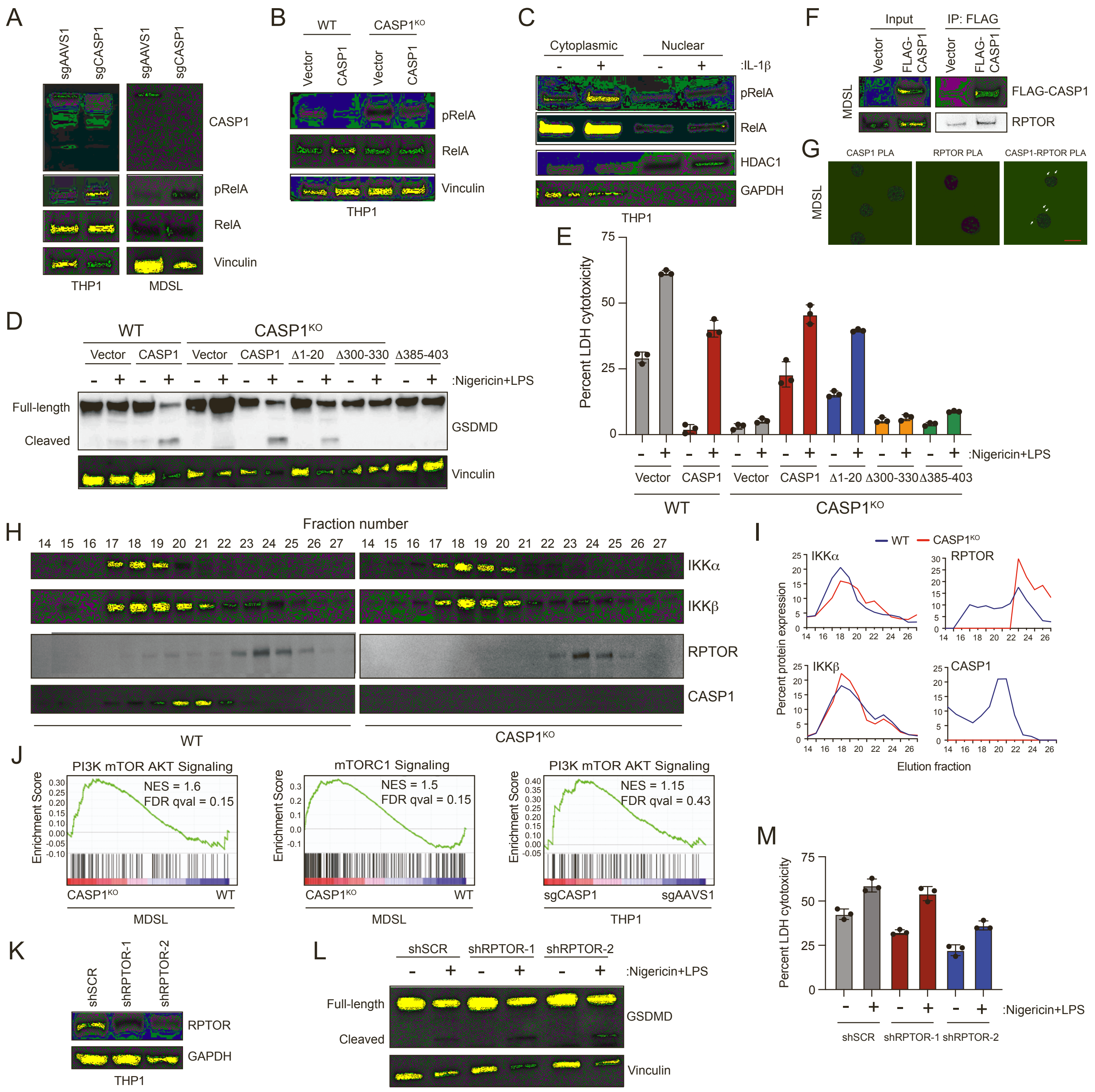

Figure S5. Validation of CASP1 regulation of RPTOR-NF-κB activation in leukemia, related to Figures 4-6. (A) Immunoblot of WT and CASP1-deficient THP1 and MDSL cells used for RNA-sequencing. **(B)** Immunoblot of WT and CASP1-deficient THP1 cells expressing vector and FLAG-CASP1. **(C)** Immunoblot of cytoplasmic and nuclear fractions of THP1 cells treated with IL-1β (10 ng/ml). **(D)** Immunoblot of WT and CASP1-deficient THP1 cells expressing the indicated FLAG-CASP1 mutants treated with LPS (24 hours of 10 μg/mL) and Nigericin (2 hours of 20 μM) to activate the inflammasome. **(E)** LDH release assay was performed on the indicated cells treated with LPS and Nigericin. **(F)** Co-immunoprecipitation (IP) in MDSL cells expressing FLAG-CASP1. **(G)** Representative confocal images of PLA between CASP1 and RPTOR in MDSL cells. As negative controls, CASP1 or RPTOR PLA alone were performed. **(H)** Size fractionation was performed on isogenic WT and CASP1-deficient THP1 cells. The fractionations were analyzed by immunoblotting. **(I)** Densitometry of immunoblotted proteins from each fraction was calculated for WT and CASP1-deficient cells. **(J)** Gene set enrichment analysis of WT or CASP1 deficient THP1 and MDSL cells. **(K)** Immunoblot of THP1 cells expressing non-targeting shRNA (shSCR) or shRNAs targeting RPTOR (shRPTOR). **(L)** Immunoblot of WT and CASP1-deficient THP1 cells expressing shSCR or shRPTOR treated with LPS (24 hours of 10 ug/mL) and Nigericin (2 hours of 20 μM). **(M)** LDH release assay was performed on the indicated cells treated with LPS and Nigericin.
